# Resolving short-term meteorological variability amplifies projected glacier loss and sea-level rise

**DOI:** 10.1038/s41467-026-78260-y

**Published:** 2026-10-19

**Authors:** Lander Van Tricht, Matthias Huss, Harry Zekollari, Rodrigo Aguayo, Janosch Beer, Marit van Tiel, Alexandra von der Esch, Daniel Farinotti

**Affiliations:** 1 Laboratory of Hydraulics, Hydrology and Glaciology (VAW), ETH Zürich, Zurich, Switzerland; 2 Swiss Federal Institute for Forest, Snow and Landscape Research (WSL), Sion, Switzerland; 3 Department of Water and Climate, Vrije Universiteit Brussel, Brussels, Belgium; 4 Department of Geosciences, University of Fribourg, Fribourg, Switzerland; 5 Swiss Federal Institute for Forest, Snow and Landscape Research (WSL), Birmensdorf, Switzerland

**Keywords:** Cryospheric science, Climate change

## Abstract

Global glacier models typically rely on temperature-index mass balance calculations forced with aggregated meteorological data, limiting their ability to resolve short-term variability. Here, we develop a daily-resolution version of the Global Glacier Evolution Model (GloGEM), which we apply globally and compare against the monthly configuration. Our results show that the daily model more closely reproduces mass balance observations and provides a more detailed representation of glacier runoff. Accounting for short-term meteorological variability leads to systematically greater projected mass loss, with up to 25% greater loss in Svalbard. These differences arise from extreme melt days, precipitation-phase transitions, and surface-type feedbacks not fully represented at monthly resolution. Globally, the daily model yields ice volume losses of 26 ± 10% under low-emission and 47 ± 17% under high-emission scenarios over 2025-2100 (ensemble mean ± 1 StD), resulting in an additional 5-10% mass loss (20-33 mm of global mean sea-level rise) relative to the monthly implementation. Resolving high-frequency forcing, or accounting for its effects through parameterisations, is therefore essential for robust glacier projections, while its omission may systematically underestimate future glacier loss.

## Abstract (web summary)

Accounting for daily weather variability and extremes in global glacier models better captures melt, snowfall and surface feedbacks, revealing greater projected glacier loss and sea-level rise.

## Introduction

Glaciers outside the ice sheets lost 273 ± 16 Gt of ice per year between 2000 and 2023^1^, contributing 18.1 ± 0.9 mm to global mean sea-level rise. This represents approximately 20–25% of the observed sea-level rise over this period, making glaciers the second-largest contributor after ocean thermal expansion^2^. Beyond their role as contributors to sea-level rise, glaciers are critical freshwater reservoirs for nearly 10% of the global population^3^. Ongoing and projected glacier loss is expected to diminish meltwater supply^4–6^, alter the frequency and severity of glacier-related hazards, such as outburst floods and ice mass collapses^7–9^, affect tourism economies^10^, and intensify pressures on sensitive high-altitude ecosystems^11,12^. Robust glacier modelling is therefore essential for projecting these impacts.

Global-scale glacier projections under the most recent climate scenarios suggest that glaciers could lose 25–29% of their global 2015 volume by 2100 under low-emission scenarios, and 46–54% under high-emission scenarios^13,14^, slightly exceeding earlier estimates^15^. To date, most state-of-the-art global glacier models^16–18^ used for large-scale projections^15,19,20^ are forced with aggregated monthly meteorological data. While a number of large-scale glacier evolution models have been forced with daily data^21–24^, these applications have so far focused on selected regions or river basins, individual scenarios, or limited glacier samples. Daily forcing has not yet been applied in a global glacier model to produce scenario-based projections of 21st-century glacier change. When forced with monthly data, glacier models do not explicitly resolve short-term meteorological variability, including extreme melt during heatwaves^25^, precipitation-phase transitions near 0 °C^26^, intense precipitation events^27^, or rapid surface-type transitions^28^. As climate change increases the frequency and intensity of extremes^29–31^, accounting for the influence of such short-lived variability on glacier mass balance and downstream runoff is becoming increasingly important. This need is further amplified by the growing integration of glacier evolution models within land-surface and hydrological frameworks^22–24,32–35^, which typically require daily or sub-daily inputs.

To examine how accounting for short-term meteorological variability impacts global glacier projections, we develop a daily-resolution version of the Global Glacier Evolution Model (GloGEM^16^; see “Methods”). The daily and original standard (“monthly”) configurations are applied to all glaciers larger than 5 km^2^, which account for ~94% of the global glacier volume (Supplementary Table 1). After evaluating both approaches against mass-balance observations, we analyse the differences in ice-volume and glacier-runoff projections, unravel the underlying regional patterns and physical drivers, and quantify the implications for global sea-level rise projections.

## Results and discussion

### Improved mass balance representation through daily glacier modelling

In our daily configuration (hereafter, “daily model”), GloGEM resolves surface mass balance at a daily time step using daily temperature and precipitation. In contrast, in the standard monthly GloGEM configuration, surface mass balance is computed from monthly mean temperature and total monthly precipitation, with precipitation phase based on monthly temperature and melt estimated using a statistical approach that represents sub-monthly temperature variability using the monthly standard deviation of daily temperatures derived from historical data^16,36^ (see “Methods”). While this monthly formulation (hereafter, “monthly model”) allows melt to occur in months with mean temperatures below 0 °C, it does not explicitly resolve daily temperature variability (Supplementary Fig. 1).

**Fig. 1:**
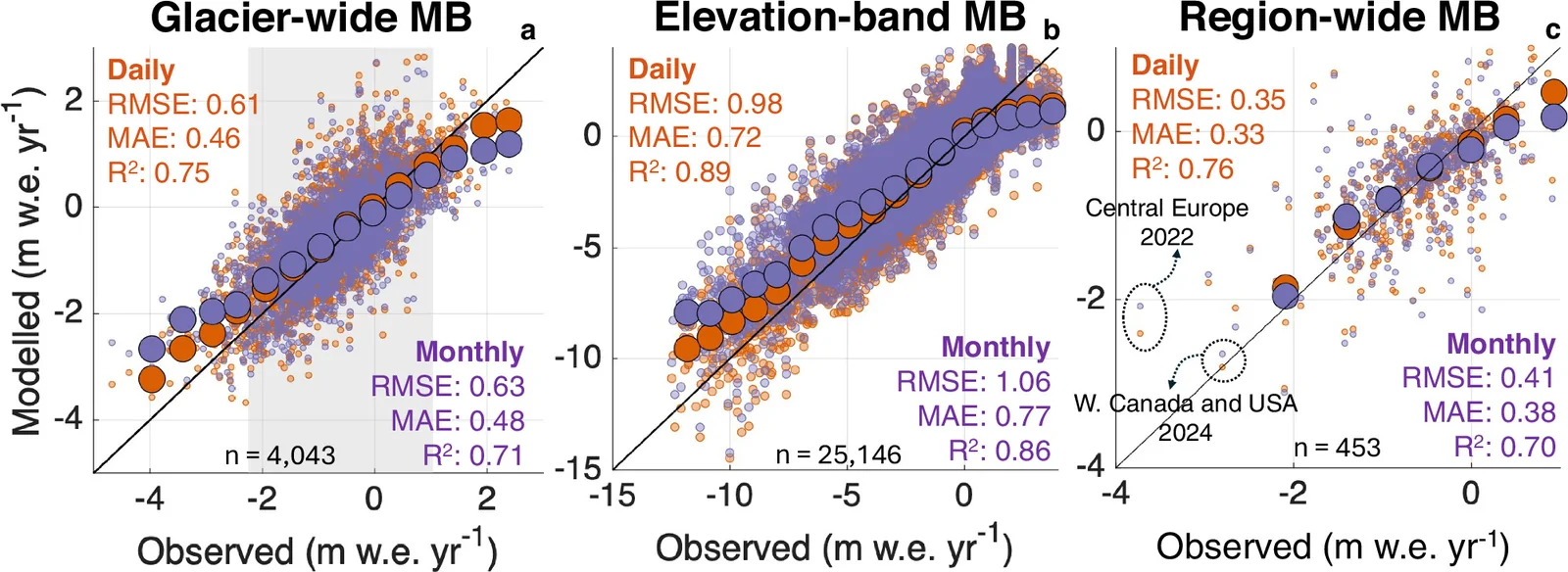
Comparison of modelled and observed glacier mass balance (MB) for the daily and monthly models using the cross-calibrated parameter configuration. **a** Modelled versus observed glacier-wide annual specific mass balance over the period 1980–2024^37^. Only glaciers with at least three years of observations are included (175 glaciers). The larger circles show the mean mass balance within bins of 0.5 m w.e. to better isolate the signal. The grey area shows the mean observed mass balance ±2σ. The number (n) of comparisons is indicated at the bottom of each panel. **b** As in panel **a**, but evaluated across glacier elevation bands. **c** Mean annual modelled region-wide mass balance compared to observations between 2000 and 2024^1^. The comparison relies on observational estimates that include all glaciers, whereas our simulations are restricted to glaciers larger than 5 km^2^. The larger circles show the mean within bins of 0.5 m w.e., where more than three values are available.

We first evaluate model performance by comparing annual climatic mass balances from the daily and monthly models with 4037 independent glacier-wide annual in situ observations^37^. For this evaluation, all glaciers with available observations are included, rather than restricting the analysis to the >5 km^2^ subset used for the global projections. In general, the daily model shows a slight improvement over the monthly model with the root mean squared error (RMSE) decreasing from 0.63 to 0.61 m w.e., the mean absolute error (MAE) decreasing from 0.48 to 0.46 m w.e., and the coefficient of determination (R^2^) increasing from 0.71 to 0.75 (Fig. 1a). These skill scores also represent an improvement over earlier evaluations of GloGEM^16^ (RMSE: 0.73 m w.e), which were based on fewer glaciers, shorter records, and lacked glacier-specific calibration data^14^. At the regional scale, however, improvements are not uniform (Supplementary Fig. 2 and Supplementary Table 2). Most regions show similar or improved performance for the daily model, while others show little change or slightly degraded skill metrics, reflecting regional calibration uncertainties, differences in climate regimes, and limited observational coverage. Regions with sparse mass-balance observations, such as the Russian Arctic and New Zealand, provide only limited opportunities for robust model evaluation, highlighting the continued need for long-term in situ observations in underrepresented glacier regions.

Given that uncertainties in annual WGMS mass-balance observations^38^ are typically of the order of ~0.2-0.4 m w.e. yr^−1^, the improvement in overall RMSE and MAE should be regarded as modest. The added value of the daily model becomes more apparent in extreme years. For mass-balance anomalies outside  ±2σ from the mean, the RMSE decreases from 0.98 to 0.83 m w.e, and the MAE decreases from 0.81 to 0.64 m w.e, when moving from the monthly to the daily model. This indicates a substantially improved representation of both strongly negative and strongly positive mass balance years, driven by enhanced sensitivity to short-term meteorological extremes and steeper mass balance gradients (Supplementary Fig. 3).

Similar improvements are found for mass balances aggregated over elevation bands and for regional mass changes (Fig. 1b, c). The higher RMSE for the elevation-band values is expected, as these data provide a more detailed test of the vertical mass-balance distribution, whereas calibration is primarily constrained by glacier-wide geodetic mass balances (see “Methods”). For the regional comparison, differences in spatial coverage should be kept in mind, as our simulations are restricted to glaciers >5 km^2^ while the GlaMBIE estimates^1^ represent the full regional glacier inventory. Nevertheless, the improvement with daily rather than monthly forcing remains apparent, particularly for extreme mass-balance years.

Since both our daily and monthly glacier models are calibrated against glacier-specific geodetic mass balances for 2000–2019^39^, they are expected to produce nearly identical cumulative mass changes over this period. However, the daily model captures interannual variability more accurately, particularly during extreme years. In the European Alps, for instance, which were subject to several extreme melt years over the past two decades^25,40^, switching from monthly to daily forcing reduces the RMSE of regional mass balances over 2000–2024 from 0.48 to 0.42 m w.e. compared to GlaMBIE observations^1^. During the record Alpine melt year of 2022, the daily model simulates a mass balance that is 0.31 m w.e. more negative than the monthly model, which is substantially closer to observations (Fig. 1c). Comparable improvements for the daily model are found for other recent extremes, including daily-monthly differences of 0.15 m w.e. in Western Canada and the USA in 2023^41^ and up to 0.98 m w.e. in Svalbard in 2024^42^. In high-latitude regions, the standard monthly model configuration of GloGEM shows strong biases during extreme melt years (see also the Svalbard region in Supplementary Fig. 2), highlighting the importance of resolving short-lived but climatically decisive events that are smoothed when using temporally aggregated forcing.

### The role of daily modelling in glacier runoff timing and composition

Glacier runoff integrates mass-balance processes over time and represents the primary pathway through which glacier change affects downstream hydrological systems. Modelled runoff therefore forms the main interface between glacier models and hydrological and land-surface models, which typically operate at (sub-)daily time steps^32,43^. Building on the mass-balance differences and variability identified above, we assess how temporal resolution influences modelled glacier runoff timing and composition. Glacier runoff is defined as the sum of ice melt, snow melt and liquid precipitation over the initially glacierised area^44^, minus refreezing (see “Methods”). We assume that, once generated, all surface water runs off immediately, without temporary storage or delayed release.

**Fig. 2:**
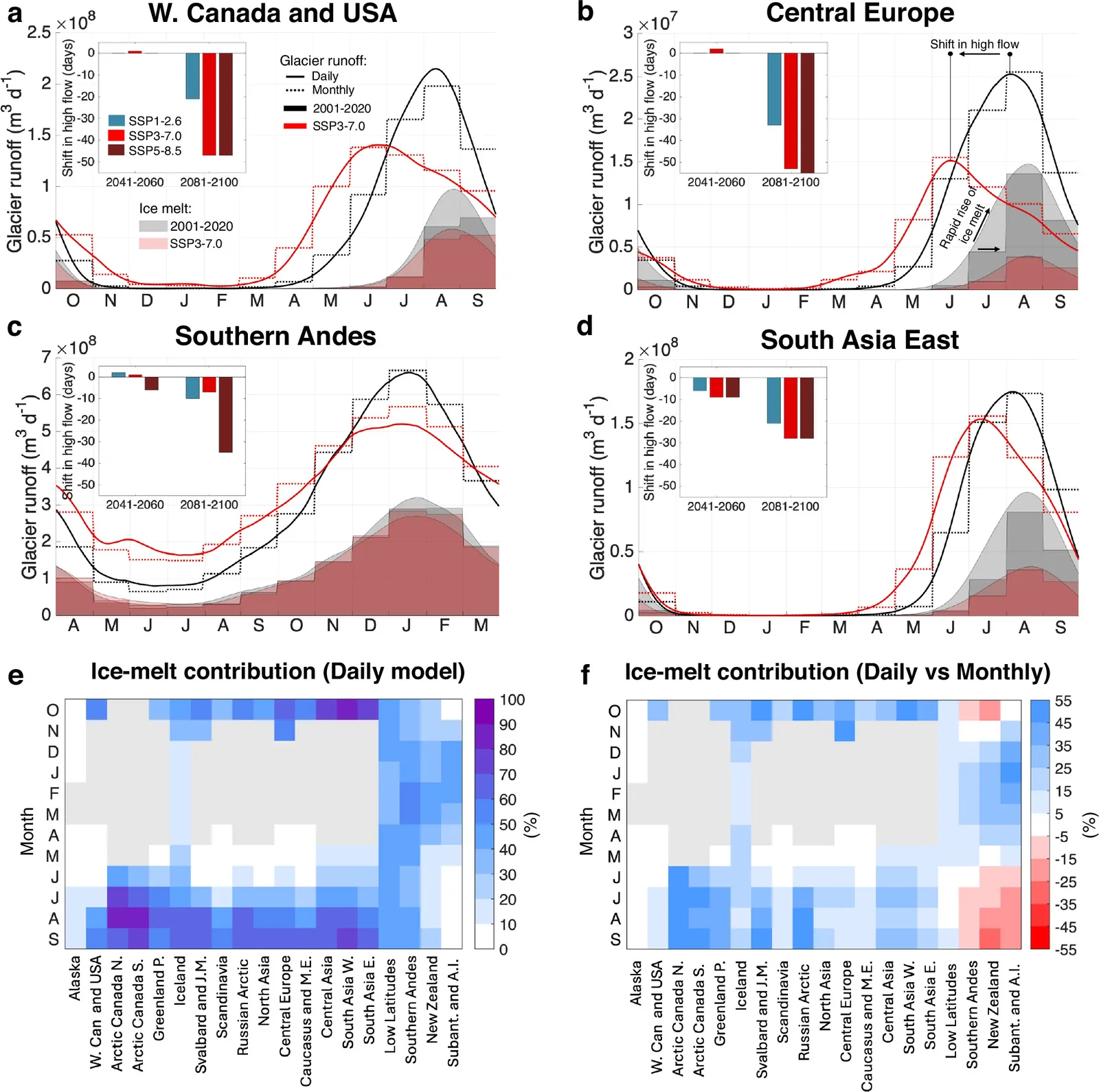
Evolution and variability of glacier runoff as unravelled through daily modelling. **a–d** Modelled mean glacier runoff at daily (solid lines) and monthly (dashed lines) resolution for four climatologically distinct regions (panels) (other regions in Supplementary Fig. 6). Shaded areas indicate the ice-melt component of runoff at present-day (grey, 2001–2020) and 2081–2100 under SSP3-7.0 (red). Insets in the top left show shifts in the timing of the seasonal peak (in days) for the daily model compared to the 2001–2020 baseline. **e** Seasonal cycle of the ice-melt contribution (%) as modelled by the daily model across all RGI regions. Months for which the runoff is <1% of the maximum monthly runoff are greyed out to avoid showing excessively large ice-melt contributions for periods with minimal glacier runoff. **f** The difference in contribution of ice-melt compared to the monthly model (%).

In our monthly model, glacier runoff is assumed to be distributed uniformly within each month, resulting in stepped runoff curves that obscure short-term variability (Fig. 2a–d, other regions in Supplementary Fig. 6). In contrast, the daily model explicitly resolves sub-monthly variability in glacier runoff, producing a continuous seasonal cycle (Fig. 2a–d). This enables identification of the timing of high flow (annual maximum) and its temporal evolution. By 2100, for example, the daily model projects high-flow glacier runoff in Western Canada and the USA to occur ~46 days earlier under both Shared Socioeconomic Pathway (SSP) 3–7.0 and SSP5-8.5, relative to the 2001–2020 historical baseline (Fig. 2a). In the Southern Andes, high-flow timing shifts by ~10–40 days earlier, depending on the scenario (Fig. 2c). The monthly model misrepresents these shifts by approximately 15 days. Furthermore, the daily model captures pronounced day-to-day fluctuations in glacier runoff associated with hydrological extremes (e.g., heatwaves and cold spells; see the example for Central Europe in Supplementary Fig. 7b), which are smoothed out in our monthly configuration.

**Fig. 3:**
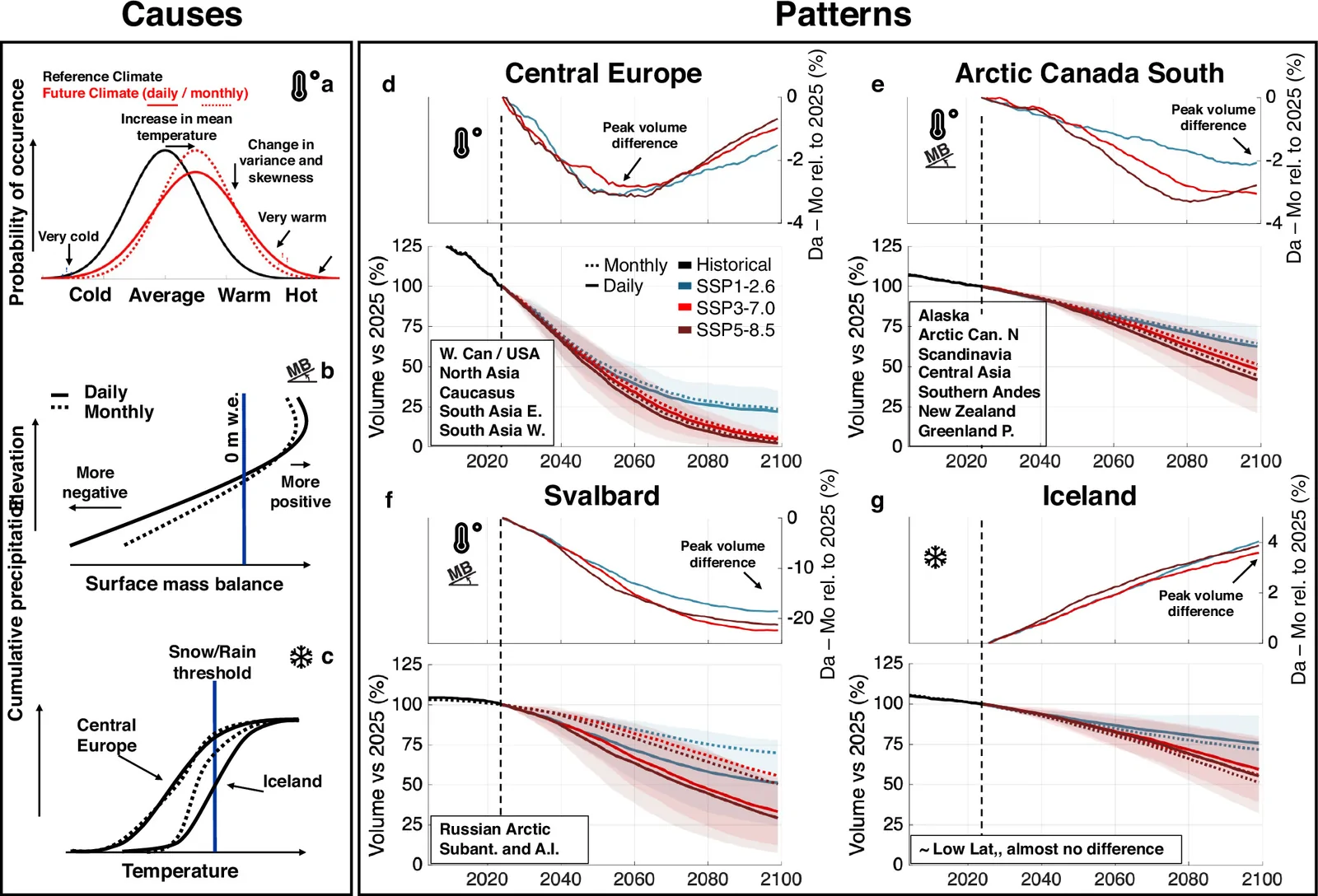
Differences in regional glacier volume projections arising from the transition from monthly to daily modelling, and their underlying drivers. **a–c** Key processes driving the regional contrasts. The symbols next to each panel letter are repeated in panels **d–g** to indicate the dominant drivers of the projected daily–monthly differences. The snowflake icon is taken from https://github.com/siemens/ix-icons. Copyright © 2019–2025 Siemens AG. This project is MIT licensed. The temperature icon is taken from Dazzle UI, Dazzle Line Icons collection (CC Attribution License; SVG Repo). **d–g** Glacier volume projections for four representative regions, each exemplifying a distinct divergence between outputs from the daily and monthly models (regions listed in the boxes in the bottom left follow the same pattern, shown in Supplementary Fig. 9 for every region). In each panel, the lower half represents the multi-GCM mean, and the coloured areas represent ±1 standard deviation, indicating the ensemble spread across GCMs. The upper half highlights the difference in modelled volume between the daily and monthly models, with Da − Mo denoting daily minus monthly simulations and values expressed relative to the 2025 volume.

**Fig. 4:**
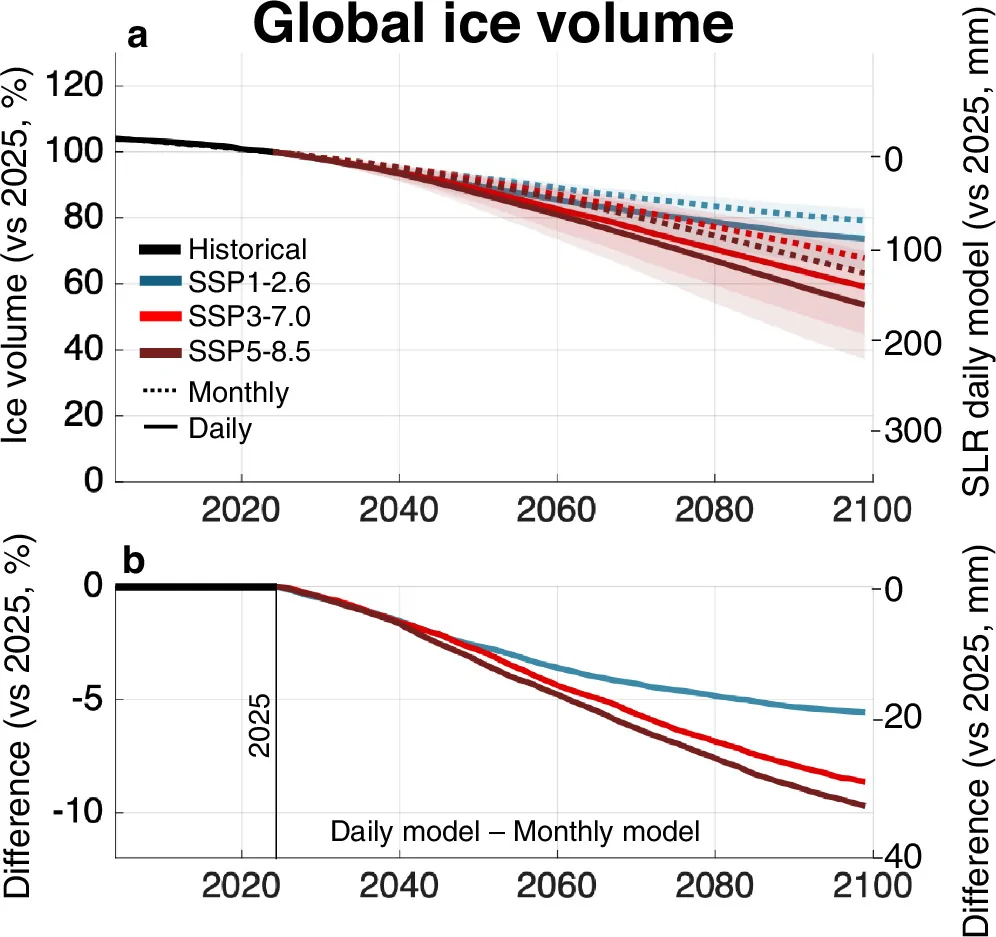
Modelled global ice volume evolution, contribution to sea-level rise, and differences between the daily and monthly models. **a** 21^st^-century ice volume evolution under different scenarios and the corresponding contribution to sea-level rise (multi-model mean ±1 standard deviation). **b** Relative difference in ice-volume loss and absolute difference in contribution to sea-level rise since 2025 between the daily and monthly models .

The temporal resolution also affects the glacier-runoff composition through differences in the frequency at which the surface type is updated within the model. In the daily model, surface transitions (for example, from snow to ice) can occur from day to day  in response to melt or snowfall. In contrast, in the monthly GloGEM model, the surface type remains fixed for each month. This generally reduces the modelled summer ice melt contributions in early summer and increases them in latesummer (Fig. 2a–d and Supplementary Fig. 6). Although projected ice melt over the coming decades is therefore lower in the monthly model in many regions, the larger ice volumes remaining by the end of the century (Figs. 3 and 4) eventually lead to greater glacier runoff and ice-melt contributions than in the  daily model. As for glacier mass balance, resolving daily meteorological variability leads to stronger interannual variations in simulated glacier runoff. In Central Europe, runoff during the extreme melt year 2022 is 8% higher in the daily model than in the monthly model, whereas runoff is 12% lower during the relatively cool year 2014 (Supplementary Fig. 7a). Across 2000–2024, the median annual daily-minus-monthly runoff difference is −2%, with a standard deviation of 4.5%. These contrasts show that both runoff magnitude and timing are sensitive to year-to-year climate variability and to an explicit representation of sub-monthly processes, especially during extreme years.

Finally, the explicit separation of snow melt, ice melt and rainfall at daily resolution enables a detailed assessment of the seasonal evolution of glacier-runoff components (Fig. 2). While a similar decomposition is possible in the monthly configuration, interpretation in the latter case is constrained by temporal averaging and the assumed fixed within-month surface states. For instance, for High Mountain Asia (RGI regions 13–15), the daily model shows that ice melt accounts for approximately 70–80% of present-day glacier runoff by late summer (September), whereas the monthly model yields a substantially lower contribution of 44%. In high-latitude regions, ice-melt contributions are much larger in the daily configuration (Fig. 2f). These component-specific runoff estimates are intended as model-based diagnostics of temporal-resolution effects, rather than as directly validated regional partitioning estimates.

### Regional and global differences in projected ice volume changes

Beyond its influence on mass balance and runoff variability, the temporal resolution also affects the long-term evolution of projected ice volume (Fig. 3, Supplementary Fig. 9, and Supplementary Table 3). These volume differences mainly arise from differences in climate forcing and model structure, which first affect the simulated surface mass balance (Supplementary Fig. 8) and then accumulate through time into differences in glacier geometry, area and volume. At the regional scale, differences in the 21^st^-century ice-volume evolution between the daily and monthly models can be grouped into four main patterns (Fig. 3d–g). These patterns consist of differences in timing, persistence, and magnitude of ice volume evolution between the two configurations and mainly reflect regional contrasts in climate variability, glacier hypsometry and elevation range. We refer to these patterns below as (1) transient divergence, with convergence towards deglaciation, (2) persistent divergence in regions with sustained ice cover, (3) amplified divergence in low-relief ice-cap regions, and (4) reversed divergence associated with precipitation-phase effects.

The first pattern is characterised by a transient divergence in ice volume between the daily and monthly models that peaks around mid-century and declines afterward (Fig. 3d). This behaviour is observed in mid-latitude regions with relatively limited present-day ice volumes, such as North Asia, Central Europe, and the Caucasus. In these regions, the daily model typically projects 1–4% greater ice loss by 2050 (relative to 2025), a difference that primarily reflects more negative mass balances under climate change, resulting in accelerated glacier loss (Fig. 3a). However, differences tend to decline thereafter due to rapid deglaciation. Projections from both the daily and the monthly models converge toward near-zero ice volumes by 2100.

The second pattern appears for regions that retain a substantial ice volume throughout the 21^st^ century and therefore exhibit a persistent and, in some cases, increasing divergence between the daily and monthly models (Fig. 3e). This behaviour is observed in regions such as Alaska, Scandinavia, and the Southern Andes, where resolving daily variability produces steeper mass-balance gradients (Supplementary Fig. 3). The latter leads to enhanced mass turnover and a more rapid glacier response to climatic forcing^45,46^. In addition, the daily model produces more negative annual mass balances (Supplementary Figs. 2 and 8). While these effects accumulate over time, the differences in modelled ice volume remain generally moderate. By the end of the century, the daily model typically projects 2-5% more ice-volume loss than the monthly model, expressed relative to the 2025 glacier volume (Fig. 3e). New Zealand also exhibits enhanced ice loss in the daily model, although this does not appear to be associated with a systematically steeper mass-balance gradient (Supplementary Fig. 3).

The projected amplified glacier loss for the daily model is even more pronounced for Svalbard, the Russian Arctic and the Antarctic and Sub-Antarctic glaciers (Fig. 3f; third pattern). In these low-relief, ice-cap-dominated regions, the narrow elevation range strongly enhances the sensitivity to surface-type transitions and melt-albedo feedbacks, which are further intensified by accelerated warming and the increasing frequency of extreme events associated with Arctic Amplification^47^. Resolving these processes at daily timescales produces markedly steeper mass-balance gradients (Fig. 3b and Supplementary Fig. 3), more negative mass balances (Supplementary Figs. 2 and 8) and substantially greater ice loss. For these regions, the daily model projects up to ~15% greater relative ice loss over 2025–2060 (vs. monthly model), reaching ~20–25% over 2025–2100 (Fig. 3f). These values should be interpreted as upper-end estimates, as these regions are particularly sensitive to calibration choices (see “Methods” and Supplementary Figs. 4 and 5). These differences are also more pronounced than for other high-latitude regions, such as Arctic Canada North, Arctic Canada South and Greenland periphery (Fig. 3e), where the slightly broader elevation ranges and weaker surface-type feedbacks limit the steepening of mass-balance gradients (for daily vs. monthly model).

Iceland, by contrast, is the only region exhibiting reversed divergence associated with precipitation-phase effects (Fig. 3g; fourth pattern). In this case, the daily model projects 4% less ice loss than the monthly model by 2100. The smaller ice loss in the daily model results from the specific precipitation regime, where heavy precipitation events frequently coincide with relatively warm conditions, as mild south-westerly air masses typically deliver both heat and moisture from the North Atlantic^48^. In contrast, colder periods dominated by northerly flow are generally drier. Resolving these co-occurrences at daily timescales results in precipitation being classified more often as rain rather than snow (Fig. 3c), reducing snow accumulation. Monthly averaging smooths over these warm events, overestimates the fraction of precipitation falling as snow, and thus overestimates accumulation. This bias propagates into calibration, yielding parameters that slightly increase glacier mass-balance sensitivity to future warming (see “Methods” and Supplementary Figs. 4 and 5).

We further tested the sensitivity of the volume projections to the calibration design by repeating the projections with an alternative calibration setup (the original, independent setup, in which C_prec_, T_bias_ and the DDFs are calibrated separately for the daily and monthly models, see “Methods”). This experiment yields similar or slightly smaller daily–monthly differences, while the regional 21st-century volume changes remain broadly consistent (Supplementary Fig. 10). This indicates that the main regional patterns described above are robust to the choice of calibration strategy.

While a direct comparison with existing global-scale glacier projections from the Climate Model Intercomparison Project version 6 (CMIP6) is complicated by to differences in the choice of the reanalysis product^49^, future forcing (i.e., a different CMIP6 global climate model (GCM) ensemble considered), model setup, and exclusion of glaciers smaller than 5 km^2^, there is generally good agreement between values reported in the literature^13,14^ and projections from our monthly-model (Supplementary Fig. 11). The slightly smaller losses projected by our monthly model compared to previous GloGEM CMIP6 results likely reflect differences in the selected GCM ensemble and the use of an extended reanalysis period up to 2024, which affects the reference state. Updated calibration choices and the >5 km² glacier subset may also contribute, but are unlikely to dominate as we capture the majority of the ice volume (see Supplementary Table 1). The Antarctic and Sub-Antarctic glaciers form a notable exception, for which both the daily and the monthly models project substantially smaller volume losses than reported in previous global assessments, especially under SSP5-8.5. Here, projected differences are particularly sensitive to the representation of surface climate in the reanalysis forcing and of frontal ablation^49–51^, highlighting that forcing and model uncertainty can outweigh temporal-resolution effects in cold, low-melt regimes. In contrast, for Svalbard and the Russian Arctic, our projections are more negative than in previous assessments and are also sensitive to calibration strategy (Supplementary Fig. 10). The magnitude of projected daily–monthly differences in these polar and subpolar regions should therefore be interpreted with care, even though the sign and overall pattern of the temporal-resolution effect remain robust.

Building on the analyses above, we quantify the impact of temporal resolution on projections of global glacier volume. Relative to 2025, our daily model projects global ice losses of 26 ± 10% (SSP1-2.6) and 47 ± 17% (SSP5-8.5) by 2100. Compared with the monthly model, this corresponds to an additional global ice loss of 4–5% by mid-century, increasing to 5–10% by 2100, depending on the scenario (Fig. 4 and Supplementary Table 3). In absolute terms, this implies that the daily model yields 7–15% less global glacier ice remaining by the end of the century compared to the monthly model. The divergence between daily and monthly projections increases steadily throughout the 21^st^ century and does not stabilise by 2100 (Fig. 4b). Given the strong trend still apparent at the end of the century, and previous studies highlighting the multi-centennial response timescales involved in global glacier evolution^19,52^, these temporal-resolution effects are likely to persist beyond the 21st century. A direct comparison with global CMIP6-based projections remains challenging due to differences in model configuration, including the choice of GCM ensembles and reanalysis data. Nevertheless, our daily model indicates slightly greater 21^st^-century volume loss than previous CMIP6-based projections^14^^,^ whereas our monthly configuration yields slightly less loss (Supplementary Table 4).

### Implications for large-scale glacier modelling and impacts

The temporal resolution of glacier models is more than a technical choice: it can shape both projected glacier change and its downstream impacts. This is particularly relevant when glacier projections are used in coupled hydrological, land-surface, and impact-assessment frameworks, where the timing, variability and extremes of glacier melt can matter as much as long-term changes in glacier volume. Daily glacier models can explicitly represent nonlinear responses associated with temperature thresholds, short-lived meteorological extremes and rapid surface-state transitions, whereas monthly models generally represent these processes in an aggregated way. Our results show that these differences can propagate into runoff seasonality, interannual variability and long-term ice-volume evolution. Temporal resolution should therefore be considered carefully in impact assessments such as studies of (dry-season) water supply^3,34^ or hydropower production in glacier-fed catchments.

Adopting daily resolution at the global scale, however, comes with substantial computational costs: in our simulations, resolving daily variability increases processing time substantially compared with monthly simulations (see “Methods”). Although continued increases in computational capacity and model development will likely alleviate these constraints, monthly models will probably remain the standard for many applications in the near term, particularly those focused on large ensembles or probabilistic assessments. The key challenge is therefore not choosing between daily and monthly models but balancing physical realism with computational feasibility according to the addressed question.

In this study, we identified several mechanisms affecting glacier projections that provide potential pathways for improving monthly models. Assumptions of stationary sub-monthly temperature variability based on reanalysis data (see “Methods”) are increasingly inconsistent with observed changes in the variance of temperature distributions under climate change^36,53–55^. Accounting for such non-stationarity would require daily GCM output or other robust information on future changes in sub-monthly temperature variability, to be readily available; however, this is not always the case for all models, scenarios or long-term projection experiments. Likewise, accumulation schemes based solely on monthly mean temperatures misrepresent snowfall where precipitation frequently coincides with warm conditions, as illustrated by the Iceland example. In an additional sensitivity experiment for Iceland, we ran the monthly GloGEM model with accumulation determined from the daily temperature evolution, while the remaining monthly model structure was left unchanged (Supplementary Fig. 12). In that case, the resulting ice-volume evolution shifts much closer to the daily model, confirming that precipitation-phase partitioning is the main driver of the reversed daily–monthly divergence in Iceland (see Fig. 3g). This suggests that relatively simple corrections for sub-monthly temperature variability and precipitation-phase partitioning could improve monthly glacier projections. Refining these components may therefore reduce some of the structural biases associated with temporal aggregation, although their robustness under changing climatic conditions remains uncertain. In parallel, the growing availability of new observational constraints, such as snowlines or snow cover datasets^56^, provides new opportunities to better constrain the spatial extent of snow cover in glacier models. This could help reduce some of the biases present in glacier models, e.g., those linked to model equifinality, where different parameter combinations can reproduce similar observations^18^. For the daily version of GloGEM, such observations are particularly promising because remotely sensed daily snow-cover patterns can be compared directly with simulated snow conditions and used as additional calibration or validation constraints^57^. By contrast, observations representing specific points in time are more difficult to incorporate consistently in a monthly model framework.

In a warming climate marked by more frequent extremes, low-temporal-resolution modelling suppresses short-term variability, which can result in a systematic underestimation of glacier loss. By resolving daily meteorological variability, our simulations consistently project more glacier mass loss than the current monthly GloGEM configuration across most regions, highlighting the potential importance of temporal resolution for downstream impacts. By 2100, our daily model projects a global glacier contribution of 86–157 mm to global mean sea-level rise, exceeding monthly estimates by 20–33 mm (Fig. 4a, b). This additional contribution in the daily model is substantial, although it remains within the GCM ensemble spread shown in Fig. 4a. These additional centimeters resulting from the transition to daily modelling could have direct implications for exposure to coastal flooding, erosion and saltwater intrusion^58^. As such, our findings indicate that temporal resolution is not a technical detail but a modelling choice with direct consequences for impact assessments. Future glacier projection efforts should therefore explicitly consider temporal resolution, which, combined with informed choices regarding bias correction of climate data^59^, climate forcing^6,49^ and calibration strategies^60–62^, will help produce robust and policy-relevant projections of glacier change in a warming world.

## Methods

### Geometric data

We simulate all glaciers from the Randolph Glacier Inventory v6.0^44^ larger than 5 km^2^, comprising 12,571 glaciers, or about 6% of the global glacier number. These glaciers account for 148,075 km^3^ of ice (94% of the global glacier ice volume) and 583,189 km^2^ of glacierised area (83% of the global glacier area) (Supplementary Table 1). Although this threshold excludes a large number of small glaciers, especially in regions with many small ice bodies, such as in High Mountain Asia, our aim is not to simulate every individual glacier but to isolate the effect of daily versus monthly forcing within a consistent global modelling framework.

Ice thickness distributions are taken from Farinotti et al. (2019)^63^, who combined up to five inversion models^64^ to produce a consensus global estimate representative of early twenty-first-century conditions. Glacier surface topography is derived from the Shuttle Radar Topography Mission (SRTM) digital elevation model (DEM)^65^ for glaciers located between 60°S and 60°N. For higher-latitude regions, surface elevation is obtained from a combination of ASTER, ArcticDEM and REMA datasets.

### Climate data

Both the daily and monthly configurations of GloGEM are driven by gridded near-surface air temperature and precipitation extracted from the nearest land grid cell to each glacier centroid. For the historical period (1980–2024), we use ERA5-Land reanalysis data at 0.1° (~9 km) spatial resolution^66^. ERA5-Land is generated by rerunning the land component of ERA5^67^, providing globally consistent, high-resolution meteorological fields and extending coverage to recent years, including major climate extremes, such as the exceptional melt season of 2022 in the European Alps^25,40^. Because ERA5-Land provides valid values only over land surfaces, coastal glaciers whose centroid falls offshore are assigned the nearest valid land grid cell. We do not expect this procedure to introduce a systematic bias, although local representativeness errors may occur in coastal or steep terrain (see section Mass balance model).

For the 2025–2100 period, climate forcing is taken from five global climate models (GCMs) from the Coupled Model Intercomparison Project Phase 6 (CMIP6)^68^, selected within the Inter-Sectoral Impact Model Intercomparison Project Phase 3b (ISIMIP3b) framework^69^: GFDL-ESM4, UKESM1-0-LL, MPI-ESM1-2-HR, MRI-ESM2-0 and IPSL-CM6A-LR. These models were chosen to represent a broad range of climate sensitivities, precipitation responses and regional climate characteristics, while ensuring continuous availability of daily and monthly variables required for glacier modelling across all considered Shared Socioeconomic Pathways (SSPs). Together, they span much of the CMIP6 uncertainty range relevant for cryospheric impact assessments and are commonly used in coordinated impact modelling efforts.

Simulations are performed under three SSPs (SSP1-2.6, SSP3-7.0 and SSP5-8.5), representing low-, intermediate- and high-emission scenarios consistent with the IPCC Sixth Assessment Report^70^. To ensure consistency between historical and future forcing, GCM outputs are bias-corrected over 1991–2020 using the delta method. This approach corrects monthly mean biases but does not explicitly adjust daily variability statistics, such as variance, autocorrelation or extreme values. Applying a more advanced daily bias-correction method only to the daily forcing would introduce an additional methodological difference and could therefore confound the comparison between model configurations.

### Mass balance observations

Model calibration (see Calibration of the mass balance model) is based on geodetic glacier mass balances for the period 2000–2019^39^. These estimates are derived from glacier surface elevation changes obtained from ASTER stereo imagery^71^ and converted to mass using an ice density of 850 kg m^−3^^72^. The dataset provides glacier-specific estimates of mean mass change over a 20-year period for nearly all glaciers worldwide and forms a consistent global reference for large-scale model calibration^13,14^.

For independent validation, we use glacier-wide and elevation-band annual climatic mass balance observations from the World Glacier Monitoring Service (WGMS)^37^. We select glaciers with at least three years of observations between 1980 and 2024 (175 glaciers in total). Earlier measurements are excluded because the glacier model assumes fixed glacier geometry prior to the inventory date (approximately the year 2000), such that modelled geometries may not be representative of earlier glacier states. The validation is performed using the cross-calibrated daily and monthly GloGEM configurations that are also used for the projections (see section Calibration consistency across temporal resolutions). The WGMS dataset includes both point-based measurements (stake and snow-pit observations) and glacier-wide balances, allowing for robust validation across most RGI regions, although observations are spatially unevenly distributed and biased towards smaller, mid-latitude glaciers. As an additional validation, we compare regional mass changes with independent regional estimates from GLAMBIE^1^.

### Glacier modelling

Glacier evolution is simulated using GloGEM^16^, an elevation-band model designed to compute glacier mass balance, geometry and volume evolution at regional to global scales. GloGEM has been widely applied to large-scale projections of glacier change^73–75^, glacier runoff^4,5,24,33,76^, and assessments of the impacts of glacier loss on ecosystems and societies^11,77,78^. In GloGEM, glacier geometries are discretised into elevation bands of 10 m, and individual glacier tributaries are merged into single glacier entities to ensure consistent treatment of mass balance and geometry evolution across complex glacier systems^16^.

### Mass balance model

The core of GloGEM is a surface mass-balance model that computes accumulation, ablation, and refreezing independently for each elevation band. Accumulation is determined from solid precipitation, with the phase of precipitation inferred from near-surface air temperature using a linear snow–rain transition between 0.5 and 2.5 °C. While recent work has shown that snow-rain transition temperatures can vary regionally depending on local climatic conditions (e.g., Jennings et al., 2025^26^), a fixed transition range is applied here globally for consistency across all glacierised regions.

The standard GloGEM configuration operates at monthly resolution and is therefore referred to here as the “monthly model” or “monthly configuration”^16^. Despite its monthly forcing, this configuration includes a pseudo-daily statistical representation of temperature variability to approximate daily-scale melt processes. Sub-monthly temperature variability is parameterised using a monthly standard deviation of daily temperatures derived from historical data, allowing reconstruction of a within-month temperature distribution. This enables positive degree-day sums to occur even when monthly mean temperatures remain below freezing. In the daily configuration, melt is computed from daily mean air temperatures, which are used directly to derive positive degree-day sums. Accumulation is calculated from daily total precipitation and daily mean air temperature, thereby explicitly resolving day-to-day variability in precipitation phase. In contrast, the monthly configuration estimates accumulation from monthly total precipitation and mean monthly air temperature, without explicitly resolving day-to-day variability in precipitation phase.

In both configurations, precipitation is vertically adjusted using a regional precipitation lapse rate (*dP/dz*) ranging from 0.015 to 0.025% m^−1^. To account for moisture depletion and wind redistribution at high elevations, an exponential decay is applied over the uppermost 25% of the elevation range for glaciers exceeding 1000 m in elevation range. To compensate for systematic biases in gridded climate data and unresolved processes such as avalanching, a glacier-specific precipitation correction factor (*C*_*prec*_) is applied and calibrated for each glacier (see Calibration of the mass balance model). Precipitation at elevation band z_i_ is thus given by:

1 $${{{{\rm{P}}}}}_{{{{\rm{i}}}}}={{{{\rm{P}}}}}_{{{{\rm{cell}}}}}*{{{{\rm{C}}}}}_{{{{\rm{prec}}}}}*(1+\left(\left({{{{\rm{z}}}}}_{{{{\rm{i}}}}}-{{{{\rm{z}}}}}_{{{{\rm{ref}}}}}\right)*\frac{{{{\rm{dP}}}}}{{{{\rm{dz}}}}}\right))$$

where *P*_*i*_ is precipitation at elevation band *z*_*i*_*, P*_*cell*_ is the gridded precipitation at reference elevation *z*_*ref*_, and *dP/dz* is the imposed vertical precipitation gradient.

Ablation is computed using a temperature-index (degree-day) approach, applying distinct degree-day factors (DDFs) for snow, firn, and ice, with fixed ratios of 1:1.5:2, respectively, based on Hock (2003)^79^ and Huss and Hock (2015)^16^. In the daily configuration, surface type (and thus the applied DDFs) is updated at a daily time step, whereas in the monthly configuration, it is updated once per month.

Air temperature at each elevation band is calculated as:

2 $${{{{\rm{T}}}}}_{{{{\rm{i}}}}}={{{{\rm{T}}}}}_{{{{\rm{cell}}}}}+\left({{{{\rm{z}}}}}_{{{{\rm{i}}}}}-{{{{\rm{z}}}}}_{{{{\rm{ref}}}}}\right)*\left(\frac{{{{\rm{dT}}}}}{{{{\rm{dz}}}}}\right)+{{{{\rm{T}}}}}_{{{{\rm{bias}}}}}$$

where *T*_*i*_ is air temperature at elevation band *z*_*i*_*, T*_*cell*_ is the gridded near-surface air temperature, *dT/dz* is the temperature lapse rate, and *T*_*bias*_ is a calibrated temperature offset (see Calibration of the mass balance model). Monthly lapse rates are derived from temperature gradients between multiple atmospheric layers in the ERA5 dataset over the historical period, allowing seasonal variability in vertical temperature structure to be represented. Refreezing is simulated using a one-dimensional heat-conduction scheme with 10 vertical layers applied to the upper 10 m of the snowpack^16^. For marine-terminating glaciers, frontal ablation is parameterised following a modified formulation^80^, scaled by terminus elevation, water depth and glacier width. Processes such as basal melt, debris cover and internal accumulation are not included in the model setup to ensure methodological consistency and comparability between daily and monthly temporal resolutions. While some of these processes, particularly debris-cover effects, may be regionally important (e.g., in High Mountain Asia) (Herreid and Pellicciotti, 2020)^81^, both model configurations are calibrated against geodetic mass-balance observations^39^, which inherently integrate their net effects. As a result, these processes are indirectly reflected in the calibrated parameter values, and their omission is not expected to systematically affect the comparison between daily and monthly model configurations.

### Calibration of the mass balance model

Model calibration ensures that the modelled glacier-wide mass balance reproduces the glacier-specific geodetic mass-balance estimates of Hugonnet et al. (2021)^39^ for 2000–2019 to within ca. ±0.1 m w.e. yr^−1^. To achieve this, three parameters are calibrated individually for each glacier: the precipitation correction factor (*C*_*prec*_), the degree-day factor for snow (*DDF*_*snow*_), and a temperature bias (*T*_*bias*_). These parameters adjust coarse-resolution climate inputs to the glacier scale and account for unresolved processes such as avalanching, debris cover, and albedo variations.

During calibration, the model is run over the period 2000–2019 using an initial parameter set, after which parameters are adjusted sequentially in three steps:

In the first calibration step, the precipitation correction factor (*C*_*prec*_) is tuned within the range 0.8–2.5 to account for biases in precipitation forcing. This parameter is prioritised, as precipitation (and thus accumulation) is typically one of the most uncertain climate variables in mountainous regions. During this step, the degree-day factors are kept fixed at their standard values.

If no satisfactory solution is found within the prescribed precipitation-correction range, *DDF*_*snow*_ is adjusted within the range 1.75–4.5 mm d^−1^ °C^−1^. The degree-day factors for firn and ice are defined relative to those of snow, scaled by factors of 1.5 and 2, respectively, and therefore vary proportionally during this calibration step.

If still no satisfactory match is found after adjusting both *C*_*prec*_ and *DDF*_*snow*_ within their prescribed limits, a temperature bias (*T*_*bias*_) is applied to correct for systematic temperature offsets in the climate input. This parameter has no predefined bounds.

The calibration procedure is repeated after an initial calibration cycle^14^. In this second iteration, grid-cell-mean temperature biases, derived from the first calibration across all glaciers within each grid cell, are imposed prior to recalibration. This ensures that temperature biases remain more consistent among glaciers within the same grid cell and prevents unrealistically large differences in temperature bias between individual glaciers. By constraining these biases, the approach reduces persistent strong positive or negative temperature offsets in the climate input and avoids the precipitation correction factor drifting towards its bounds. Glacier geometry is held constant at its initial state during the calibration period (2000–2019).

### Calibration consistency across temporal resolutions

Independent calibration of the daily and monthly configurations of GloGEM can introduce small but systematic differences in climatic corrections (precipitation correction and temperature bias), which may obscure the interpretation of temporal-resolution effects. To isolate the impact of temporal resolution itself, we apply a cross-calibration procedure.

First, the daily and monthly model configurations are calibrated independently using the full three-step calibration procedure described above, yielding glacier-specific values of the precipitation correction factor (*C*_*prec*_), temperature bias (*T*_*bias*_), and degree-day factors (DDFs). For most glaciers and regions, the calibrated parameters obtained for the daily and monthly configurations are broadly similar (Supplementary Table 5, Supplementary Figs. 4 and 5). In some regions, the calibrated values for both the daily and the monthly versions approach the prescribed parameter bounds. For example, in the Caucasus and Middle East, many glaciers reach the upper limit of *C*_*prec*_ (2.5), whereas in several other regions *C*_*prec*_ values cluster near the lower limit (0.8), indicating that precipitation from the underlying ERA5-Land grid is corrected downward. The fact that these bounded values occur in both the daily and monthly versions indicates that there is no systematic difference between the two temporal resolutions. Supplementary Fig. 4 further shows that differences in T_bias_ between the daily and monthly calibrations are generally small compared to the regional spread in calibrated values.

In the cross-calibration approach, the mean values of *C*_*prec*_ and *T*_*bias*_ from the daily and monthly calibrations are then computed and imposed identically in both model configurations. With *C*_*prec*_ and *T*_*bias*_ fixed, the degree-day factors are subsequently recalibrated separately for the daily and monthly configurations to ensure consistency with observed geodetic mass balance. This step is required because the integration of positive degree days differs across temporal resolutions, such that identical DDF values do not necessarily produce equivalent melt under the same climatic forcing.

This approach enforces a common climatic baseline across model configurations while allowing melt sensitivities to adjust to temporal resolution, ensuring that remaining differences in model behaviour can be attributed primarily to temporal-resolution effects. Following the averaging of *C*_*prec*_ and *T*_*bias*_, the recalibrated DDF values are generally slightly higher in the daily model than in the monthly model (Supplementary Fig. 5). The largest differences occur in Svalbard, the Russian Arctic, and the Subantarctic and Antarctic Islands. In these regions, the independently calibrated daily model generally has lower *C*_*prec*_ and higher *T*_*bias*_ values than the monthly model. After averaging these parameters, the daily model therefore tends to produce relatively more accumulation and/or insufficient melt compared to what would be needed to reproduce the observed geodetic mass balance.

This cross-calibration design involves a trade-off. The averaged *C*_*prec*_ and *T*_*bias*_ values do not necessarily represent the optimal parameter combination for either the daily or the monthly model when considered independently. Instead, they enforce a common climate forcing between temporal resolutions, at the cost of moving both models slightly away from their independently calibrated optima. We use this setup to isolate temporal-resolution effects, rather than to identify a unique best-fit parameter set for each model configuration.

### Glacier evolution

In both the daily and monthly models, glacier geometry (area and ice thickness) is updated annually based on the mean specific mass balance using the dh-parameterisation^82^, ensuring consistent long-term glacier evolution across temporal resolutions. The simulations were performed on a Linux workstation equipped with two Intel Xeon E5-2687W v4 processors, providing 24 physical CPU cores in total. The model runs used here were executed as single-core jobs, so each individual simulation used one CPU core rather than the full multi-core capacity of the machine. Under this setup, the daily version of the model takes approximately ten times longer than the monthly version. For a test sample of about 1000 glaciers, this corresponds to roughly 1.2 h of computation time for the daily model.

### Glacier runoff modelling

Glacier runoff is defined as the total water flux originating from areas that are glacierised at the time of simulation, comprising meltwater from snow, firn and ice as well as rainfall over the glacier surface, minus refreezing^4^. Evaporation is assumed to be negligible. As glaciers retreat over time, runoff calculations continue to include precipitation and melt contributions from the progressively deglaciating terrain within the initial RGI v6.0 glacier outlines. In the daily configuration, glacier runoff is computed at a daily time step, allowing short-term meteorological variability in melt and rainfall events to be explicitly resolved. In the monthly (standard) configuration, glacier runoff is computed as the total runoff volume originating from the glacier outline over an entire month. In both model configurations, all surface water is assumed to run off instantaneously and reach the glacier terminus on the same day, as GloGEM does not include a runoff routing scheme. For large glaciers, particularly when runoff is resolved at daily resolution, this assumption may not hold, as runoff can take several days to travel through the glacier system towards the glacier snout.

## Supplementary information

Supplementary Information

Transparent Peer Review file

## Data Availability

All the datasets needed for the glacier simulations are documented on https://doi.org/10.5281/zenodo.21792181^83^.

## Code availability

As part of this study, we have made a fully documented version of GloGEM publicly available on GitHub [https://github.com/eth-vaw-glaciology/GloGEM]^84^. This release supports both daily and monthly input configurations and includes online documentation describing the model setup, required input data, and simulation workflow. Simulation outputs associated with this study are made available in NetCDF format through https://doi.org/10.5281/zenodo.21792181^84^.
