# Supplementary material for: Resolving short-term meteorological variability amplifies projected glacier loss and sea-level rise
Source: Nat Commun. 2026 Oct 19;17:10696. doi: 10.1038/s41467-026-78260-y (PMC13680477; doi:10.1038/s41467-026-78260-y)
Supplement: Supplementary file 1 [file 41467_2026_78260_MOESM1_ESM.pdf]

Extended Data

Extended Data Table 1: Overview of modelled glaciers by region. Number of modelled glaciers, glacierised area and total glacier volume included in the simulations for each region. Percentages indicate the relative contribution of the regional total.

Region	Modelled glaciers		Glacier volume (km^3)		Glacier area (km^2)	
**Alaska**	1296	18 057	95%	5%	71 192	82%
**W. Canada and USA**	468	718	68%	2%	6 045	41%
**Arctic Canada N.**	1402	26 235	93%	31%	99 459	95%
**Arctic Canada S.**	1094	7 789	90%	15%	34 937	85%
**Greenland P.**	2385	13 258	99%	12%	76 937	86%
**Iceland**	99	3 746	99%	17%	10 646	96%
**Svalbard and J.M.**	512	7 324	98%	32%	32 245	95%
**Scandinavia**	137	212	71%	4%	1 330	45%
**Russian Arctic**	606	14 604	99%	57%	50 440	98%
**North Asia**	73	74	53%	1%	742	31%
**Central Europe**	123	81	62%	3%	904	43%
**Caucasus and M.E.**	53	37	62%	3%	489	37%
**Central Asia**	1465	2 282	70%	3%	22 984	47%
**South Asia W.**	911	2 340	82%	3%	20 111	60%
**South Asia E.**	532	595	68%	4%	7 085	48%
**Low Latitudes**	89	38	38%	3%	637	27%
**Southern Andes**	622	5 062	95%	4%	21 916	75%
**New Zealand**	30	50	71%	1%	441	38%
**Subant. and A.I.**	656	47 925	99%	24%	128 580	97%
**Global**	12 571	148 075	94%	6%	583 189	83%

Extended Data Table 2: Evaluation of modelled mass balances against WGMS observations. Regional evaluation of modelled mean specific mass balances compared with data from the WGMS[37]. Only glaciers with at least three years of observations are included. Regions for which this condition is only met for one or less glaciers are shaded in light orange. RMSE Da. and RMSE Mo. denote the root mean square error, MAE Da. and MAE Mo. the mean absolute error, and R^2 Da. and R^2 Mo. the coefficient of determination for the cross-calibrated daily and monthly model configurations, respectively.

Region	RMSE Da.	RMSE Mo.	MAE Da.	MAE Mo.	R^2 DA.	R^2 MO.	Nr. Glaciers	Nr. Years	Nr. Observations
Alaska	0.59	0.56	0.48	0.49	0.93	0.93	8	45	207
W. Canada and USA	0.48	0.47	0.38	0.39	0.99	0.98	21	45	410
Arctic Canada N.	0.17	0.24	0.16	0.21	0.93	0.95	3	45	132
Arctic Canada S.							0	0	0
Greenland P.	0.60	0.62	0.48	0.51	0.69	0.65	6	40	74
Iceland	0.54	0.70	0.44	0.59	0.95	0.92	11	38	302
Svalbard and J.M.	0.21	0.53	0.18	0.42	0.98	0.96	14	45	270
Scandinavia	0.45	0.56	0.39	0.47	0.98	0.99	25	45	682
Russian Arctic	0.44	0.41	0.44	0.41	0.88	0.89	1	5	5
North Asia	0.50	0.49	0.31	0.33	0.24	0.27	4	39	91
Central Europe	0.46	0.70	0.37	0.55	0.99	0.98	35	45	1009
Caucasus and M.E.	0.21	0.22	0.18	0.21	0.96	0.97	2	45	86
Central Asia	0.16	0.20	0.14	0.17	0.99	0.96	16	45	284
South Asia W.	0.52	0.54	0.36	0.40	0.59	0.56	6	34	58
South Asia E.	0.36	0.32	0.28	0.29	0.79	0.78	10	38	102
Low Latitudes	1.73	1.76	1.29	1.29	0.78	0.77	4	34	67
Southern Andes	0.39	0.46	0.31	0.34	0.53	0.37	4	44	56
New Zealand	0.70	0.62	0.53	0.47	0.86	0.72	1	20	20
Subant. and A.I.	0.50	0.69	0.45	0.58	0.90	0.73	4	26	77

Extended Data Table 3: Modelled regional and global ice volume change by 2100 relative to 2025. Values show projected glacier ice-volume change (%) relative to 2025. Reported values represent the multi-model mean across the five GCMs used, with ±1 standard deviation indicating the ensemble spread.

Region	Daily model			Monthly model		
	SSP1-2.6	SSP3-7.0	SSP5-8.5	SSP1-2.6	SSP3-7.0	SSP5-8.5
Alaska	44 ± 11	61 ± 15	66 ± 17	40 ± 11	58 ± 15	63 ± 19
W. Canada and USA	69 ± 15	90 ± 9	92 ± 8	67 ± 14	87 ± 10	90 ± 9
Arctic Canada N.	11 ± 5	20 ± 13	27 ± 17	12 ± 4	20 ± 11	25 ± 15
Arctic Canada S.	37 ± 14	52 ± 18	58 ± 21	35 ± 12	48 ± 17	55 ± 20
Greenland P.	24 ± 12	42 ± 16	50 ± 20	24 ± 10	39 ± 13	45 ± 17
Iceland	24 ± 17	40 ± 21	45 ± 23	28 ± 15	44 ± 19	48 ± 21
Svalbard and J.M.	48 ± 27	66 ± 21	71 ± 22	30 ± 21	44 ± 23	49 ± 26
Scandinavia	40 ± 21	67 ± 16	72 ± 18	40 ± 19	64 ± 16	70 ± 19
Russian Arctic	50 ± 22	65 ± 22	70 ± 22	26 ± 18	37 ± 22	43 ± 25
North Asia	61 ± 15	80 ± 10	86 ± 7	59 ± 14	77 ± 12	82 ± 10
Central Europe	78 ± 14	95 ± 4	98 ± 2	76 ± 14	94 ± 5	97 ± 3
Caucasus	66 ± 11	92 ± 4	94 ± 4	65 ± 10	92 ± 4	94 ± 4
Central Asia	44 ± 16	74 ± 13	82 ± 13	43 ± 14	70 ± 13	78 ± 13
South Asia W.	31 ± 13	58 ± 14	69 ± 13	32 ± 11	57 ± 13	68 ± 13
South Asia E.	72 ± 10	91 ± 5	94 ± 3	73 ± 8	91 ± 5	94 ± 3
Low Latitudes	73 ± 11	97 ± 1	98 ± 1	73 ± 11	98 ± 1	98 ± 1
Southern Andes	50 ± 8	73 ± 11	79 ± 10	40 ± 10	66 ± 14	73 ± 14
New Zealand	51 ± 5	88 ± 5	89 ± 7	40 ± 5	81 ± 8	83 ± 11
Subant. and A.I.	13 ± 4	27 ± 14	33 ± 14	8 ± 1	14 ± 5	16 ± 6
Global	-26 ± 10	-42 ± 15	-47 ± 17	-21 ± 8	-33 ± 12	-37 ± 14

Extended Data Table 4: Global glacier volume by 2100 relative to 2015 and comparison to GloGEM CMIP6 global glacier evolution projections from Zekollari et al. (2024). To allow for a comparison to Zekollari et al. (2024), reported values are the ensemble medians, with the 95% confidence intervals indicating the multi-climate model spread across simulations.

Region	Daily model			Monthly model		
	**SSP1-2.6**	**SSP3-7.0**	**SSP5-8.5**	**SSP1-2.6**	**SSP3-7.0**	**SSP5-8.5**
**This study**	-27 ± 18	-43 ± 28	-48 ± 31	-23 ± 15	-34 ± 23	-39 ± 27
**Zekollari 2024 (only GloGEM)**				-25 ± 15	-39 ± 21	-46 ± 26

Extended Data Table 5: Regional calibration-parameter statistics. Mean and standard deviation of all calibration parameters for each region. The upper part of the table reports parameters from the original calibration strategy, in which the precipitation correction factor (C_{prec}), temperature bias (T_{bias}), and degree-day factors of snow (DDF_{snow}) are calibrated independently for the daily and monthly model configurations. The lower part reports the cross-calibrated parameters obtained after averaging C_{prec} and T_{bias} between the daily and monthly configurations for each glacier.

	C_{prec} (-)				T_{bias} (°C)				DDF_{snow} (mm day^{-1} °C^{-1})			
**Original calibration**	**Daily**		**Monthly**		**Daily**		**Monthly**		**Daily**		**Monthly**	
	Mean	Std	Mean	Std	Mean	Std	Mean	Std	**Mean**	**Std**	**Mean**	**Std**
**Alaska**	1.05	0.43	1.08	0.45	0.26	1.01	0.20	0.93	3.44	0.61	3.36	0.58
**W. Canada and USA**	1.21	0.43	1.29	0.49	0.00	0.02	0.00	0.00	3.08	0.24	3.04	0.19
**Arctic Canada N.**	1.01	0.47	1.02	0.48	0.55	0.86	0.44	0.83	3.77	0.71	3.72	0.72
**Arctic Canada S.**	0.93	0.35	0.97	0.40	0.60	0.82	0.39	0.65	3.87	0.65	3.78	0.67
**Greenland P.**	1.05	0.54	1.07	0.56	1.86	2.17	1.50	2.01	3.85	0.84	3.80	0.85
**Iceland**	0.84	0.13	0.84	0.13	0.29	0.43	0.33	0.46	3.99	0.56	4.02	0.53
**Svalbard and J.M.**	0.97	0.45	1.22	0.60	0.51	1.31	-0.19	1.40	3.96	0.76	3.25	0.70
**Scandinavia**	1.16	0.27	1.22	0.28	0.00	0.00	0.00	0.02	3.01	0.05	3.00	0.02
**Russian Arctic**	0.87	0.24	1.43	0.66	0.62	0.62	-0.05	0.41	4.05	0.66	3.10	0.66
**North Asia**	1.49	0.53	1.58	0.53	0.03	0.15	-0.01	0.13	3.09	0.41	3.03	0.28
**Central Europe**	0.97	0.27	1.00	0.29	0.06	0.33	0.05	0.30	3.28	0.44	3.23	0.41
**Caucasus and M.E.**	1.79	0.68	1.79	0.68	-0.02	0.49	-0.03	0.48	3.11	0.58	3.10	0.55
**Central Asia**	0.95	0.35	0.96	0.35	0.88	1.30	0.73	1.21	3.75	0.69	3.71	0.69
**South Asia W.**	0.90	0.30	0.90	0.30	2.05	2.09	1.90	2.13	3.97	0.63	3.92	0.65
**South Asia E.**	0.96	0.38	0.95	0.36	1.02	1.25	1.01	1.24	3.82	0.70	3.83	0.69
**Low Latitudes**	1.00	0.49	1.01	0.50	0.56	0.97	0.61	1.00	3.87	0.80	3.87	0.81
**Southern Andes**	0.90	0.25	0.90	0.25	0.99	1.15	0.97	1.15	3.90	0.65	3.90	0.65
**New Zealand**	1.67	0.60	1.48	0.53	0.00	0.00	0.00	0.00	2.94	0.19	2.99	0.12
**Subant. and A.I.**	0.91	0.35	1.01	0.48	2.70	3.91	1.93	3.82	4.12	0.71	3.91	0.80
**Cross-calibration**	**Daily**		**Monthly**		**Daily**		**Monthly**		**Daily**		**Monthly**	
	Mean	Std	Mean	Std	Mean	Std	Mean	Std	**Mean**	**Std**	**Mean**	**Std**
**Alaska**	1.06	0.44	1.06	0.44	0.23	0.97	1.06	0.44	3.52	0.68	3.32	0.55
**W. Canada and USA**	1.25	0.46	1.25	0.46	0.00	0.01	1.25	0.46	3.16	0.23	3.01	0.21
**Arctic Canada N.**	1.01	0.46	1.01	0.46	0.49	0.82	1.01	0.46	4.01	1.08	3.72	0.76
**Arctic Canada S.**	0.95	0.37	0.95	0.37	0.50	0.73	0.95	0.37	4.14	0.88	3.67	0.63
**Greenland P.**	1.06	0.54	1.06	0.54	1.68	2.08	1.06	0.54	4.39	1.68	3.70	0.86
**Iceland**	0.84	0.13	0.84	0.13	0.31	0.44	0.84	0.13	3.98	0.57	4.06	0.58
**Svalbard and J.M.**	1.09	0.49	1.09	0.49	0.16	1.29	1.09	0.49	5.55	1.57	2.72	0.75
**Scandinavia**	1.19	0.28	1.19	0.28	0.00	0.00	1.19	0.28	3.06	0.07	2.99	0.04
**Russian Arctic**	1.15	0.40	1.15	0.40	0.28	0.42	1.15	0.40	7.27	2.04	2.33	0.80
**North Asia**	1.53	0.51	1.53	0.51	0.01	0.12	1.53	0.51	3.24	0.94	3.00	0.30
**Central Europe**	0.97	0.27	0.97	0.27	0.06	0.33	0.97	0.27	3.28	0.45	3.21	0.41
**Caucasus and M.E.**	1.79	0.68	1.79	0.68	-0.03	0.49	1.79	0.68	3.12	0.59	3.10	0.57
**Central Asia**	0.96	0.35	0.96	0.35	0.80	1.24	0.96	0.35	4.08	1.22	3.70	0.69
**South Asia W.**	0.90	0.30	0.90	0.30	1.97	2.10	0.90	0.30	4.22	0.97	3.90	0.66
**South Asia E.**	0.95	0.37	0.95	0.37	1.01	1.24	0.95	0.37	3.84	0.73	3.84	0.70
**Low Latitudes**	1.01	0.50	1.01	0.50	0.58	0.98	1.01	0.50	3.86	0.78	4.02	0.94
**Southern Andes**	0.90	0.25	0.90	0.25	0.98	1.15	0.90	0.25	3.96	0.75	3.91	0.66
**New Zealand**	1.57	0.56	1.57	0.56	0.00	0.00	1.57	0.56	2.81	0.18	3.14	0.15
**Subant. and A.I.**	0.96	0.39	0.96	0.39	2.32	3.82	0.96	0.39	10.71	12.56	3.36	1.25

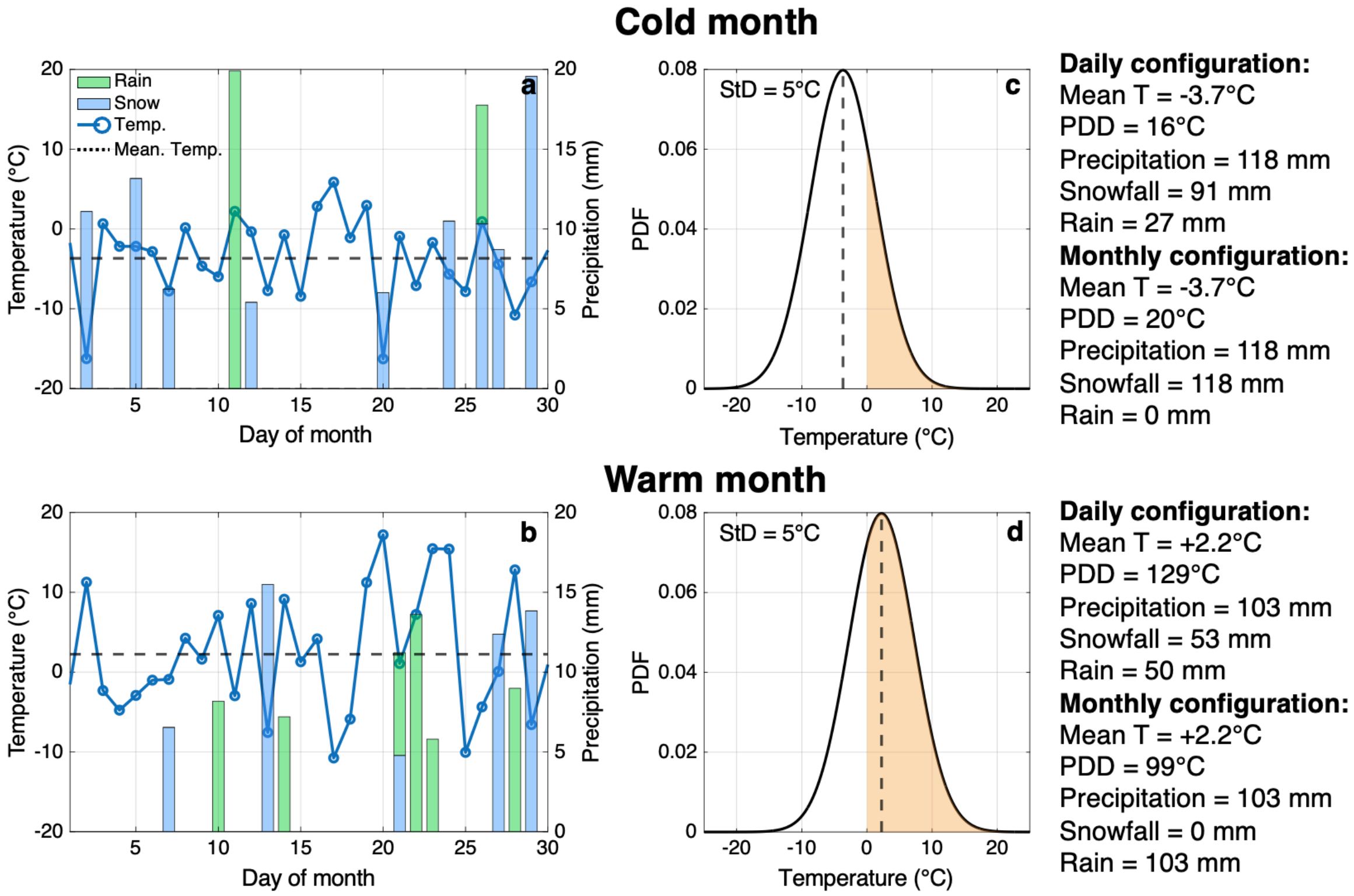

Extended Data Fig. 1: Differences between daily and monthly model configurations. a, b Daily temperature variability and the partitioning of precipitation into snowfall and rain over a representative cold and warm month, together with the corresponding monthly mean temperature. **c, d** Probability density functions (PDFs) depict the assumed temperature distribution used in the monthly model, with an illustrative standard deviation of 5°C and means derived from the respective cold and warm months. Despite a monthly mean temperature below 0°C, non-zero PDDs arise in the monthly model due to sub-monthly temperature variability. In the monthly configuration, however, snowfall depends solely on the monthly mean temperature, leading to zero snowfall in the warm month where the mean temperature exceeds 0 °C.

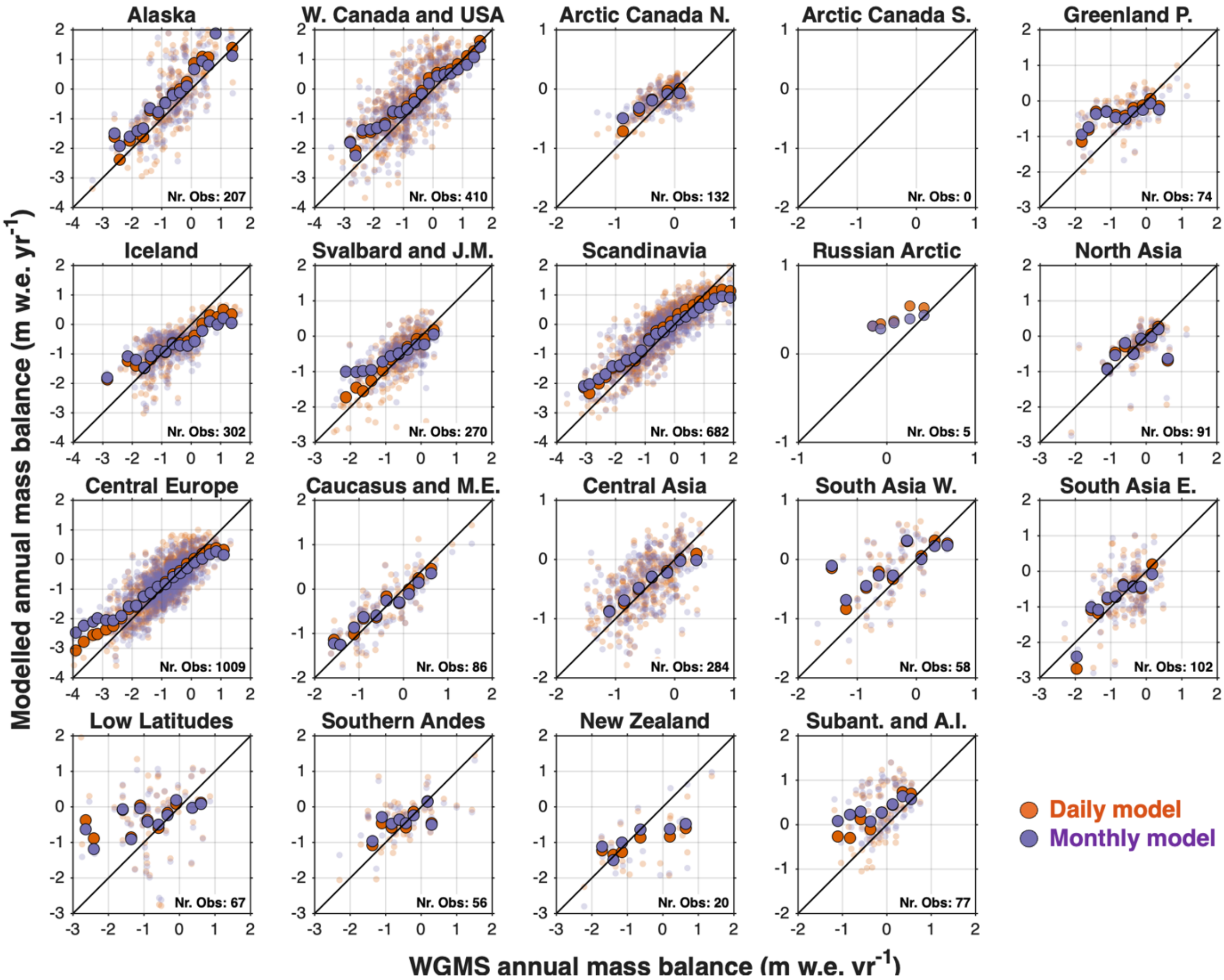

Extended Data Fig. 2: Comparison of modelled and observed glacier mass balance for the daily and monthly models. Modelled versus observed glacier-wide annual specific mass balance according to WGMS (2025) for the period 1980–2024, shown separately for each RGI region. Only glaciers with at least three years of observations are included. Large circles indicate mean values within 0.5 m w.e. bins, which helps highlight the underlying signal where sufficient data are available. For the Russian Arctic, where the number of points is too small for meaningful binning, larger circles instead show the available individual comparisons. The number of model–observation comparisons (Nr. Obs) is indicated at the bottom right of each panel.

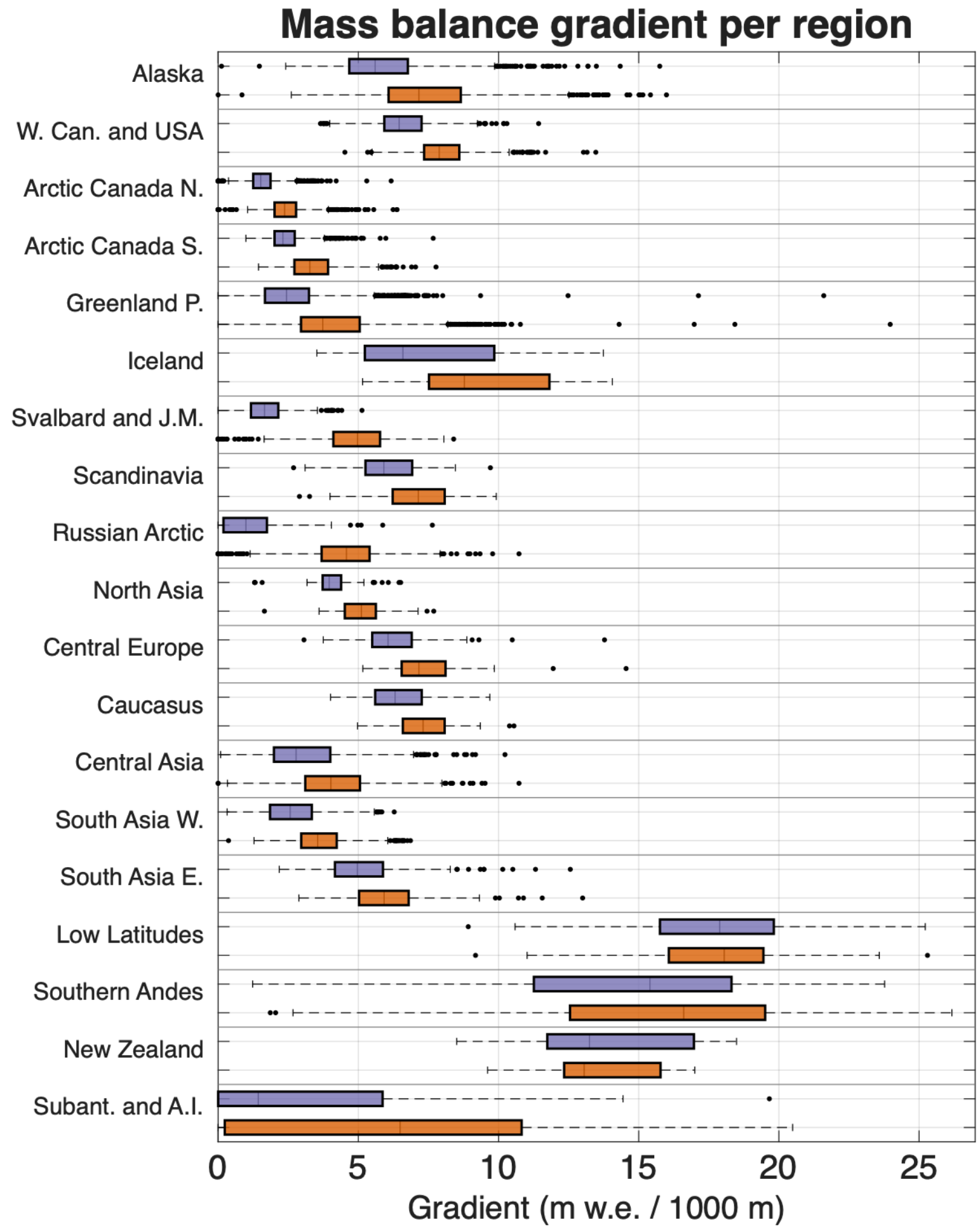

Extended Data Fig. 3: Regional differences in mass-balance gradients between the daily and monthly model. Upper panel shows mass-balance gradients in the ablation area averaged over 2000–2019 for each RGI region as simulated by the daily and monthly model.

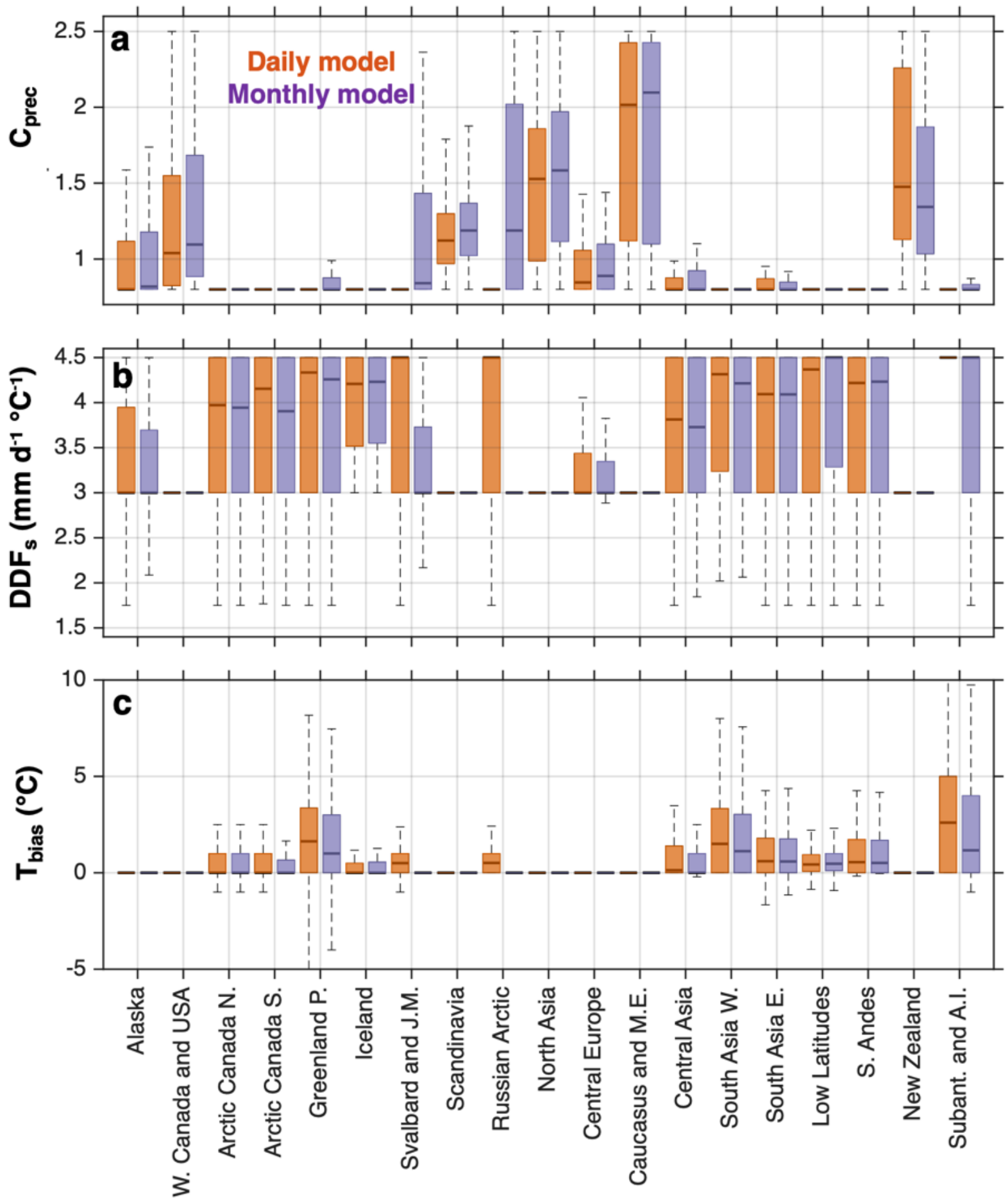

Extended Data Fig. 4: Regional distributions of calibrated model parameters for the daily and monthly model under the original calibration approach. a-c Distributions of the calibrated precipitation correction factor (C_{prec}), snow degree-day factor (DDF_s), and temperature bias (T_{bias}) obtained from the original calibration procedure. Daily and monthly model calibrations are shown in orange and purple, respectively. Boxplots summarise the distribution of glacier-specific calibration parameters within each RGI region.

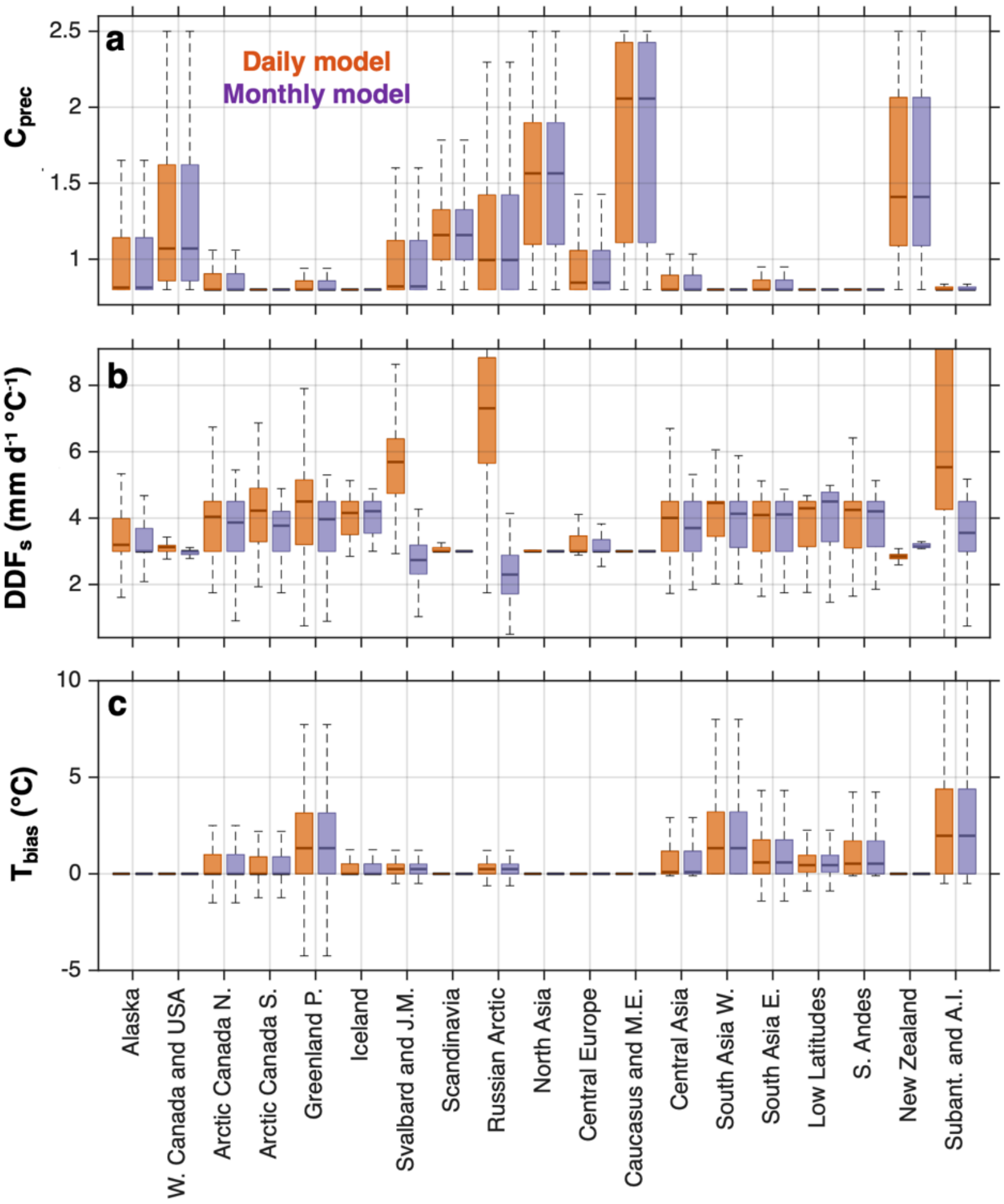

Extended Data Fig. 5: Regional distributions of calibrated model parameters for the daily and monthly model with the cross-calibration approach. a-c Distributions of the calibrated precipitation correction factor (C_{prec}), snow degree-day factor (DDFs), and temperature bias (T_{bias}) obtained from the cross-calibration procedure. Daily and monthly model calibrations are shown in orange and purple, respectively. Boxplots summarise the distribution of glacier-specific calibration parameters within each RGI region.

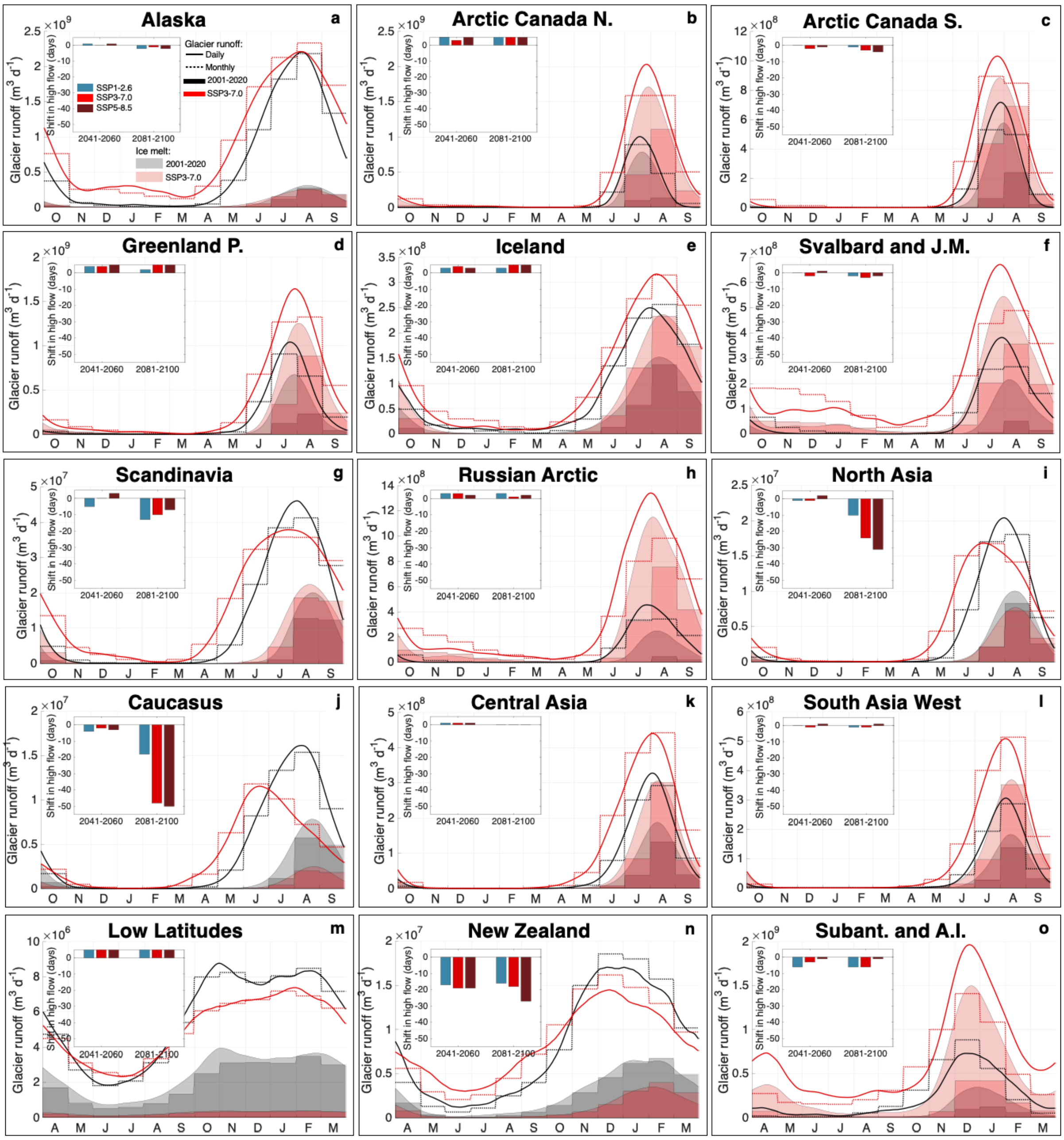

Extended Data Fig. 6: Evolution and variability of glacier runoff. Modelled mean glacier runoff at daily (solid lines) and monthly (dashed lines) temporal resolution for 15 regions (panels). Shaded areas indicate the ice-melt component of runoff at present-day (grey, 2001-2020) and 2081-2100 under SSP3-7.0 (red). Insets at top left show shifts in the timing of the seasonal high flow (in days) for the daily model compared to the 2001-2020 baseline.

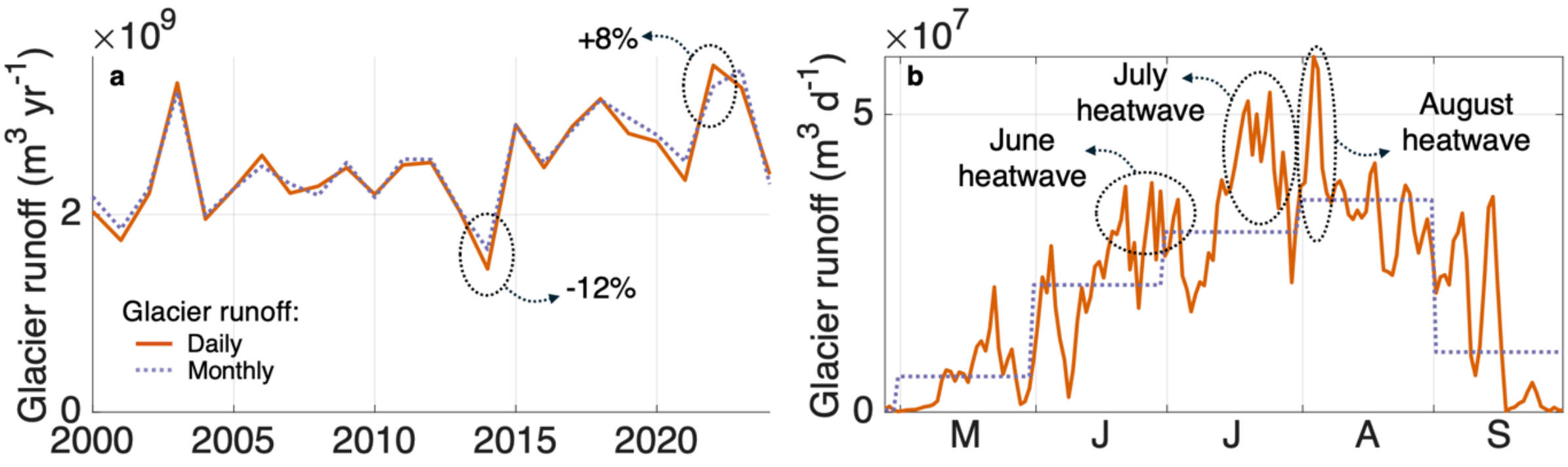

Extended Data Fig. 7: Total annual glacier runoff in Central Europe and an example summer cycle. **a** Modelled total annual glacier runoff in Central Europe for the period 2000–2024. **b** Modelled glacier runoff during the summer of 2022, which included three distinct heatwaves (Cremona et al., 2023).

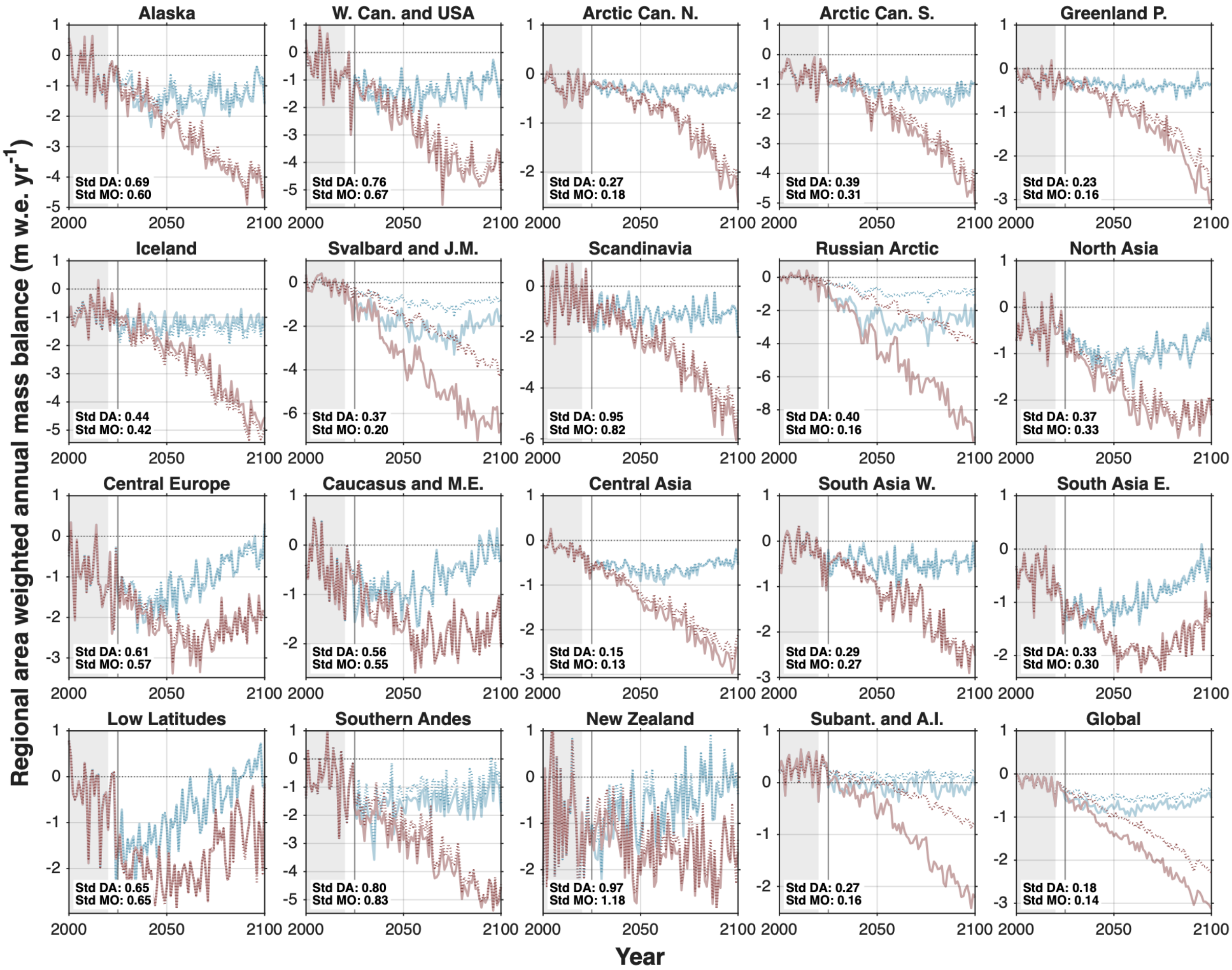

Extended Data Fig. 8: Regional area-weighted mass balance. Evolution of the regional area-weighted glacier mass balance over 2025–2100 under SSP1-2.6 (blue) and SSP5-8.5 (red). Lines show the multi-GCM mean across five GCMs for the daily model (solid lines) and monthly model (dashed lines). The standard deviation of the daily and monthly modelled mass balance over the 2000–2024 period is shown in the lower-left corner of each panel.

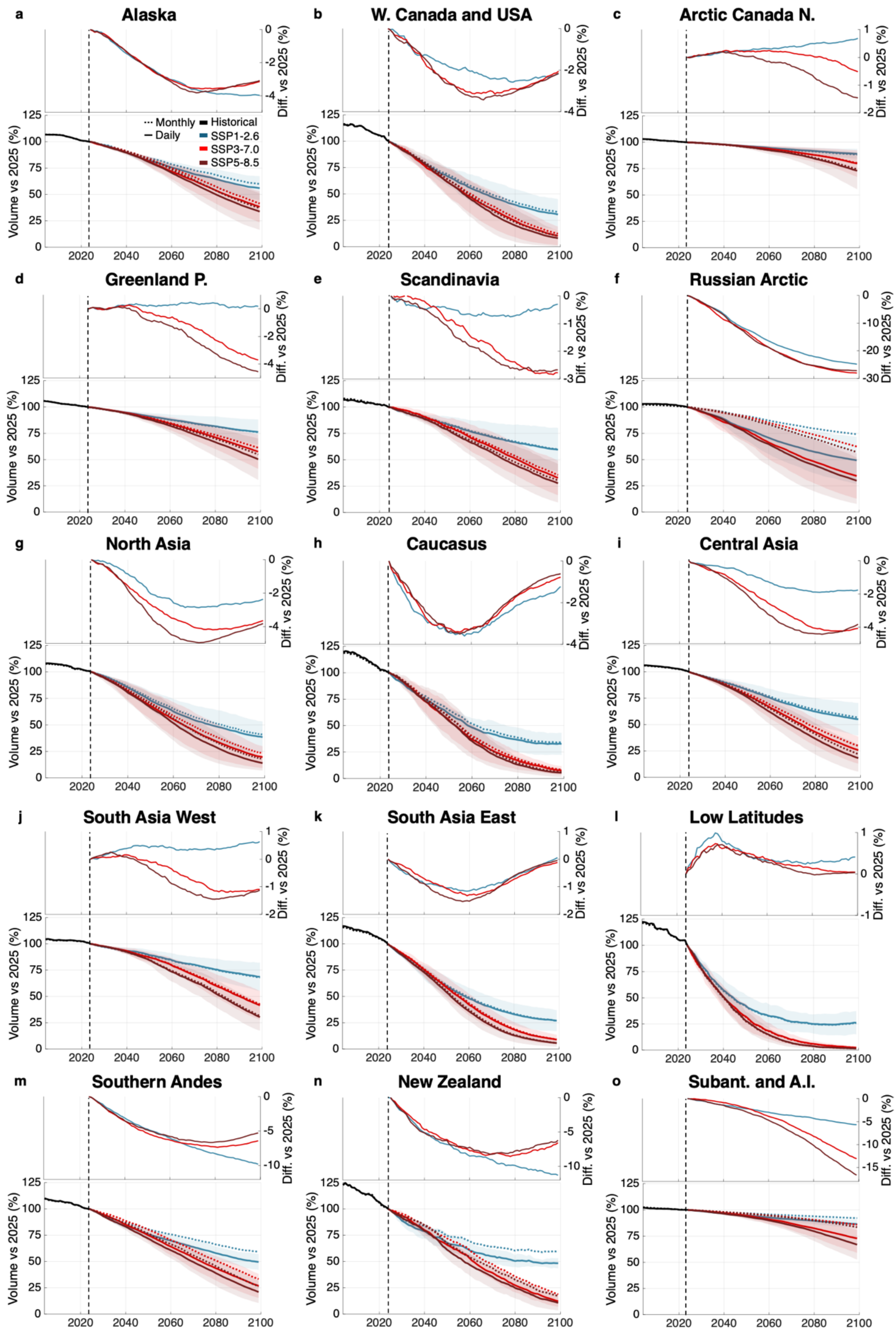

Extended Data Fig. 9: Regional ice volume evolution. Projected 21st-century glacier volumes (multi-model mean ±1 standard deviation) are shown as relative changes with respect to 2025. The upper panel highlights the difference in modelled volume between the daily and the monthly model.

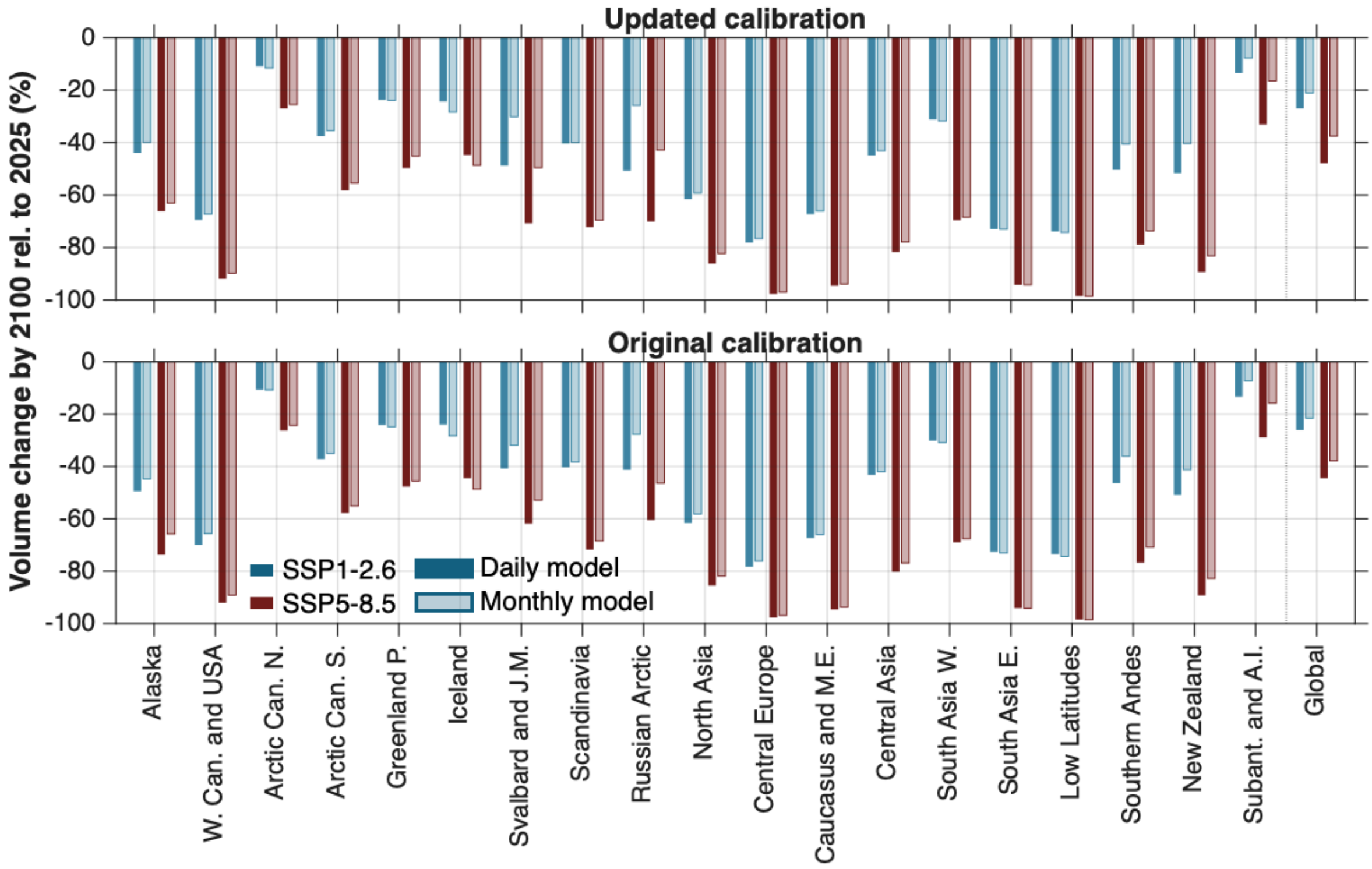

Extended Data Fig. 10: Regional ice volume by 2100 relative to 2025. Projected glacier volume in 2100 relative to 2025 for the daily and monthly models under SSP1-2.6 and SSP5-8.5. Results are shown for the updated calibration used as the standard in this study, in which T_{bias} and C_{prec} are cross-calibrated, and for the original calibration, in which all three calibration parameters are calibrated independently for the daily and monthly glacier models. Values indicate multi-GCM mean relative volume change for each region.

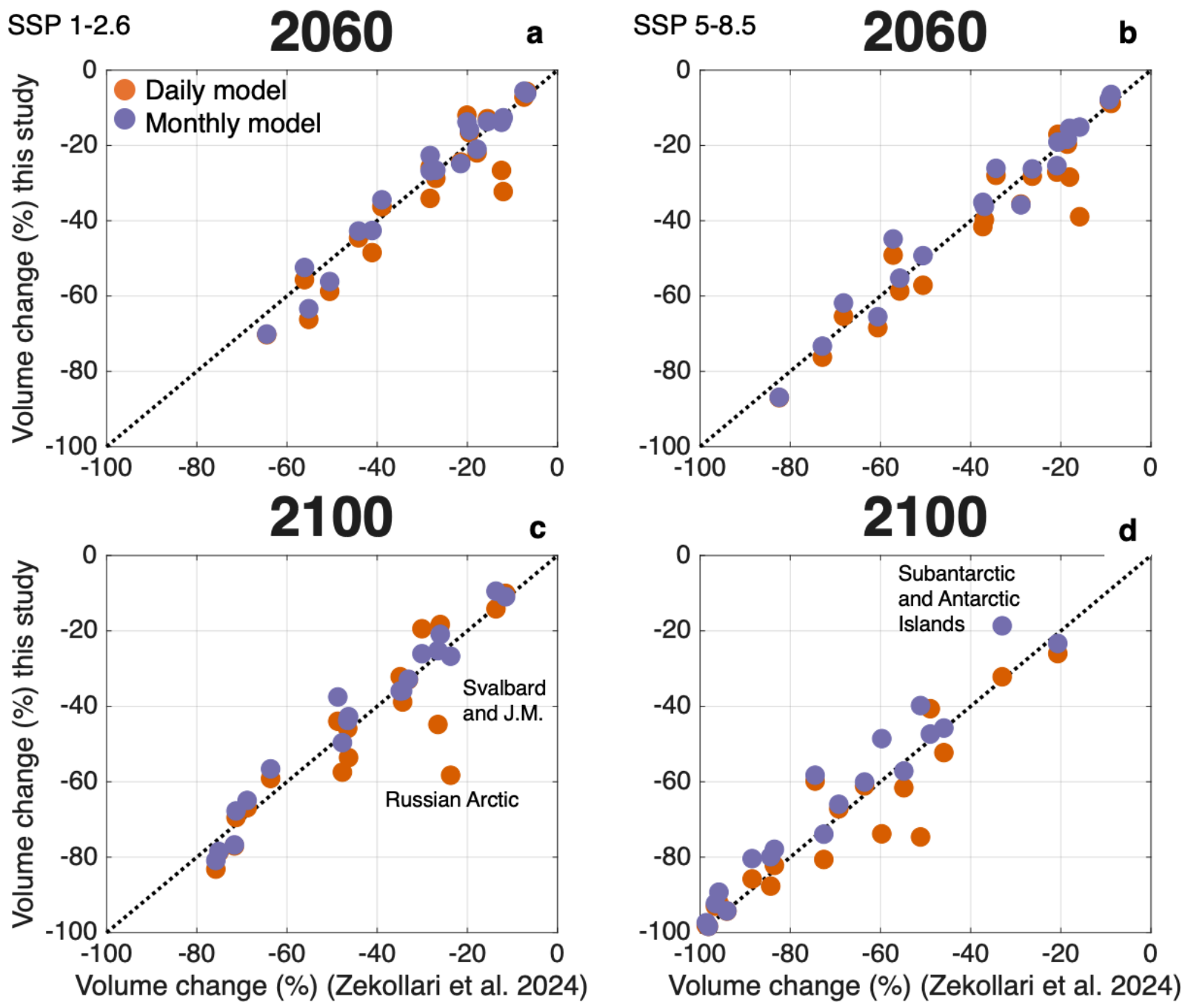

Extended Data Fig. 11: Comparison with previous GloGEM CMIP6 glacier-volume projections. Comparison of median relative glacier-volume changes (with respect to 2015) obtained in this study using the daily and monthly model (y-axis) against GloGEM results for the same glacier sample reported in Zekollari et al. (2024) for SSP1-2.6 (Panel a) and SSP5-8.5 (Panel b), shown for the years 2060 and 2100. Labelled regions indicate where differences between the previous projections and the new estimates are largest.

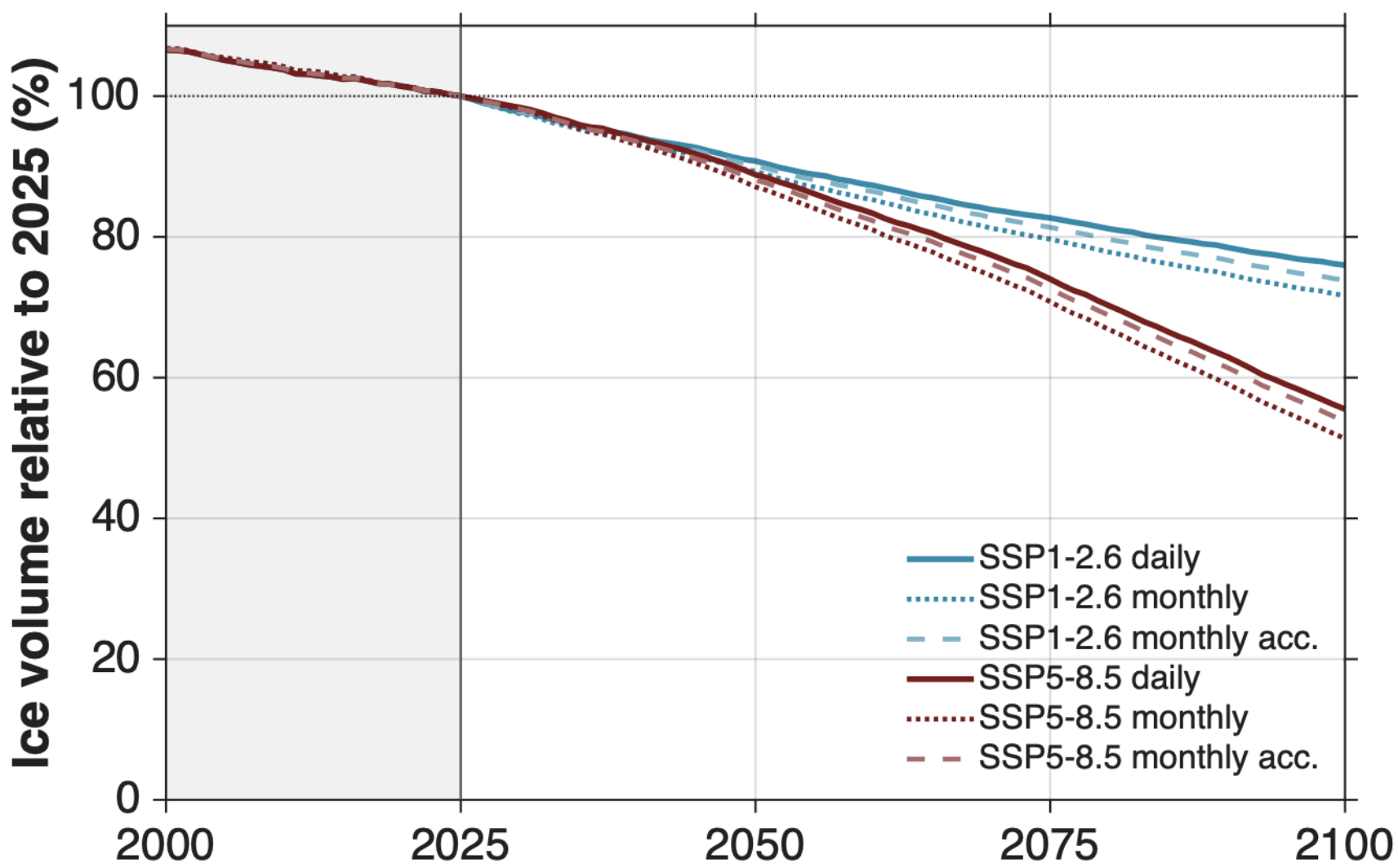

Extended Data Fig. 12: Regional ice volume relative to 2025 in Iceland. Projected glacier volume relative to 2025 for the daily and monthly models under SSP1-2.6 and SSP5-8.5 in Iceland. "Monthly acc" refers to the monthly model experiment in which accumulation is determined from the daily temperature evolution instead of the monthly mean temperature.
