# Supplementary material for: Resolving short-term meteorological variability amplifies projected glacier loss and sea-level rise
Source: Nat Commun. 2026 Oct 19;17:10696. doi: 10.1038/s41467-026-78260-y (PMC13680477; doi:10.1038/s41467-026-78260-y)
Supplement: Supplementary file 2 [file 41467_2026_78260_MOESM2_ESM.pdf]

nature portfolio

Peer Review File

Resolving short-term meteorological variability amplifies projected glacier loss and sea-level rise

Corresponding Author: Dr Lander Van Tricht

Parts of this Peer Review File have been redacted as indicated to remove third-party material.

This file contains all reviewer reports in order by version, followed by all author rebuttals in order by version.

Attachments originally included by the reviewers as part of their assessment can be found at the end of this file.

Version 0:

Reviewer comments:

Reviewer #1

(Remarks to the Author)
Please check attached file.

(Remarks on code availability)

Reviewer #2

(Remarks to the Author)
This manuscript by Van Tricht et al. presents a significant and timely methodological advance: the first global, scenario-based glacier projection using daily-resolution meteorological forcing. The core finding — that daily models project 5–10% greater global ice loss and 20–33 mm additional sea-level rise by 2100 relative to conventional monthly models — is broadly compelling and well-motivated. The manuscript is clearly written and the physical mechanisms are thoughtfully identified. However, several issues require major revision before acceptance. The cross-calibration design conflates calibration compromise with temporal-resolution effects; the monthly model is evaluated in a structurally disadvantaged form (fixed 5°C sub-monthly variability, coarse snowfall partitioning), inflating apparent daily-model improvements; high-latitude divergence results lack sufficient in situ validation; the human exposure extrapolation (4–10 million additional people at flood risk) is methodologically flawed and must be revised or removed; and code and data are not yet publicly accessible, precluding reproducibility assessment. The paper warrants major revision.

Detailed comments are attached!

(Remarks on code availability)

Reviewer #3

(Remarks to the Author)
Abstract
For clarity, I would say something like “Current global glacier models typically use temperature-index mass balance calculations forced with temporally aggregated meteorological data”.
This model cannot resolve surface-albedo feedbacks at any time scale. I think that what is intended is that daily resolution allows more rapid transitions between snow, firn and ice degree-day factors.
It should be noted that both approaches, which differ in 2025-2100 projections, are calibrated against 2000-2019 mass balances.

Improved mass balance representation
An estimate should be given for the uncertainty in WGMS mass balances. I believe that this will have 0.1 m w.e. order of magnitude, making the 0.02 m w.e. improvements in RMSE and MAE look very marginal. The improvements for extreme years are more significant and consistent with expectations for increasing temporal resolution.

Glacier runoff
The authors could consider substituting Extended Data Figure 5 for the map in Figure 2.
Would a spline fit to the monthly model runoff be very different to the daily model?

Projected ice volume
There is very large equifinality in calibrating precipitation correction factor, degree-day factor and temperature bias to 2000-2019 geodetic mass balances, giving different temperature sensitivities for equally good fits (Schuster et al. 2023, cited as 36). Claims about impacts of forcing frequency on projections would be more robust with an estimate of the resulting uncertainty.

Minor corrections:

29
“contributes an additional”

96
“mass balances aggregated over elevation bands”

184
“greyed out to avoid”

220
“low-relief, ice cap dominated regions”

324
“more glacier mass loss than”

351
Citations such as “Ice thickness distributions are taken from^63” are inelegant. “Ice thickness distributions are taken from Farinotti et al. (2017)^63” would be better.

404
“compare regional mass changes with independent regional estimates”

The formatting of equation 1 should be improved.

541
“assumed to be negligible”

551
“the glacier snout”

(Remarks on code availability)
Code and data not yet available

Version 1:

Reviewer comments:

Reviewer #1

(Remarks to the Author)
I think the manuscript has been substantially improved. The Methods section is now much clearer. I also pointed out several minor issues in the Results section, and I appreciate the authors' careful revisions in response to those comments.

(Remarks on code availability)

Reviewer #2

(Remarks to the Author)
I thank the authors for a careful and constructive response. The revision addresses the substance of all four of my major concerns. The independent-calibration sensitivity experiment (Extended Data Fig. 10), the regional calibration-parameter

statistics (Extended Data Table 5; Extended Data Figs. 4–5), the regional validation table and figure (Extended Data Table 2; Extended Data Fig. 2), the Iceland accumulation experiment (Extended Data Fig. 12), the removal of the flood-exposure extrapolation, and the public, documented, DOI-referenced release of GloGEM together make this a materially stronger and more transparent paper. I particularly welcome the added honesty about non-uniform regional performance, which is the kind of statement that makes a modelling paper trustworthy.
Some points remain. All are textual, none requires new simulations, and the first is the only one that touches the central claim. I recommend MINOR revision.

Remaining points

1. Framing of the abstract (lines 27–29, 33–35). The abstract states that the relevant mechanisms “cannot be resolved at a monthly time scale” and that resolving high-frequency variability “is essential” for robust projections. The revised discussion is more measured: the Iceland experiment (Extended Data Fig. 12) shows that a monthly model with daily-informed accumulation moves close to the daily model, and the authors conclude at lines 549–551 that simple corrections could improve monthly projections. I find the latter framing both more accurate and stronger result, since it diagnoses the mechanism rather than only reporting the difference. Please bring the abstract into line with it, and refer to the standard monthly GloGEM configuration rather than to monthly models in general. The title may warrant the same consideration.

2. Calibration underlying the validation (Fig. 1; Extended Data Table 2; Methods). Extended Data Table 2 reports RMSEs for the individually calibrated models, whereas the projections use the cross-calibrated configuration. The Fig. 1 caption does not state which configuration it shows, and the skill scores at lines 110–112 are unattributed. Since the argument runs from validation to projection, please state the calibration explicitly in both captions and in the Methods, and report the glacier-wide skill scores for the cross-calibrated configuration if they differ. Both parameter sets already exist.

3. Russian Arctic in the abstract (lines 25–27). The abstract now names the Russian Arctic and Svalbard as the regions with relative increases of up to 25%. Svalbard is well supported (14 glaciers, 270 observations). The Russian Arctic rests on one glacier and five observation-years, and the monthly model has the marginally lower RMSE there; the manuscript notes at lines 119–121 that the region allows only limited evaluation. Please lead with Svalbard, or qualify the Russian Arctic. New Zealand (lines 313–315) and the Low Latitudes are similarly weakly constrained and may deserve brief acknowledgement.

4. Comparison at lines 569–572. The 20–33 mm daily-minus-monthly difference is set against the 18 mm glacier contribution observed over 2000–2023. These are different quantities: a spread between two model configurations, and a measured historical signal. Please reframe the comparison, for instance by noting that the difference falls within the GCM ensemble spread shown in Fig. 4a, or omit it.

5. Extended Data Table 2. Please report MAE and R^2 alongside RMSE. The daily model’s claimed advantage lies in interannual variability, and R^2 is already quoted globally in the main text.

6. Extended Data Fig. 1 caption, and lines 90–92. The caption describes a fixed standard deviation of 5 °C without indicating that this is illustrative, and the main text describes a “fixed” standard deviation, which reads as spatially uniform rather than stationary in time. The Methods wording at lines 727–731 is clear and could be adopted in both places.

7. Data and code availability (lines 950–955). The text directs readers to GitHub, but reference 83 is a Zenodo software DOI and no repository URL is given; please add it so the documentation is reachable. A reserved DOI for the simulation outputs, cited now, would complete this.

(Remarks on code availability)

Reviewer #3

(Remarks to the Author)
The authors have adequately addressed my comments

(Remarks on code availability)

Open Access This Peer Review File is licensed under a Creative Commons Attribution 4.0 International License, which permits use, sharing, adaptation, distribution and reproduction in any medium or format, as long as you give appropriate credit to the original author(s) and the source, provide a link to the Creative Commons license, and indicate if changes were made.
In cases where reviewers are anonymous, credit should be given to 'Anonymous Referee' and the source.
The images or other third party material in this Peer Review File are included in the article's Creative Commons license, unless indicated otherwise in a credit line to the material. If material is not included in the article's Creative Commons license and your intended use is not permitted by statutory regulation or exceeds the permitted use, you will need to obtain permission directly from the copyright holder.
To view a copy of this license, visit https://creativecommons.org/licenses/by/4.0/

Reviewer 1

General comment	(R1.1)
This manuscript recalculates global glacier mass-balance variability using a daily time step instead of the previously employed monthly resolution. In doing so, precipitation, degree-day factors (DDF), and air temperature are calibrated so as to reproduce the 2000–2019 mass balances derived from the DEM. The authors then perform future projections and classify the causes of regional differences between the monthly and daily models into four types, which they discuss in detail. Glacier runoff is also computed. Their calibration procedure follows that of Huss and Hock (2015), with only a slightly different calibration range. The main differences from previous studies lie in the input datasets used. Specifically, the authors use ERA5 instead of ERA-Interim as the meteorological forcing, and they update the mass-balance data used for calibration from Gardner et al. (2013) to Hugonnet et al. (2021).	

We thank the reviewer for the detailed assessment of our manuscript. We agree that the manuscript builds on the GloGEM modelling framework and calibration strategy of Huss and Hock (2015) but would like to clarify one important point. The main methodological comparison in our study is not between simulations forced by different meteorological datasets or calibrated with different geodetic mass-balance products. Instead, both the monthly and daily model versions used here are forced with the same reanalysis product, ERA5-Land, calibrated against the same glacier-specific geodetic mass balance from Hugonnet et al. (2021), and use the same glacier geometry and ice-thickness information. This setup was chosen specifically to isolate the effect of temporal resolution as the only major difference between the two model configurations.

Reviewer 1 – Comment 2	(R1.2)
In the three-step calibration procedure, I understand that Tbias is initially set to zero when Cprec is determined, but from the Methods section it was not clear how the DDF is specified at this stage. If the procedure is identical to Huss and Hock (2015), i.e. a spatially uniform DDF is assumed, a key issue is that the calibrated Cprec will strongly depend on this initial choice of DDF. With the current approach, DDF is only allowed to vary when Cprec becomes smaller than 0.8 or larger than 2.5. DDF, however, typically varies by about a factor of two between regions. This implies that for the majority of glaciers (those with 0.8≤ Cprec ≤2.5) a single constant DDF value is effectively used. In my view it is not clear whether such a configuration can adequately reproduce the observed mass balance patterns for future projection.	

We thank the reviewer for this comment. We now have better clarified the calibration procedure in the Methods section. In the first calibration step, the precipitation correction factor is optimised while using fixed initial degree-day factors of $DDF_{snow} = 3$ mm d^{-1} °C^{-1} and $DDF_{ice} = 6$ mm d^{-1} °C^{-1}, following Huss and Hock (2015). These values provide the starting point of the calibration procedure and are taken mid-range of the values reported by Hock (2003). We acknowledge that the calibrated precipitation correction factor partly depends on this initial DDF choice. However, the calibration framework was designed to avoid overparameterisation and to first correct the climate-forcing component that is generally least well constrained: precipitation. Only when an acceptable match with observed mass balance cannot be achieved with standard DDFs and within the allowed precipitation-correction range

of $0.8 \leq C_{prec} \leq 2.5$, the DDF_s are adjusted, with DDF_{snow} varying between 1.75 and 4.5 mm d^{-1} °C^{-1} and DDF_{ice} and DDF_{firn} adjusted simultaneously.

We agree that DDFs can vary regionally, potentially by about a factor of two. However, in a global model such as GloGEM, this variability has to be balanced against the need for a robust and transferable calibration framework that can be applied consistently to all glaciers. More regionally or locally tuned models may allow for more specific DDF choices, but this would also introduce additional regional assumptions that are difficult to constrain consistently at the global scale, and against one dataset for calibration (Hugonnet et al., 2021). The aim of our study is not to develop a new calibration procedure, but to isolate the effect of moving from monthly to daily temporal resolution within an otherwise consistent global modelling framework. We also want to clarify that T_{bias} is not simply kept at zero throughout the full calibration. After the first calibration cycle, grid-cell-mean temperature biases are derived from all glaciers located within the same climate grid cell and imposed before repeating the calibration cycle. This ensures more spatially consistent T_{bias} values while still allowing C_{prec} and, where necessary, DDFs to adjust during the second calibration iteration. This is a new implementation of GloGEM which was first introduced in Zekollari et al. (2024).

To more clearly phrase the calibration, we have modified the Methods section in **lines 791-826**:

"During calibration, the model is run over the period 2000–2019 using an initial parameter set, after which parameters are adjusted sequentially in three steps:

In the first calibration step, the precipitation correction factor (C_{prec}) is tuned within the range 0.8–2.5 to account for biases in precipitation forcing. This parameter is prioritised, as precipitation (and thus accumulation) is typically one of the most uncertain variables in mountainous regions. During this step, the degree-day factors are kept fixed at their standard values.

If no satisfactory solution is found within the prescribed precipitation-correction range, DDF_{snow} is adjusted within the range 1.75–4.5 mm d^{-1} °C^{-1}. The degree-day factors for firn and ice are defined relative to that of snow, scaled by factors of 1.5 and 2, respectively, and therefore vary proportionally during this calibration step.

If still no satisfactory match is found after adjusting both C_{prec} and DDF_{snow} within their prescribed limits, a temperature bias (T_{bias}) is applied to correct for systematic temperature offsets in the climate input. This parameter has no predefined bounds.

The calibration procedure is repeated after an initial calibration cycle[14]. In this second iteration, grid-cell-mean temperature biases, derived from the first calibration across all glaciers within each grid cell, are imposed prior to recalibration. This ensures that temperature biases remain more consistent among glaciers within the same grid cell and prevents unrealistically large deviations between individual glaciers."

Reviewer 1 – Comment 3	(R1.3)
In the manuscript, in order to "obscure the interpretation of temporal-resolution effects", the authors average Cprec and Tbias between the monthly and daily models and then recompute the DDF for each temporal resolutions. However, I wonder whether this averaging step actually hides potentially important information. Before averaging, it would be very informative to explicitly analyse and discuss the differences in the individually calibrated parameters Cprec and Tbias between the daily and monthly setups. I think This could help clarify how much of the model behaviour stems from temporal resolution itself versus from compensating changes in the calibrated parameters. In my view, the calibration strategy proposed in this manuscript is not particularly innovative, and the resulting model does not seem well suited to reveal the fundamental processes governing glacier change.	

We thank the reviewer for this comment. We agree that the independently calibrated parameters may contain useful information and that it might be worth showing the differences between the daily and monthly model configurations for this particular setup. We have therefore added a new analysis of the calibrated values of C_{prec}, T_{bias} and the degree-day factors to Extended Data Figs. 4–5 and Extended Data Table 5 (see also bottom of this document). These figures and table show that, for most glaciers and regions, the calibrated parameter values are broadly similar between the daily and monthly configurations, although some regional differences occur.

In the text, we added in **lines 841-850**:

"For most glaciers and regions, the calibrated parameters obtained for the daily and monthly configurations are broadly similar (Extended Data Table 5; Extended Data Figs. 4–5). In some regions, the calibrated values for both the daily and the monthly version approach the prescribed parameter bounds. For example, in the Caucasus and Middle East, many glaciers reach the upper limit of C_{prec} *(2.5), whereas in several other regions* C_{prec} *values cluster near the lower limit (0.8), indicating that precipitation from the underlying ERA5-Land grid is corrected downward. The fact that these bounded values occur in both the daily and monthly versions indicate that there is no systematic difference between the two temporal resolutions. Extended Data Fig. 4 further shows that differences in* T_{bias} *between the daily and monthly calibrations are generally small compared to the regional spread in calibrated values."*

And on **lines 876-983**:

"Following the averaging of C_{prec} *and* T_{bias}*, the recalibrated DDF values are generally slightly higher in the daily model than in the monthly model (Extended Data Fig. 5). The largest differences occur in Svalbard, the Russian Arctic, and the Subantarctic and Antarctic Islands. In these regions, the independently calibrated daily model generally has lower* C_{prec} *and higher* T_{bias} *values than the monthly model. After averaging these parameters, the daily model therefore tends to produce relatively more accumulation and/or insufficient melt compared to what would be needed to reproduce the observed geodetic mass balance."*

To further test the influence of this choice, we now also compare volume projections using the averaged parameter set with projections based on the original, fully independent calibration. This comparison

shows that the differences between daily and monthly projections remain qualitatively consistent under both calibration strategies, although their magnitude can vary regionally (Extended Data Fig. 10, see also bottom of this document). This supports the interpretation that the reported differences between the daily and monthly model are not solely an artefact of the parameter-averaging procedure but rather due to the difference in temporal resolution. To present this additional test, we added in **lines 417-423**:

"We further tested the sensitivity of the volume projections to the calibration design by repeating the projections with an alternative calibration setup (the original, independent setup, in which C_{prec}, T_{bias} and the DDFs are calibrated separately for the daily and monthly model, see Methods). This experiment yields similar, or slightly smaller, daily–monthly differences, while the regional 21st-century volume changes remain broadly consistent (Extended Data Fig. 10). This indicates that the main regional patterns described above are robust to the choice of calibration strategy."

Finally, to evaluate whether both configurations reproduce observed mass-balance behaviour, we added a regional comparison between modelled annual mass balances and WGMS observations (Extended Data Fig. 2; Extended Data Table 2; see also R1.2 and R1.5). This comparison shows that both the daily and monthly configurations reproduce historical mass-balance patterns reasonably well in most regions, while also highlighting regions where observational constraints remain limited and model performance is weaker. This information is now added in **lines 115-121**:

"At the regional scale, however, improvements are not uniform (Extended Data Fig. 2; Extended Data Table 2). Most regions show similar or improved performance for the daily model, while others show little change or slightly degraded skill metrics, reflecting regional calibration uncertainties, differences in climate regimes and limited observational constraints. Regions with sparse mass-balance observations, such as the Russian Arctic and New Zealand, provide only limited opportunities for robust model evaluation, highlighting the continued need for long-term in situ observations in underrepresented glacier regions."

**Reviewer 1 – Comment 4**	**(R1.4)**
There are several places in the manuscript where the reference numbers do not seem to be correctly assigned.	

We thank the reviewer for pointing this out. We have carefully checked the reference numbering throughout the manuscript and corrected the citations where necessary.

**Reviewer 1 – Comment 5**	**(R1.5)**
Lines 98–109: When moving from monthly to daily data, the authors only demonstrate cases where the agreement with observations improves, but Fig. 1 suggests that there are also regions and elevation bands where the fit does not improve. Focusing on such regions and bands and identifying the reasons for the degraded performance could, in my view, reveal important shortcomings of the current calibration strategy.	

We agree that the global validation statistics shown in Fig. 1 do not fully capture the regional variability in model performance. We have therefore added a regional evaluation of modelled versus observed annual mass balances from WGMS (Extended Data Fig. 2, see also bottom of this document and our answer to R1.3) together with a summary of regional RMSE, MAE and R^2 values (Extended Data Table 2). These additions do indeed show that the daily model does not outperform the monthly model in every region. While most regions exhibit similar or improved skill metrics, some regions show only marginal improvements or slight degradation. We now discuss this explicitly in the manuscript on **lines 115-121**:

"At the regional scale, however, improvements are not uniform (Extended Data Fig. 2; Extended Data Table 2). Most regions show similar or improved performance for the daily model, while others show little change or slightly degraded skill metrics, reflecting regional calibration uncertainties, differences in climate regimes and limited observational constraints. Regions with sparse mass-balance observations, such as the Russian Arctic and New Zealand, provide only limited opportunities for robust model evaluation, highlighting the continued need for long-term in situ observations in underrepresented glacier regions."

**Reviewer 1 – Comment 6**	**(R1.6)**
Line 132: What exactly is meant by "original glacier outline" here? Is the term "original" actually necessary?	

By "original glacier outline", we intended to refer to the initial glacier extent as defined by RGI v6.0. This distinction is important because runoff is calculated not only over the glacierized area that remains through time, but also over areas that become deglacierised during the simulation. We agree that the term "original" was ambiguous and have therefore revised the text for clarity. The manuscript now reads in **lines 206-209**:

"Glacier runoff is defined as the sum of ice melt, snow melt and liquid precipitation over the initially glacierised area[44], minus refreezing (see Methods)."

**Reviewer 1 – Comment 7**	**(R1.7)**
Fig. 3: For the graphs within the "patterns" panels of Fig. 3, the righthand y-axis is labeled "Diff vs 2025 (%)", but it is not clear what this difference is taken between. Please explicitly state what is subtracted from what (e.g. "daily minus monthly relative to 2025" or similar) so that the definition of the percentage difference is unambiguous.	

This is a very good suggestion. We modified the y-axis label to "Da − Mo rel. to 2025 (%)" and clarified the caption as follows in **lines 413-415**:

"The upper half highlights the difference in modelled volume between the daily and the monthly model, with Da − Mo denoting daily minus monthly simulations and values expressed relative to the 2025 volume."

Reviewer 1 – Comment 8	(R1.8)
Fig. 3e: I found Arctic Canada South here, and not Alaska, Scandinavia and New Zealand	

The different regions are added at the bottom left of the panel.

Reviewer 1 – Comment 9	(R1.9)
Lines 188–240 ("Regional and global differences in projected ice volume changes" section): In this section, the authors discuss projected changes in glacier ice volume, which inherently include changes in glacier area. However, most of the mechanisms invoked to explain the regional differences relate to surface energy or massbalance processes at the glacier surface. I therefore wonder whether the manuscript would be clearer if the authors first focused on discussing differences in surface mass balance alone, before moving on to the resulting differences in ice volume change (which additionally involve geometric adjustment and area change).	

We agree with the reviewer that the interpretation of regional volume differences could benefit from an intermediate view on surface mass-balance differences before discussing the resulting ice-volume response. At the same time, we note that the regional annual mass balance is not a fully geometry-independent metric either, because it is area-weighted and therefore also affected by differences in glacier retreat and area loss. To address the reviewer's point, we added a new Extended Data Fig. 7 showing the evolution of regional area-weighted annual mass balance for the daily and monthly model. This figure shows that, in most regions, the daily model exhibits larger interannual variability during the calibration period (higher StD) and generally more negative mean mass balances in the projections. We also added a short clarification to the manuscript at the start of the results section on the regional volume changes. The text in **lines 320-323** now reads:

"These volume differences mainly arise from differences in climate forcing and model structure, which first affect the simulated surface mass balance (Extended Data Fig. 8) and then accumulate through time into differences in glacier geometry, area and volume."

Reviewer 1 – Comment 10	(R1.10)
L211-213 "where resolving daily variability produces steeper mass balance" In the Extended Figure 2, New Zealand has not larger mass balance gradient in the daily model.	

We thank the reviewer for pointing this out. We agree that New Zealand does not exhibit a noticeably steeper mass-balance gradient in the daily model (Extended Data Fig. 3), even though the region does show enhanced ice loss in the daily simulations during the 21st century. We have therefore revised the text to remove New Zealand from this specific discussion and to better distinguish between the different mechanisms contributing to regional differences. We modified the section i**n lines 341-351**:

"The second pattern appears for regions that retain a substantial ice volume throughout the 21st century and therefore exhibit a persistent, and in some cases increasing, divergence between the daily and

monthly model (Fig. 3e). This behaviour is observed in regions such as Alaska, Scandinavia, and the Southern Andes, where resolving daily variability produces steeper mass-balance gradients (Extended Data Fig. 3). The latter leads to enhanced mass turnover and a more rapid glacier response to climatic forcing[45,46]. While these effects accumulate over time, the differences in modelled ice volume remain generally moderate: by the end of the century and compared with the monthly model, the daily model typically projects additional ice-volume losses of 2–5% relative to the 2025 glacier volume (Fig. 3e). New Zealand also exhibits enhanced ice loss in the daily model, although this does not appear to be associated with a systematically steeper mass-balance gradient (Extended Data Fig. 3)."

**Reviewer 1 – Comment 11**	**(R1.11)**
Line 317: Is the expression "snowlines or snow cover datasets" really appropriate here? These datasets only provide information on the areal extent of snow cover, not on its mass. Wouldn't snow water equivalent (SWE) be more relevant for this purpose?	

We agree that snow cover and snowline observations do not directly provide information on snow mass or snow water equivalent (SWE). In principle, SWE observations would indeed provide a more direct constraint on glacier accumulation and mass balance. However, obtaining reliable SWE estimates over glaciers from satellite observations remains challenging, particularly at the global scale. In contrast, snow cover extent and transient snowline observations can be derived relatively robustly from optical satellite imagery and have therefore become increasingly used (e.g., Loibl et al., 2025; Cremona et al., 2025) as additional constraints in glacier mass-balance model calibration and validation. For this reason, we believe the current wording remains appropriate, although we have clarified that these datasets constrain the spatial extent of snow cover rather than its mass.

The in **lines 553-562** now reads:

"In parallel, the growing availability of new observational constraints, such as snowlines or snow cover datasets[56], provides new opportunities to better constrain the spatial extent of snowcover glacier models. This could help reduce some of the biases present in glacier models, e.g., those linked to model equifinality in which various parameter combinations allow reproducing similar observations[18]."

**Reviewer 1 – Comment 12**	**(R1.12)**
Line 452: The statement "applying distinct degree-day factors (DDFs) for snow, firn and ice, with fixed ratios of 1:1.5:2, respectively" requires a supporting reference.	

The choice of fixed ratios aligns with the one commonly made in GloGEM (Huss and Hock, 2015), which is in turn based on the typical ratio between DDF values reported for different regions on Earth (Hock, 2003). We now refer to this study in the text.

Reviewer 1 – Comment 13	(R1.13)
Lines 484–494: It would be important to explicitly state in which regions (or for which glaciers) a temperature adjustment was necessary. In addition, the manuscript lacks a clear description of which specific mass-balance quantity the three parameters were calibrated against (e.g. glacier-wide mean annual balance versus elevation-band mass balance). This is a crucial methodological detail and should be described more explicitly.	

Regarding the temperature adjustment, the reviewer is correct that the original manuscript did not provide information on the spatial distribution of the calibrated parameters. Following this suggestion, and similar comments from other reviewers, we have added a comparison of the calibrated values of C_{prec}, DDF_{snow}, and T_{bias} across all regions in Extended Data Figs. 4-5 and Extended Data Table 5. We refer to our answer in R1.3 for more details. We also agree that the calibration parameters and the calibration target should be stated more explicitly. The parameters were calibrated against the glacier-wide mean specific geodetic mass balance over the period 2000–2019 from Hugonnet et al. (2021), rather than against elevation-dependent mass-balance observations because such elevation-band information is not be available at the global scale. To avoid ambiguity, we have clarified this in the revised manuscript in **lines 783-785**:

"Model calibration ensures that the modelled glacier-wide mass balance reproduces the glacier-specific geodetic mass-balance estimates of Hugonnet et al. (2021)[39] for 2000–2019 within ca. ±0.1 m w.e. yr^{-1}."

Reviewer 2

General comment	(R2.GC)
This manuscript presents the first global, scenario-based glacier evolution projection using a daily resolution version of the Global Glacier Evolution Model (GloGEM), applied to all glaciers larger than 5 km^2 (~94% of global ice volume). The authors compare daily and monthly model configurations under three SSP scenarios (SSP1-2.6, SSP3-7.0, SSP5-8.5) forced by five CMIP6 GCMs, and quantify the implications of temporal resolution for projected mass balance, glacier runoff, ice volume evolution, and sea-level rise.<br><br>The central finding is that resolving daily meteorological variability leads to systematically greater projected glacier mass loss (5–10% more by 2100), with regional relative increases exceeding 25%, contributing an additional 20–33 mm to global mean sea-level rise compared to monthly forcing. The topic is timely and relevant to the glaciological, hydrological, and climate impact communities. The cross-calibration approach to isolate the temporal-resolution effect is methodologically thoughtful, and the identification of four distinct regional divergence patterns offers physical insight beyond a simple aggregate comparison. The validation against WGMS and Glambie datasets is appropriate, and the demonstration of improved performance during extreme years (e.g., 2022 Alpine melt, 2024 Svalbard) is compelling.<br><br>Nevertheless, several methodological choices, interpretational claims, and reproducibility concerns require attention before the manuscript is suitable for publication. The headline performance improvements in routine years are modest, the comparison may inadvertently treat the monthly model as a strawman, and several quantitative claims (particularly the sea-level rise extrapolation to flood exposure) require more careful framing. I therefore recommend major revision, with the major and minor comments detailed below.	

We thank the reviewer for the thorough and constructive assessment of our manuscript. We appreciate the recognition of the novelty of this work, the methodological effort to isolate the effect of temporal resolution, and the relevance of the results. We also acknowledge the concerns raised and have tackled these by carefully revising our manuscript. In particular, we now provide additional information on the calibration parameters, further justify our cross-calibration strategy, and removed the discussion of sea-level rise and exposure estimates where appropriate. We believe that these revisions have substantially improved the clarity, robustness, and reproducibility of the study.

Reviewer 2 – Comment 1	(R2.1)

The authors implement a cross-calibration procedure (lines 505–524) in which C_prec and T_bias are independently calibrated for both configurations, then their mean values are imposed on both daily and monthly simulations, with only the DDFs subsequently re-calibrated separately. The stated goal is to isolate the impact of temporal resolution. However, this design raises several concerns:

1. The averaged parameters are optimal for neither configuration. By construction, the resulting C_prec and T_bias are a compromise that does not reflect the best-fit calibration for either model. This means the reported daily-vs-monthly differences may partly reflect the imposition of suboptimal parameters on one or both configurations, rather than purely the resolution effect.

2. No sensitivity analysis is provided. It would be valuable to know how results differ when each configuration is independently calibrated (the "natural" approach) vs. the crosscalibrated approach. Without this comparison, readers cannot assess whether the headline conclusions are robust to the calibration design.

3. DDFs are re-calibrated, but C_prec is not, despite precipitation phase being central to several mechanisms. For the Iceland "reversed divergence" pattern (lines 232–241), the authors argue that monthly averaging overclassifies snowfall and biases calibration. If this is true, then fixing C_prec at an averaged value may carry over the bias from the monthly calibration into the daily configuration.

4. The Iceland mechanism appears post-hoc. The argument that monthly averaging "overclassifies snowfall, and thus overestimates accumulation. This bias propagates into calibration, yielding parameters that increase glacier mass-balance sensitivity to future warming" is plausible but unsupported by direct diagnostic evidence. The authors should provide a quantitative breakdown of the calibrated parameters for Iceland under both configurations (independent calibration), and ideally for at least one region from each of the four divergence patterns.

Recommended actions:
(i) Add a supplementary analysis comparing results from independently calibrated daily and monthly configurations against the cross-calibrated baseline.
(ii) Provide regional summary tables of calibrated parameters (C_prec, T_bias, DDF_snow) for both configurations.
(iii) Explicitly discuss the calibration-design trade-offs in the Methods section, including how Iceland's parameters differ between independent and cross-calibration.

We thank the reviewer for this very detailed and constructive comment. Our primary motivation for the cross-calibration approach is to isolate the effect of temporal resolution. Allowing the daily and monthly models to retain independently calibrated values of C_{prec} and T_{bias} would imply that the two model configurations are effectively forced by different climatic conditions (cf. answer to review comment R1.3). Both factors are known to exert a strong influence on glacier evolution and could therefore obscure the specific contribution of temporal resolution to the projected differences. Following the reviewer's suggestions, we have expanded our analyses of the calibration procedure in three ways:

1. We performed an additional sensitivity analysis comparing glacier evolution obtained with independently calibrated daily and monthly model configurations against the cross-calibrated setup used in the main manuscript. The results are presented in the Extended Data Fig. 10 (see also bottom of this document) and discussed in **lines 417-423**:

   "We further tested the sensitivity of the volume projections to the calibration design by repeating the projections with an alternative calibration setup (the original, independent setup, in which C_{prec}, T_{bias} and the DDFs are calibrated separately for the daily and monthly model, see Methods). This experiment yields similar, or slightly smaller, daily–monthly differences, while the regional 21st-century volume changes remain broadly consistent (Extended Data Fig. 10). This indicates that the main regional patterns described above are robust to the choice of calibration strategy."

2. We now provide a regional comparison of the calibrated values of C_{prec}, T_{bias}, and DDFs for both the daily and monthly model configurations (Extended Data Table 5; Extended Data Fig. 4–5). This allows readers to assess the magnitude and spatial patterns of the calibration differences between both setups. For more details we refer to our answer in **R1.3, pages 4–5**.

3. We expanded the Methods sections to explicitly discuss the rationale, advantages, and limitations of the cross-calibration procedure. The text on **lines 876-883**:

   "Following the averaging of C_{prec} and T_{bias}, the recalibrated DDF values are generally slightly higher in the daily model than in the monthly model (Extended Data Fig. 5). The largest differences occur in Svalbard, the Russian Arctic, and the Subantarctic and Antarctic Islands. In these regions, the independently calibrated daily model generally has lower C_{prec} and higher T_{bias} values than the monthly model. After averaging these parameters, the daily model therefore tends to produce relatively more accumulation and/or insufficient melt compared to what would be needed to reproduce the observed geodetic mass balance."

   And in **lines 884-888:**

   "This cross-calibration design involves a trade-off. The averaged C_{prec} and T_{bias} values do not necessarily represent the optimal parameter combination for either the daily or the monthly model when considered independently. Instead, they enforce a common climate forcing between temporal resolutions, at the cost of partly moving both models away from their independently calibrated optimum. We use this setup to isolate temporal-resolution effects, rather than to identify a unique best-fit parameter set for each model configuration."

**Reviewer 2 – Comment 2**	**(R2.2)**

The authors propose that daily modelling captures processes that "a monthly resolution cannot reproduce" (line 290). However, several aspects of the monthly model comparison appear to penalise it more than is necessary:

1. Fixed sub-monthly temperature standard deviation of 5°C. The authors themselves note (lines 308–311) that "assumptions of stationary sub-monthly temperature variability are increasingly inconsistent with observed changes in the variance of temperature distributions under climate change." Yet this stationary σ is retained as the basis for comparison. If σ were updated using GCM-derived sub-monthly variability (which is, in principle, available from the same daily GCM outputs used to force the daily model), how much of the daily-monthly gap would close?

2. Precipitation phase based solely on monthly mean temperature in the monthly model. Extended Data Fig. 1 vividly illustrates the issue: in a warm month with mean T > 0 °C, the monthly configuration yields zero snowfall. This is a known limitation that simpler statistical adjustments (e.g., applying the same temperature PDF to precipitation-phase partitioning) could partially address. The comparison would be more compelling if it included an "improved monthly" intermediate configuration that applied the sub-monthly PDF to precipitation phase as well.

3. Monthly surface-type updating is a model choice, not an intrinsic property of monthly forcing. A monthly-forced model could conceptually update surface type more frequently using sub-monthly logic. The fact that the standard GloGEM does not is a structural choice that compounds the resolution penalty.

The combined effect is that some of the reported daily-vs-monthly differences may reflect implementation choices in the monthly model rather than a fundamental limitation of monthly forcing. The authors note this implicitly (lines 308–315) but do not test it.

Recommended actions:
(i) Implement and report at least one "improved monthly" sensitivity experiment in which the monthly precipitation phase uses the assumed sub-monthly temperature PDF (matching the PDD calculation).
(ii) Discuss explicitly the difference between the "resolution effect" (temporal granularity of inputs) and the "implementation effect" (specific choices in the monthly GloGEM).
(iii) Soften the language in the abstract and conclusions to reflect that the comparison is between the daily GloGEM and the current standard monthly GloGEM, not a generic monthly model.

We thank the reviewer for these thoughtful comments. Regarding point 1, we would like to clarify that the monthly configuration used here does not rely on a globally fixed sub-monthly temperature standard deviation of 5°C (which is given as an example in Extended Data Fig. 1). Instead, the sub-monthly temperature variability used for the positive-degree-day calculation is grid-cell dependent and derived from daily temperature variability during the historical period based on ERA5-Land. It is true, however, that this variability is stationary in time. We argue that allowing it to evolve with projected changes in daily temperature distributions could either reduce or increase part of the difference between the monthly and daily simulations, as it is not clear how the variability will actually evolve.

We agree that alternative monthly formulations could be developed, for example by using changing sub-monthly temperature variability, applying sub-monthly temperature distributions to precipitation-phase partitioning or by updating surface type more frequently within the monthly time step. However, developing and calibrating such an improved monthly model is beyond the scope of this study. Our aim here is to evaluate the added value of a newly developed daily version of a global glacier evolution model relative to the established monthly framework used in previous global projections, while keeping the comparison internally consistent.

We added in **lines 535-542**:

"In this study, we identified several mechanisms that impact glacier projections, which provide potential pathways for improving monthly models. Assumptions of stationary sub-monthly temperature variability based on reanalysis data (see Methods) are increasingly inconsistent with observed changes in the variance of temperature distributions under climate change[36,53,54,55]. Accounting for such non-stationarity would require daily GCM output, or other robust information on future changes in sub-monthly temperature variability, to be readily available; however, this is not always the case for all models, scenarios or long-term projection experiments."

Introducing an additional intermediate monthly model would also require new methodological choices, calibration and validation, and would make it more difficult to isolate the behaviour of the two currently established configurations. Moreover, applying a sub-monthly temperature distribution to accumulation is not necessarily a straightforward improvement. Such an approach assumes that precipitation is distributed independently of sub-monthly temperature variability, which is not generally valid. If we come back to Iceland, where a large fraction of precipitation may occur during relatively warm conditions, while colder days are often drier. In such cases, applying an unweighted temperature distribution to precipitation-phase partitioning could overestimate snowfall as well. A physically more consistent correction would require information on the covariance between temperature and precipitation occurrence, ideally at the grid-cell or regional scale. It would also require assumptions about whether these relationships remain stationary under future climate change. Given these uncertainties, we have decided not to introduce or explore such an additional correction here. Instead, we consider the more robust way forward to be the transition towards a fully daily model configuration, which is becoming increasingly feasible and is illustrated in this study by our implementation of the daily simulations.

Following the reviewer's suggestion (i) and also building on R2.1, we performed an additional sensitivity experiment for Iceland in which accumulation in the monthly model is determined using the daily temperature evolution, while all other aspects of the monthly model structure are left unchanged. This allows us to assess how much of the daily–monthly difference can be attributed specifically to precipitation-phase partitioning. We added the results as Extended Data Fig. 12 (see also bottom of this document). This experiment shows that the projected ice-volume evolution of this modified monthly setup is more aligned with the daily model than with the standard monthly model, confirming that precipitation-phase partitioning is the main driver of the reversed daily–monthly divergence in Iceland. We added a short explanation of this sensitivity experiment to the manuscript in **lines 544-551**:

"In an additional sensitivity experiment for Iceland, we ran the monthly GloGEM model with accumulation determined from the daily temperature evolution, while the remaining monthly model structure was left unchanged (Extended Data Fig. 12). In that case, the resulting ice-volume evolution shifts much closer to the daily model, confirming that precipitation-phase partitioning is the main driver

of the reversed daily–monthly divergence in Iceland (see Fig. 3g). This suggests that relatively simple corrections for sub-monthly temperature variability and precipitation-phase partitioning could improve monthly glacier projections.”

Regarding points ii and iii, we agree that part of the difference between the daily and monthly simulations reflects not only the temporal granularity of the climate input, but also structural choices in the established monthly GloGEM implementation. We have therefore revised the text to distinguish more clearly between effects related to the temporal resolution and effects related to the specific monthly model formulation and performed an additional sensitivity experiment. We have softened the wording at several instances to explicitly refer to the GloGEM monthly model and in the introduction as follows on **lines 20-25**:

“Here, we develop a daily-resolution version of the Global Glacier Evolution Model (GloGEM), which we apply globally to all glaciers larger than 5 km² and compare against the standard monthly GloGEM configuration. This daily model more closely reproduces annual mass balance observations than its monthly configuration and provides a more detailed representation of glacier runoff patterns and their temporal changes. “

And in the concluding statements in **lines 566-568**:

“By resolving daily meteorological variability, our simulations consistently project more glacier mass loss than the current monthly GloGEM configuration across most regions, highlighting the potential importance of temporal resolution for downstream impacts.”

**Reviewer 2 – Comment 3**	**(R2.3)**
The mass-balance validation against WGMS observations (Fig. 1) shows overall modest improvement (glacier-wide RMSE 0.63 → 0.61 m w.e., R. 0.71 → 0.75). The authors emphasise that the daily model substantially outperforms in extreme years (RMSE 0.98 → 0.83 m w.e. for Å}2σ events), which is convincing. However, the largest projected divergences between daily and monthly models occur in highlatitude, ice-cap-dominated regions: Svalbard, Russian Arctic, and Antarctic/sub-Antarctic Islands (Fig. 3f; up to 20–25% relative difference by 2100). These regions are precisely where in situ WGMS validation is sparse:<br><br>1. The WGMS dataset is acknowledged to be "biased towards smaller, mid-latitude glaciers" (line 401–402). This means the regions where the daily model claims the largest improvements are validated the least directly.<br><br>2. The Svalbard 2024 example is compelling but anecdotal. A single extreme year for a single region does not constitute a robust validation of a ~20–25% projected divergence over the 21st century in the broader Russian Arctic and Antarctic regions.<br><br>3. The Antarctic/sub-Antarctic anomaly is concerning. The authors note (lines 261–265) that both daily and monthly models project smaller losses than previous global assessments in these regions, and attribute this to "forcing and model uncertainty [that] can outweigh temporal-resolution effects in cold, low-melt regimes." If this is so, then the claimed 20–25% divergence in Antarctic/sub-Antarctic glaciers under SSP5-8.5 should be presented with greater caution — the daily model's apparent improvement in these regions may rest on a forcing baseline that is itself biased.	

4. Cumulative regional mass balance comparison (Fig. 1c) uses Glambie, which is an inverse comparison: simulations restricted to glaciers >5 km. are compared to observations including all glaciers. This methodological mismatch could mask or amplify apparent differences and should be discussed.

Recommended actions:
(i) Add a regional breakdown of validation statistics (RMSE, MAE, R.) for each RGI region for both models, with the number of validation glaciers and observation-years per region.
(ii) Explicitly flag the regions where validation data are sparse (Russian Arctic, Antarctic/sub-Antarctic) and temper claims accordingly.
(iii) For the Antarctic/sub-Antarctic anomaly, provide an explicit discussion of whether the projected daily-vs-monthly divergence is robust given the underlying baseline mismatch with literature.
(iv) Where possible, supplement validation with remote-sensing-based seasonal snow line observations (the authors mention these in lines 315–317 as a future opportunity; integrating even a few representative examples would strengthen the high-latitude case).

To address both suggestions (i) and (ii), we added a new figure (Extended Data Fig. 2, see also bottom of this document) and a new table (Extended Data Table 2) in which we show the validation of the mass balances at regional scale. We refer to our answer in **R1.5 on pages 5–6** for more information. We agree that the Antarctic and Sub-Antarctic region requires caution because both the daily and monthly model configurations project substantially smaller ice losses than previous global assessments. As noted in the manuscript, glacier evolution in this region is highly sensitive to uncertainties in surface climate forcing (ensemble of GCMs used) and frontal ablation, both of which can exert a stronger influence on projected ice loss than temporal-resolution effects alone. To clarify this point, we expanded the discussion in the manuscript **on lines 371-379**:

"For these regions, the daily model projects up to ~15% more relative ice loss for the period 2025–2060 (vs. monthly model), which increases to up to ~20–25% for 2025–2100 (Fig. 3f). These values should be interpreted as upper-end estimates, as these regions are particularly sensitive to calibration choices (see Methods and Extended Data Fig. 4–5). These differences are also more pronounced than for other high-latitude regions, such as Arctic Canada North, Arctic Canada South and Greenland periphery (Fig. 3e), where the slightly broader elevation ranges and weaker surface-type feedbacks limit the steepening of mass-balance gradients (for daily vs. monthly model)."

Regarding the use of the GlaMBIE dataset (point 4), we agree that the difference in spatial coverage between the simulations and the observations can affect the interpretation of the regional comparison. In our simulations, only glaciers larger than 5 km^2 are included, whereas the GlaMBIE estimates represent regional glacier mass changes for the full glacier population. In regions where our modelled glacier sample covers only a limited fraction of the total glacier area, differences between modelled and observed regional mass changes may therefore partly reflect differences in spatial coverage rather than model performance alone. Conversely, the comparison is more robust in regions where the >5 km^2 glacier sample represents a large fraction of the regional glacier area. We have added this directly to the manuscript text in **lines 136-140**:

"For the regional comparison, differences in spatial coverage should be kept in mind, as our simulations are restricted to glaciers >5 km² while the GlaMBIE estimates represent the full regional glacier inventory. Nevertheless, the improvement from using daily rather than monthly forcing remains apparent, particularly for extreme mass-balance years."

Concerning point (iii), we have slightly reformulated the text to clarify that the results for the Subantarctic and Antarctic Islands are associated with the largest uncertainty. The revised text in **lines 443-448** now reads:

"The Antarctic and Sub-Antarctic glaciers form a notable exception, for which both the daily and the monthly models project substantially smaller volume losses than reported in previous global assessments, especially under the SSP5-8.5. Here, projected differences are particularly sensitive to the representation of both surface climate in the reanalysis forcing and frontal ablation[50,51], highlighting that forcing and model uncertainty can outweigh temporal-resolution effects in cold, low-melt regimes."

Finally, regarding point (iv), we agree that additional remote-sensing-based seasonal snowline observations would be valuable for model validation. However, this is beyond the scope of our study and is currently being addressed in a separate manuscript led by our co-author Alex von der Esch, which is currently under review.

**Reviewer 2 – Comment 4**	**(R2.4)**
In the final section (lines 325–334), the authors translate the additional 20–33 mm of sea-level rise from daily-vs-monthly modelling into "4–10 million additional people exposed to flooding by 2100" using a linear extrapolation of ~200,000–300,000 people per mm of SLR.<br><br>This claim<br>warrants substantial revision for several reasons:<br>1. Linear extrapolation is unlikely to hold at this scale. The cited exposure rate (Kulp & Strauss, 2019; Nicholls et al., 2021) is derived from current population distributions and present-day coastal topography. Applying it to a 20–33 mm increment on top of a much larger projected SLR (the daily model's 86–157 mm glacier contribution alone, plus thermal expansion, ice sheets, and land water storage) ignores that exposure responses to SLR are typically nonlinear and depend on local topography, adaptation, demographic change, and concurrent processes such as subsidence.<br><br>2. The marginal vs. total framing is misleading. Stating that "an additional 4–10 million people are exposed to flooding by 2100" implies that this is a discrete, attributable population. In reality, this is a marginal sensitivity estimate within a much larger uncertainty envelope. The comparison to the 18 mm of glacier-driven SLR observed over 2000–2023 (line 328) is rhetorically powerful but mixes a modelling choice difference with an observed historical increment, which are not directly comparable.<br><br>3. The framing risks overshadowing the methodological contribution. The paper's strongest contribution is methodological — demonstrating that temporal resolution materially affects projections. The flood-exposure extrapolation pushes into impact assessment territory that requires additional caveats not provided here.	

4. Uncertainty quantification is missing. No uncertainty range is given for the exposure estimate, despite the underlying SLR estimate having a Å}1 SD spread that overlaps zero in some scenarios (Fig. 4a uncertainty envelopes).

Recommended actions:
(i) Either remove the flood-exposure extrapolation or substantially
temper it. If retained, present it explicitly as a first-order sensitivity estimate, include uncertainty bounds, and acknowledge nonlinearities and adaptation.
(ii) Reframe the comparison to historical glacier-driven SLR (line 328) to avoid conflating a model-choice difference with an observed increment.
(iii) Consider moving impact extrapolations to a discussion paragraph rather than the conclusion, where the methodological contribution should remain central.

We agree that the flood-exposure extrapolation involves substantial uncertainties and assumptions that are beyond the scope of the present study. We have therefore removed this estimate from the manuscript and revised the conclusion to retain the focus on the methodological contribution, namely the effect of temporal resolution on glacier projections.

**Reviewer 2 – Comment 5**	**(R2.5)**

Lines 23–24: "relative increases exceeding 25% at the regional scale". Specify which region(s) and scenario this refers to in the abstract.

We thank the reviewer for pointing this out. We have now clarified in the abstract that regional relative increases up to 25% occur in the Russian Arctic and Svalbard, while keeping the wording concise and avoiding excessive scenario-specific detail. We changed this in text by adding in **lines 25-27**:

"We show that accounting for short-term meteorological variability leads to systematically greater projected glacier mass loss, with relative increases over 2025-2100 of up to 25% for the Russian Arctic and Svalbard."

**Reviewer 2 – Comment 6**	**(R2.6)**

Line 27: "26 ± 10% under low-emission scenarios and 47 ± 17% under high-emission scenarios". State that these are ensemble-mean ± 1 SD across GCMs.

We have revised the sentence to explicitly state that the reported uncertainties represent the standard deviation across GCMs. We changed the text accordingly in **lines 30-31**:

"...expressed as the ensemble mean ± 1 Std across GCMs."

Reviewer 2 – Comment 7	(R2.7)
Lines 49–50: The phrasing "such approaches have not yet been used for global, scenario based projections of 21st-century glacier change" should explicitly contrast with the cited daily-forced studies (refs. 21–24) by noting their scope (regional, single-scenario, etc.) for clarity.	

We have clarified the scope of the previously cited daily-forced studies. These studies have demonstrated the value of daily forcing for selected regions, river basins, individual scenarios or limited glacier samples, but have not yet provided global, scenario-based projections of 21st-century glacier change.

We changed the text which now reads in **lines 62-66**:

"While a few number of large-scale glacier evolution models have been forced with daily data[21,22,23,24], these applications have so far focused on selected regions or river basins, individual scenarios, or limited glacier samples. Daily forcing has not yet been applied in a global glacier model to produce scenario-based projections of 21st-century glacier change."

Reviewer 2 – Comment 8	(R2.8)
Line 55: "transient albedo feedbacks"? Given that GloGEM uses a degree-day approach without explicit albedo, clarify how the daily model captures albedo effects (presumably indirectly through surface-type switching).	

We agree that the term "transient albedo feedbacks" could be misleading, as GloGEM uses a degree-day approach and does not explicitly resolve albedo. What we intended to refer to is the effect of rapid surface-type transitions, for example from snow-covered to ice-covered conditions, which is already mentioned in the sentence. We have therefore removed the albedo example to avoid confusion.

Reviewer 2 – Comment 9	(R2.9)
Lines 64–65: Justify the 5 km² threshold more quantitatively. What fraction of regional ice volume is excluded in each RGI region? This is particularly relevant for regions dominated by smaller glaciers (e.g., Central Europe, New Zealand, Low Latitudes).	

We thank the reviewer for this helpful suggestion. To address this, we added a new Extended Data Table 1 that reports, for each RGI region, the number of modelled glaciers larger than 5 km², their total area and volume, and the fraction of regional glacier area and volume represented by this subset. This shows explicitly how much of the regional glacier inventory is included in the simulations and how much ice volume is excluded by the 5 km² threshold. The table highlights that, although many small glaciers are excluded by number, the modelled subset captures the dominant fraction of glacier volume in all regions except Low Latitudes. The text now reads in **lines 602-608**:

"We simulate all glaciers from the Randolph Glacier Inventory v6.0[44] larger than 5 km², comprising

12,571 glaciers, or about 6% of the global total by number. These glaciers account for 148,075 km³ of ice (94% of the global glacier ice volume) and 583,189 km² of glacierised area (83% of the global glacier area) (Extended Data Table 1). Although this threshold excludes a large number of small glaciers, especially in regions with many small ice bodies like High Mountain Asia, our aim is not to simulate every individual glacier, but to isolate the effect of daily versus monthly forcing within a consistent global modelling framework.“

**Reviewer 2 – Comment 10**	**(R2.10)**
Lines 363–364: Specify whether the "nearest land grid cell" approach causes systematic biases for glaciers near coastlines or in steep topography.	

We have clarified that the use of the nearest land grid cell is required because ERA5-Land only provides values over land surfaces. For glaciers close to coastlines, especially large or tidewater glaciers with complex geometries, the glacier centroid may fall in an ocean/no-data grid cell, even though the glacier itself originates on land and is connected to coastal terrain. In these cases, we use the nearest available land grid cell as the meteorological forcing. We do not expect this procedure to introduce a systematic bias, although local representativeness errors may occur in coastal regions, in steep topography or for glaciers much larger than the climate grid resolution. These effects are partly mitigated by downscaling temperature and precipitation over the glacier elevation range using prescribed lapse rates. We added two sentences in the text in **lines 625-629:**

“Because ERA5-Land provides valid values only over land surfaces, coastal glaciers whose centroid falls offshore are assigned the nearest valid land grid cell. We do not expect this procedure to introduce a systematic bias, although local representativeness errors may occur in coastal or steep terrain (see section Mass balance model).”

**Reviewer 2 – Comment 11**	**(R2.11)**
Lines 382–383: The delta-method bias correction over 1991–2020. Does this preserve daily variability statistics (variance, autocorrelation, extreme tails) or only the mean? This is critical given the manuscript's focus on extremes.	

We have clarified that the monthly delta-method was used to ensure a controlled comparison between the daily and monthly model configurations. The aim of the study is to isolate the effect of temporal resolution in the mass-balance model, rather than to assess the impact of different bias-correction methods (see for example Weathers et al. (2025). The delta-method corrects monthly mean biases but does not explicitly adjust daily variability statistics, such as variance, autocorrelation or distribution tails. Applying a more advanced daily bias-correction method only to the daily forcing would introduce an additional methodological difference between the two configurations and could therefore confound the comparison. We added two sentences in the text on this in **lines 665-668**:

“This approach corrects monthly mean biases but does not explicitly adjust daily variability statistics, such as variance, autocorrelation or extreme values. Applying a more advanced daily bias-correction

method only to the daily forcing would introduce an additional methodological difference and could therefore confound the comparison between model configurations."

**Reviewer 2 – Comment 12**	**(R2.12)**
Lines 422–423: The 0.5–2.5 °C snow–rain transition is treated as a fixed range. Recent work (cited as ref. 26, Jennings et al. 2025) suggests this range varies regionally. A brief acknowledgment would be appropriate.	

We agree that the snow–rain transition temperature might not be spatially uniform but can vary regionally depending on local climatic conditions, as highlighted by Jennings et al. (2025). In GloGEM, a fixed linear transition between 0.5°C and 2.5°C is applied globally for consistency between regions and computational efficiency. We have now acknowledged this in the manuscript and note that incorporating regionally varying snow–rain transition thresholds could further improve the representation of precipitation phase in future model developments. We added in **lines 721-724**:

"While recent work has shown that snow-rain transition temperatures can vary regionally depending on local climatic conditions (e.g., Jennings et al., 202526), a fixed transition range is applied here globally for consistency across all glacierised regions."

**Reviewer 2 – Comment 13**	**(R2.13)**
Lines 466–470: "Processes such as basal melt, debris cover and internal accumulation are not included". This omission is more consequential for some regions (e.g., debris cover in High Mountain Asia). Briefly discuss whether this could systematically affect the dailyvs- monthly comparison in those regions.	

We agree that some omitted processes, particularly debris-cover effects, can be regionally important, for example in High Mountain Asia. However, because both the daily and monthly model configurations are calibrated against the same geodetic mass-balance observations (Hugonnet et al., 2021), the integrated effects of processes such as basal melt, internal accumulation, and debris cover are implicitly incorporated into the calibrated parameter sets. While the explicit representation of these processes could improve the physical realism of the simulations, we do not expect their omission to systematically affect the comparison between daily and monthly model resolutions. We have clarified this by adding in **lines 774-779**:

"While some of these processes, particularly debris-cover effects, may be regionally important (e.g., in High Mountain Asia) (Herreid and Pellicciotti, 2020)[82], both model configurations are calibrated against geodetic mass-balance observations[39], which inherently integrate their net effects. As a result, these processes are indirectly reflected in the calibrated parameter values, and their omission is not expected to systematically affect the comparison between daily and monthly model resolutions."

Reviewer 2 – Comment 14	(R2.14)
Lines 530–532: The computational cost statement ("approximately 30 times higher") is a useful figure. Provide the absolute runtime (CPU-hours) for the daily and monthly global runs to help readers calibrate feasibility for their own work.	

We thank the reviewer for this helpful suggestion. The previously stated factor of approximately 30 was a conservative estimate based on theoretical scaling from monthly to daily time steps. Following a more detailed assessment of the current model implementation, including recent improvements in the computational workflow, we find that the additional computational cost is substantially lower. In our present setup, the daily model requires approximately ten times more computation time than the monthly model, indicating that daily simulations are computationally feasible for large-scale applications. We have therefore revised the text to provide both the relative increase in computational cost and an absolute benchmark runtime.

The revised text now reads in **lines 894-900:**

"The simulations were performed on a Linux workstation equipped with two Intel Xeon E5-2687W v4 processors, providing 24 physical CPU cores in total. The model runs used here were executed as single-core jobs, so each individual simulation used one CPU core rather than the full multi-core capacity of the machine. Under this setup, the daily version of the model takes approximately ten times longer than the monthly version. For a test sample of about 1000 glaciers, this corresponds to roughly 1.2 hours of computation time for the daily model."

Reviewer 2 – Comment 15	(R2.15)
Figure 1a: The grey shaded band (±2σ of observations) is helpful but the colour distinction between daily (orange) and monthly (blue) circles is hard to read in the dense scatter. Consider using shape differentiation in addition to colour.	

We considered adding shape differentiation between the daily and monthly model results but in the end decided to keep the current design to avoid further increasing the visual complexity of an already dense scatter plot. We therefore kept Fig. 1a unchanged.

Reviewer 2 – Comment 16	(R2.16)
Figure 1b (elevation-band): The elevation-band RMSE of ~1 m w.e. is much higher than the glacier-wide RMSE (~0.6). Briefly explain to readers why elevation-band errors are inherently larger.	

We have added a short clarification that elevation-band errors are expected to be larger because they evaluate the model's ability to reproduce the vertical distribution of mass balance, rather than the glacier-wide mean. This is a more stringent test, especially because the model is primarily calibrated against glacier-wide geodetic mass changes and not directly against elevation-band gradients. We reformulated this as such in **lines 133-136**:

"Similar improvements are found for mass balances aggregated over elevation bands and for regional mass changes (Fig. 1b-c). The higher RMSE for the elevation-band values is expected, as these data provide a more detailed test of the vertical mass-balance distribution, whereas calibration is primarily constrained by glacier-wide geodetic mass balances (see Methods)."

**Reviewer 2 – Comment 17**	**(R2.17)**
Figure 1c: The Central Europe 2022 highlighted point is effective. Consider adding 1–2 other named extreme years (e.g., Svalbard 2024, Western Canada 2023) to make the extreme-year argument more visually concrete.	

We have added the Western Canada and USA 2023 extreme year to Fig. 1c to make the extreme-year comparison more visually concrete. We also considered highlighting Svalbard 2024, but this point has a regional mass balance of approximately −1 m w.e. and lies near the centre of the scatterplot, making it difficult to distinguish visually. We therefore did not add Svalbard to avoid overcrowding the figure.

**Reviewer 2 – Comment 18**	**(R2.18)**
Lines 142–144: "high-flow glacier runoff in Western Canada and the USA is projected to occur ~46 days earlier by 2100". Please clarify whether this is the daily model under SSP5- 8.5 vs. the 2001–2020 baseline (which I infer but is not explicit).	

We have clarified that the ~46-day advance in high-flow glacier runoff for Western Canada and the USA refers to the daily model under both SSP3-7.0 and SSP5-8.5 by 2100, relative to the 2001–2020 historical baseline. We have also clarified that the Southern Andes show a broader projected shift of ~10–40 days earlier. This now reads in **lines 215-219**:

"By 2100, for example, the daily model projects high-flow glacier runoff in Western Canada and the USA to occur ~46 days earlier under both Shared Socioeconomic Pathway (SSP) 3-7.0 and SSP5-8.5, relative to the 2001–2020 historical baseline (Fig. 2a). In the Southern Andes, high-flow timing shifts by ~10–40 days earlier, depending on the scenario (Fig. 2c)."

**Reviewer 2 – Comment 19**	**(R2.19)**
Figure 2 panel labels: The figure caption refers to panel "f" but I see only panels a–e in the description. Please verify the panel labelling matches the figure.	

We have verified the figure caption and corrected the panel labelling. We removed the reference to 2f.

Reviewer 2 – Comment 20	(R2.20)
Extended Data Fig. 4: The +8% (2022) and –12% (2014) comparison is striking but represents only two years. Provide the full distribution of daily-vs-monthly annual runoff differences for Central Europe over 2000–2024 to support the generality of the claim.	

We agree that the highlighted years represent extreme examples. Our intention was to illustrate the magnitude of the differences that can occur during anomalous years rather than to suggest that such differences are typical. We now report summary statistics for the full 2000–2024 distribution in the manuscript text, which reads in **lines 237-240**:

"Across 2000–2024, the median annual daily-minus-monthly runoff difference is −2%, with a standard deviation of 4.5%. These contrasts show that both runoff magnitude and timing are sensitive to year-to-year climate variability and to an explicit representation of sub-monthly processes, especially during extreme years."

Reviewer 2 – Comment 21	(R2.21)
Lines 171–173: "ice melt accounts for approximately 70–80% of present-day glacier runoff by late summer (September), whereas the monthly model yields a substantially lower contribution of 44%". This is a large difference. Is it possible to validate against in situ HMA hydrological observations (e.g., isotope-based partitioning studies)?	

We agree that an independent validation of the simulated runoff partitioning using in situ hydrological or isotope-based observations would be valuable. However, such a validation is complex and would warrant another, dedicated study. The main challenge is that suitable observations are not available across High Mountain Asia, especially for the specific partitioning between snowmelt, ice melt and other runoff components. Available isotope-based or catchment-scale studies are typically limited to individual basins, short time periods, and locally specific hydrological settings. As such, they are difficult to compare directly with our regional-scale glacier-model results. Furthermore, our model does not resolve runoff contributions from non-glacierized regions, making a comparison to direct sampling data even more difficult. We therefore interpret the reported difference in late-summer ice-melt contribution as a model-based indication of how temporal forcing resolution affects the simulated runoff components, rather than as a directly validated regional estimate. To avoid overinterpretation, we have clarified this point in the manuscript in **lines 312-314**:

"These component-specific runoff estimates are intended as model-based diagnostics of temporal-resolution effects, rather than as directly validated regional partitioning estimates."

**Reviewer 2 – Comment 22**	**(R2.22)**
Figure 3a–c (causes): The schematic panels are useful but could be more clearly linked to the right-hand patterns. Consider adding small icons or coloured borders matching each pattern to the relevant divergence panel (d–g).	

We added a small icon in the left panels which are recurring the right panels:

[editorial note: third party material redacted]

**Reviewer 2 – Comment 23**	**(R2.23)**
Extended Data Table 1: The "Global" row shows -24 ± 9% (SSP1-2.6, daily) vs. -21 ± 8% (monthly). The text reports "26 ± 10% (SSP1-2.6) and 47 ± 17% (SSP5-8.5)". The signs and values differ slightly. Please reconcile (likely a 2025 vs. 2015 reference year issue, but should be made explicit in the table caption).	

Done. We updated the table with the correct numbers.

Reviewer 2 – Comment 24	(R2.24)
Extended Data Table 2: The comparison to Zekollari et al. (2024) is valuable, but the monthly model in this study yields less loss than the previous GloGEM CMIP6 results (e.g., –32 ± 12% vs. –39 ± 21% for SSP3-7.0 by 2100). The text notes this (lines 277–278) but does not explain why, given that both are monthly GloGEM with similar forcing, this discrepancy deserves a sentence or two of discussion. Is it due to ERA5-Land vs. ERA5, the updated calibration, 2015 vs. 2025 baseline, or the >5 km² subsetting?	

We have added a short discussion of why the monthly model projections in this study differ from previous GloGEM CMIP6 results. A direct attribution is difficult because several aspects differ between the studies, but we expect the discrepancy to mainly reflect differences in the selected CMIP6 GCM ensemble and the use of an extended reanalysis period. The latter includes ten additional years up to 2024, which affects the reference state. The updated calibration procedure and the exclusion of glaciers smaller than 5 km² may also contribute to the difference but are unlikely to be the dominant source given the overall consistency of the projections and the fact that we include more than 90% of the global volume. We added this in the text in **lines 438-443**

"The slightly smaller losses projected by our monthly model compared to previous GloGEM CMIP6 results likely reflect differences in the selected GCM ensemble and the use of an extended reanalysis period up to 2024, which affects the reference state. Updated calibration choices and the >5 km² glacier subset may also contribute but are unlikely to dominate as we capture the majority of the ice volumes (see Extended Data Table 1)."

Reviewer 2 – Comment 25	(R2.25)
Figure 4b: The diverging y-axes (volume difference on left, SLR difference on right) are slightly hard to parse. Consider stacking the panels or using a single normalised axis.	

We agree that the use of two y-axes requires some care when interpreting the figure. However, we decided to retain the current layout because the two axes show the same underlying difference expressed in two physically meaningful units: relative glacier-volume difference and the corresponding sea-level-rise equivalent. Stacking the panels would increase the size and complexity of the figure, while a single normalised axis would remove the direct link to these two commonly used quantities.

Reviewer 2 – Comment 26	(R2.26)
Lines 273–275: "the divergence ... does not stabilise by 2100 ... suggesting that differences may continue to grow beyond 2100". This is a strong claim. Either run a short extension (e.g., to 2150) or temper the statement.	

We agree that the original wording implied behaviour beyond the simulation period that is not directly supported by our results. We have therefore revised the sentence to limit the interpretation to the 21st century while retaining the main finding that the divergence does not stabilise by 2100. The text now reads in **lines 485-489**:

"The divergence between daily and monthly projections increases steadily throughout the 21st century and does not stabilise by 2100 (Fig. 4b). Given the strong trend still apparent at the end of the century, and previous studies highlighting the multi-centennial response timescales involved in global glacier evolution[19,52], these temporal-resolution effects are likely to persist beyond the 21st century."

Reviewer 2 – Comment 27	(R2.27)
Lines 287–290: The opening of the discussion is somewhat repetitive of the abstract. Consider restructuring to emphasise the implications rather than re-summarising findings.	

We revised the paragraph to keep a short transition from the results to foreground the implications for large-scale glacier modelling, coupled impact studies and the trade-off between physical realism and computational feasibility. We revised the start of this section which now reads in **lines 461-465**:

"The temporal resolution of glacier models is more than a technical choice: it can shape both projected glacier change and its downstream impacts. This is particularly relevant when glacier projections are used in coupled hydrological, land-surface and impact frameworks, where the timing, variability and extremes of glacier melt can matter as much as long-term changes in glacier volume. Daily glacier models can explicitly represent nonlinear responses associated with temperature thresholds, short-lived meteorological extremes and rapid surface-state transitions, whereas monthly models generally represent these processes in an aggregated way. Our results show that these differences can propagate into runoff seasonality, interannual variability and long-term ice-volume evolution. Temporal resolution should therefore be considered carefully in impact assessments such as studies of (dry-season) water supply[3] [34] or hydropower production in glacier-fed catchments."

Reviewer 2 – Comment 28	(R2.28)
Lines 296–298: "Many coupled applications require temporally consistent runoff estimates and realistic interannual variability, particularly in regions where glacier melt sustains dryseason water supply and hydropower production." It is a strong statement; cite the relevant glacio-hydrological coupling literature explicitly here (refs. 22–24, 32–35 are cited earlier but the link to this conclusion could be tighter).	

We have therefore softened the wording by replacing "sustains" with "contributes to" and added an explicit reference to Van tiel et al (2023). See our response in R2.27 for the textual changes.

Reviewer 2 – Comment 29	(R2.29)
Lines 308–319: This paragraph on pathways for improving monthly models is excellent and should be expanded. Specifically, the suggestion that snowline observations could constrain models (ref. 55 (actually ref. 54)) is intriguing. Could the authors comment on whether their daily model could be calibrated/validated this way in future work?	

We agree that this is an important section and have slightly expanded the paragraph. As noted in our response to R2.2, we additionally performed a sensitivity experiment with a modified monthly accumulation scheme and changing standard deviation. Regarding snowlines, we agree that snowline and snow-cover observations provide a promising avenue for future calibration and validation of the model. This is particularly feasible for the daily version of GloGEM, as it simulates daily snowline evolution that can be directly compared with remotely sensed snow-cover products. In future work, such observations could be used as an additional calibration constraint, for example by minimizing mismatches between observed and simulated snow-covered area or snowline elevation. We have added a sentence to clarify this point and cite recent work using Sentinel-2-derived snow-cover observations for this purpose (Cremona et al., 2025). We also want to mention that we have currently another study in review which is using snowlines as an additional constraint for the calibration of GloGEM, by co-author Alexandra von der Esch. The text now reads in **lines 553-562**:

"In parallel, the growing availability of new observational constraints, such as snowlines or snow cover datasets[56], provides new opportunities to better constrain the spatial extent of snow cover in glacier models. This could help reduce some of the biases present in glacier models, e.g., those linked to model equifinality in which various parameter combinations allow reproducing similar observations[18]. For the daily version of GloGEM, such observations are particularly promising because remotely sensed daily snow-cover patterns can be compared directly with simulated snow conditions and used as additional calibration or validation constraints[57]. By contrast, observations representing specific points in time are more difficult to incorporate consistently in a monthly model framework"

Reviewer 2 – Comment 30	(R2.30)
Lines 572–577: The statement that "The GitHub version is currently undergoing final evaluation, with a public release planned for late spring alongside full documentation" is concerning for a Nature Communications submission, which operates under transparent peer review. Code should be available at the time of review to allow independent assessment. Please provide at minimum a private repository link (or anonymised code archive) to the reviewers and editors during the revision. Simulations output data: Specify the repository (e.g., Zenodo, PANGAEA) and provide a placeholder DOI if available.	

We have made the full code of GloGEM available alongside a full documentation.

The text now reads in **lines 951-955**:

"As part of this study, we have made a fully documented version of GloGEM publicly available on GitHub[84]. This release supports both daily and monthly input configurations and includes online documentation describing the model setup, required input data, and simulation workflow. All simulation outputs associated with this study will be made available in NetCDF format through Zenodo upon acceptance."

With reference to:

Huss, M., Van Tricht, L., Beer, J., and von der Esch, A.: GloGEM: The Global Glacier Evolution Model (Version 0.1.1) [Computer software]. https://doi.org/10.5281/zenodo.21133140, 2026

Reviewer 2 – Comment 31	(R2.31)
Reference 51 (Screen, 2014): This is cited at line 275 ("does not stabilise by 2100, suggesting that differences may continue to grow") but ref. 51 is about Arctic amplification reducing temperature variance, the connection is unclear. Verify the citation.	

We modified this to now correctly refer to

Schuster, L., Maussion, F., Rounce, D. R., Ultee, L., Schmitt, P., Lacroix, F., Frölicher, T. L., and Schleussner, C.-F.: Irreversible glacier change and trough water for centuries after overshooting 1.5 °C, Nat. Clim. Chang., 15, 634–641, https://doi.org/10.1038/s41558-025-02318-w, 2025.

Reviewer 2 – Comment 32	(R2.32)
Reference numbering inconsistency: Ref. 47 (Aguayo et al. 2024 on Patagonia uncertainty) appears in the reference list but I could not locate its in-text citation. Please verify. The in-text numbering skips	

We now refer to this study in **lines 425-438**:

"While a direct comparison with existing global-scale glacier projections from the Climate Model Intercomparison Project version 6 (CMIP6) is complicated due to differences in the choice of the reanalysis product[49], future forcing (i.e., a different CMIP6 global climate model (GCM) ensemble considered), model setup, and exclusion of glaciers smaller than 5 km², there is generally a good agreement between the values from the literature[13,14] and our monthly model counterparts (Extended Data Fig. 11)."

**Reviewer 2 – Comment 33**	**(R2.33)**
Reference 47 entirely, jumping directly from citation 46 (line 238) to citation 48 (line 257).	

We now refer to reference 47 in line 394.

**Reviewer 2 – Comment 34**	**(R2.34)**
Line 102: Footnote-style reference to "the daily model[1]". ref. 1 is the Glambie Team paper, which is the observational dataset. The phrasing is potentially confusing; consider rephrasing as "compared to Glambie observations[1]."	

We added "*compared to GlaMBIE observations*."

**Reviewer 2 – Comment 35**	**(R2.35)**
Line 317: Reference 55 is actually a paper on coastal flooding by Nicholls et al., while Reference 54 is the correct paper on transient glacier snowlines by Loibl et al.	

Corrected.

**Reviewer 2 – Comment 36**	**(R2.36)**
Several Extended Data figures (e.g., ED Fig. 2 mass-balance gradients) could benefit from including the sample size (number of glaciers) per region directly on the plot.	

To not overload the figures, we decided to not add the number of glaciers per region but we added this information in Extended Data Table 1.

**Reviewer 2 – Comment 37**	**(R2.37)**
Extended Data Fig. 7 is a useful comparison but the labelled outliers (Svalbard and J.M., Russian Arctic in SSP1-2.6 2100 panel) are not discussed in the caption. A brief note on what drives these outliers would help readers.	

We added in the caption:

"Labelled regions indicate where differences between the previous projections and the new estimates are largest."

Reviewer 2 – Comment 33	(R2.33)
Line 30: "an additional 20-33 mm to global mean sea-level rise": consistently use either en-dashes or hyphens in numerical ranges throughout (multiple inconsistencies exist).	

Done.

Reviewer 2 – Comment 34	(R2.34)
Line 53: "limited number of large-scale glacier evolution models have been forced with daily data", here "small number" reads more naturally than "limited number."	

Modified as suggested.

Reviewer 2 – Comment 35	(R2.35)
Line 165: "snouth" should be "snout" (line 551 in Methods).	

Done.

Reviewer 2 – Comment 36	(R2.36)
Lines 215–216: "relative losses that are typically 2–5% higher in the daily model". Clarify whether this is the relative difference in remaining volume, or the absolute percentagepoint difference in projected loss.	

We have clarified that the reported 2–5% refers to an absolute percentage-point difference in projected ice-volume loss relative to the 2025 glacier volume, calculated as the daily minus monthly modelled loss. We modified this in the text which now reads in **lines 346-351**:

"While these effects accumulate over time, the differences in modelled ice volume remain generally moderate: by the end of the century and compared with the monthly model, the daily model typically projects additional ice-volume losses of 2–5% relative to the 2025 glacier volume (Fig. 3e). New Zealand also exhibits enhanced ice loss in the daily model, although this does not appear to be associated with a systematically steeper mass-balance gradient (Extended Data Fig. 3)."

Reviewer 3

Reviewer 3 – Comment 1	(R3.1)
Abstract: For clarity, I would say something like "Current global glacier models typically use temperature-index mass balance calculations forced with temporally aggregated meteorological data". This model cannot resolve surface-albedo feedbacks at any time scale. I think that what is intended is that daily resolution allows more rapid transitions between snow, firn and ice degree-day factors.<br><br>It should be noted that both approaches, which differ in 2025-2100 projections, are calibrated against 2000-2019 mass balances.	

We thank the reviewer for this useful suggestion. We have revised the opening sentence which now reads in **lines 18-20:**

"Current global glacier models typically rely on temperature-index mass balance calculations forced with temporally aggregated meteorological data, limiting their ability to resolve responses to short-term meteorological variability."

Both the daily and monthly model configurations are calibrated against the same 2000–2019 geodetic mass-balance observations before being used for the 2025–2100 projections, meaning only temporal resolution differs while the other components of the modelling setup are consistent. We now explicitly added the information on the calibration data and time frame in **lines 783-785**.

"Model calibration ensures that the modelled glacier-wide mass balance reproduces the glacier-specific geodetic mass-balance estimates of Hugonnet et al. (2021)[39] for 2000–2019 within ca. ±0.1 m w.e. yr^{-1}."

Reviewer 3 – Comment 2	(R3.2)
Improved mass balance representation: An estimate should be given for the uncertainty in WGMS mass balances. I believe that this will have 0.1 m w.e. order of magnitude, making the 0.02 m w.e. improvements in RMSE and MAE look very marginal. The improvements for extreme years are more significant and consistent with expectations for increasing temporal resolution.	

We agree that the overall improvements in RMSE and MAE are modest compared to the uncertainty of individual WGMS observations. We therefore revised the text to emphasise that the strongest evidence for the added value of daily forcing comes from the substantially improved representation of extreme mass-balance years. In **lines 109-115**, we now write:

"In general, the daily model shows a slight improvement over the monthly model with the root mean squared error (RMSE) decreasing from 0.63 to 0.61 m w.e., the mean absolute error (MAE) decreasing from 0.48 to 0.46 m w.e., and the coefficient of determination (R^2) increasing from 0.71 to 0.75 (Fig. 1a). These skill scores also represent an improvement on earlier evaluations of GloGEM[16] (RMSE: 0.73

m w.e), which were based on fewer glaciers, shorter records, and lacked glacier-specific calibration data[14].”

We agree that uncertainties in WGMS mass-balance estimates should be explicitly stated here. Although these uncertainties are not straightforward to quantify in a fully consistent manner across programmes and glaciers, the reported annual glacier-wide mass-balance uncertainty is typically on the order of a few hundred millimetres water equivalent per year (approximately ±0.2–0.4 m w.e. yr^{-1}). This range varies between glaciers and monitoring programmes, depending on factors such as stake density, the representation of accumulation measurements, spatial extrapolation procedures, and geodetic validation constraints. We have now clarified this in **lines 123–131** and more explicitly highlight the added value of the daily model, particularly in capturing more extreme years and short-term variability that may not be fully resolved within the observational uncertainty range.

“Given that uncertainties in annual WGMS mass-balance observations[38] are typically of the order of ~0.2-0.4 m w.e. yr^{-1}, the improvement in overall RMSE and MAE should be regarded as modest. The added value of the daily model becomes more apparent in extreme years. For mass balance anomalies exceeding the mean by ±2σ, the RMSE for the glacier model decreases from 0.98 to 0.83 m w.e. and the MAE decreases from 0.81 to 0.64 m w.e., when moving from the monthly to the daily model. This indicates a substantially improved representation of both strongly negative and strongly positive mass balance years, driven by enhanced sensitivity to short-term meteorological extremes, and steeper mass balance gradients (Extended Data Fig. 3). “

Reviewer 3 – Comment 3	(R3.3)
Glacier runoff : The authors could consider substituting Extended Data Figure 5 for the map in Figure 2. Would a spline fit to the monthly model runoff be very different to the daily model?	

We agree that the seasonal evolution of runoff components is more informative than the map previously shown in Fig. 2, and we therefore replaced the map with the former Extended Data Fig. 5. Regarding the spline fit, we agree that smoothing the monthly runoff curve would make it visually more similar to the daily curve. However, such a spline would only interpolate between monthly values and would not recover the missing sub-monthly variability, and the timing of short-lived melt and runoff events. We therefore decided to retain the monthly runoff as stepped monthly values. The updated figure is shown below:

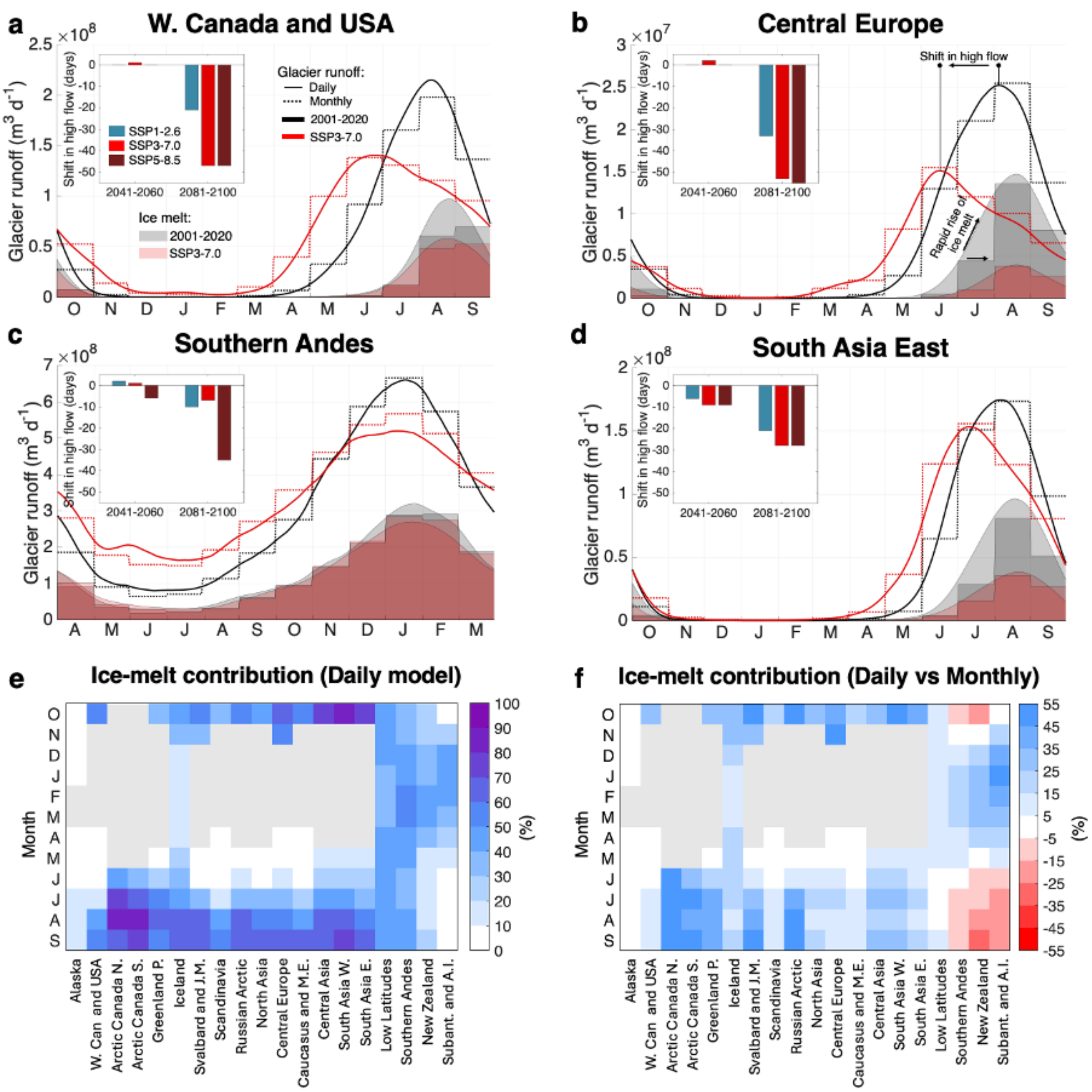

Fig. 2: Evolution and variability of glacier runoff as unravelled through daily modelling. a, b, c, d Modelled mean glacier runoff at daily (solid lines) and monthly (dashed lines) temporal resolutions for four climatologically distinct regions (panels) (other regions in Extended Data Fig 3). Shaded areas indicate the ice-melt component of runoff at present-day (grey, 2001–2020) and 2081–2100 under SSP3-7.0 (red). Insets at top left show shifts in the timing of the seasonal peak (in days) for the daily model compared to the 2001–2020 baseline. e Seasonal cycle of the ice-melt contribution (%) as modelled by the daily model across all RGI regions. Months for which the runoff is <1% of the maximal monthly runoff are greyed out to avoid showing excessively large ice-melt contributions for periods with minimal glacier runoff. f The difference in contribution of ice-melt compared to the monthly model (%).

Reviewer 3 – Comment 4	(R3.4)
Projected ice volume: There is very large equifinality in calibrating precipitation correction factor, degree-day factor and temperature bias to 2000-2019 geodetic mass balances, giving different temperature sensitivities for equally good fits (Schuster et al. 2023, cited as 36). Claims about impacts of forcing frequency on projections would be more robust with an estimate of the resulting uncertainty.	

We agree that calibrating several parameters against one geodetic mass-balance estimate can lead to parameter equifinality, and that different combinations of precipitation correction, degree-day factors and temperature bias may produce similarly good fits over the calibration period but different sensitivities in future projections. While a full parameter-uncertainty assessment would require a dedicated ensemble calibration experiment, we now added an additional analysis to test the robustness of our results to the calibration strategy.

In the analysis we now compare the projections obtained with our cross-calibrated approach, in which Cprec and Tbias are averaged between the daily and monthly configurations, with projections based on independent calibrations, where these parameters are individually adjusted for each of the two temporal resolutions. This comparison is shown in Extended Data Fig. 9 (see also bottom of this document). The results show that, although the absolute projected volume changes differ somewhat between calibration strategies, the sign and regional pattern of the daily–monthly differences remain consistent. This suggests that the main conclusion of the study, namely that temporal forcing resolution affects projected glacier evolution, is not directly related to the calibration procedure but rather the temporal scales considered. We have added this comparison and reworded the manuscript to make clear that this analysis provides a robustness check rather than a full quantification of calibration-parameter uncertainty.

We added in **lines 417-423**:

"We further tested the sensitivity of the volume projections to the calibration design by repeating the projections with an alternative calibration setup (the original, independent setup, in which C_{prec}, T_{bias} and the DDFs are calibrated separately for the daily and monthly model, see Methods). This experiment yields similar, or slightly smaller, daily–monthly differences, while the regional 21st-century volume changes remain broadly consistent (Extended Data Fig. 10). This indicates that the main regional patterns described above are robust to the choice of calibration strategy."

Reviewer 3 – Comment 5	(R3.5)
29 "contributes an additional"	

Modified.

**Reviewer 3 – Comment 6**	**(R3.6)**
96 “mass balances aggregated over elevation bands”	

Modified.

**Reviewer 3 – Comment 7**	**(R3.7)**
184 “greyed out to avoid”	

Corrected.

**Reviewer 3 – Comment 8**	**(R3.8)**
220 “low-relief, ice cap dominated regions”	

Done.

**Reviewer 3 – Comment 9**	**(R3.9)**
324 “more glacier mass loss than”	

Modified.

**Reviewer 3 – Comment 10**	**(R3.10)**
351 Citations such as “Ice thickness distributions are taken from^63” are inelegant. “Ice thickness distributions are taken from Farinotti et al. (2017)^63” would be better.	

Done.

**Reviewer 3 – Comment 11**	**(R3.11)**
404 “compare regional mass changes with independent regional estimates”	

Modified.

Reviewer 3 – Comment 11	(R3.11)
The formatting of equation 1 should be improved.	

Done.

Reviewer 3 – Comment 12	(R3.12)
541 “assumed to be negligible”	

Modified.

Reviewer 3 – Comment 13	(R3.13)
551 “the glacier snout”	

Corrected.

Reviewer 3 – Comment 14	(R3.14)
Code and data not yet available	

We have made the full code of GloGEM available alongside a full documentation.

The text now reads in **lines 951-955**:

“As part of this study, we have made a fully documented version of GloGEM publicly available on GitHub[84]. This release supports both daily and monthly input configurations and includes online documentation describing the model setup, required input data, and simulation workflow. All simulation outputs associated with this study will be made available in NetCDF format through Zenodo upon acceptance.”

With reference to:

Huss, M., Van Tricht, L., Beer, J., and von der Esch, A.: GloGEM: The Global Glacier Evolution Model (Version 0.1.1) [Computer software]. https://doi.org/10.5281/zenodo.21133140, 2026

New and updated tables and figures

Extended Data Table 1: Overview of modelled glaciers by region. *Number of modelled glaciers, glacierised area and total glacier volume included in the simulations for each region. Percentages indicate the relative contribution of the regional total.*

Region	Modelled glaciers		Glacier volume (km^3)		Glacier area (km^2)	
**Alaska**	1296	18 057	95%	5%	71 192	82%
**W. Canada and USA**	468	718	68%	2%	6 045	41%
**Arctic Canada N.**	1402	26 235	93%	31%	99 459	95%
**Arctic Canada S.**	1094	7 789	90%	15%	34 937	85%
**Greenland P.**	2385	13 258	99%	12%	76 937	86%
**Iceland**	99	3 746	99%	17%	10 646	96%
**Svalbard and J.M.**	512	7 324	98%	32%	32 245	95%
**Scandinavia**	137	212	71%	4%	1 330	45%
**Russian Arctic**	606	14 604	99%	57%	50 440	98%
**North Asia**	73	74	53%	1%	742	31%
**Central Europe**	123	81	62%	3%	904	43%
**Caucasus and M.E.**	53	37	62%	3%	489	37%
**Central Asia**	1465	2 282	70%	3%	22 984	47%
**South Asia W.**	911	2 340	82%	3%	20 111	60%
**South Asia E.**	532	595	68%	4%	7 085	48%
**Low Latitudes**	89	38	38%	3%	637	27%
**Southern Andes**	622	5 062	95%	4%	21 916	75%
**New Zealand**	30	50	71%	1%	441	38%
**Subant. and A.I.**	656	47 925	99%	24%	128 580	97%
**Global**	12 571	148 075	94%	6%	583 189	83%

Extended Data Table 2: Evaluation of modelled mass balances against WGMS observations. *Regional evaluation of modelled mean specific mass balances compared with data from the WGMS[37]. Only glaciers with at least three years of observations are included. Regions for which this condition is only met for one or less glaciers are shaded in light orange. RMSE Da. and RMSE Mo. stand for the RMSE of the individually calibrated daily and monthly model.*

Region	RMSE Da.	RMSE Mo.	Nr. Glaciers	Nr. Years	Nr. Observations
**Alaska**	0.59	0.56	8	45	207
**W. Canada and USA**	0.48	0.47	21	45	410
**Arctic Canada N.**	0.17	0.24	3	45	132
**Arctic Canada S.**			0	0	0
**Greenland P.**	0.60	0.62	6	40	74
**Iceland**	0.54	0.70	11	38	302
**Svalbard and J.M.**	0.21	0.53	14	45	270
**Scandinavia**	0.45	0.56	25	45	682
**Russian Arctic**	0.44	0.41	1	5	5
**North Asia**	0.50	0.49	4	39	91
**Central Europe**	0.46	0.70	35	45	1009
**Caucasus and M.E.**	0.21	0.22	2	45	86
**Central Asia**	0.16	0.20	16	45	284
**South Asia W.**	0.52	0.54	6	34	58
**South Asia E.**	0.36	0.32	10	38	102
**Low Latitudes**	1.73	1.76	4	34	67
**Southern Andes**	0.39	0.46	4	44	56
**New Zealand**	0.70	0.62	1	20	20
**Subant. and A.I.**	0.50	0.69	4	26	77

Extended Data Table 5: Regional calibration-parameter statistics. *Mean and standard deviation of all calibration parameters for each region. The upper part of the table reports parameters from the original calibration strategy, in which the precipitation correction factor (C_{prec}), temperature bias (T_{bias}), and degree-day factors of snow (DDF_{snow}) are calibrated independently for the daily and monthly model configurations. The lower part reports the cross-calibrated parameters obtained after averaging C_{prec} and T_{bias} between the daily and monthly configurations for each glacier.*

	C_{prec} (-)				T_{bias} (°C)				DDF_{snow} (mm day^{-1} °C^{-1})			
**Original calibration**	**Daily**		**Monthly**		**Daily**		**Monthly**		**Daily**		**Monthly**	
	Mean	Std	Mean	Std	Mean	Std	Mean	Std	**Mean**	**Std**	**Mean**	**Std**
**Alaska**	1.05	0.43	1.08	0.45	0.26	1.01	0.20	0.93	3.44	0.61	3.36	0.58
**W. Canada and USA**	1.21	0.43	1.29	0.49	0.00	0.02	0.00	0.00	3.08	0.24	3.04	0.19
**Arctic Canada N.**	1.01	0.47	1.02	0.48	0.55	0.86	0.44	0.83	3.77	0.71	3.72	0.72
**Arctic Canada S.**	0.93	0.35	0.97	0.40	0.60	0.82	0.39	0.65	3.87	0.65	3.78	0.67
**Greenland P.**	1.05	0.54	1.07	0.56	1.86	2.17	1.50	2.01	3.85	0.84	3.80	0.85
**Iceland**	0.84	0.13	0.84	0.13	0.29	0.43	0.33	0.46	3.99	0.56	4.02	0.53
**Svalbard and J.M.**	0.97	0.45	1.22	0.60	0.51	1.31	-0.19	1.40	3.96	0.76	3.25	0.70
**Scandinavia**	1.16	0.27	1.22	0.28	0.00	0.00	0.00	0.02	3.01	0.05	3.00	0.02
**Russian Arctic**	0.87	0.24	1.43	0.66	0.62	0.62	-0.05	0.41	4.05	0.66	3.10	0.66
**North Asia**	1.49	0.53	1.58	0.53	0.03	0.15	-0.01	0.13	3.09	0.41	3.03	0.28
**Central Europe**	0.97	0.27	1.00	0.29	0.06	0.33	0.05	0.30	3.28	0.44	3.23	0.41
**Caucasus and M.E.**	1.79	0.68	1.79	0.68	-0.02	0.49	-0.03	0.48	3.11	0.58	3.10	0.55
**Central Asia**	0.95	0.35	0.96	0.35	0.88	1.30	0.73	1.21	3.75	0.69	3.71	0.69
**South Asia W.**	0.90	0.30	0.90	0.30	2.05	2.09	1.90	2.13	3.97	0.63	3.92	0.65
**South Asia E.**	0.96	0.38	0.95	0.36	1.02	1.25	1.01	1.24	3.82	0.70	3.83	0.69
**Low Latitudes**	1.00	0.49	1.01	0.50	0.56	0.97	0.61	1.00	3.87	0.80	3.87	0.81
**Southern Andes**	0.90	0.25	0.90	0.25	0.99	1.15	0.97	1.15	3.90	0.65	3.90	0.65
**New Zealand**	1.67	0.60	1.48	0.53	0.00	0.00	0.00	0.00	2.94	0.19	2.99	0.12
**Subant. and A.I.**	0.91	0.35	1.01	0.48	2.70	3.91	1.93	3.82	4.12	0.71	3.91	0.80
**Cross-calibration**	**Daily**		**Monthly**		**Daily**		**Monthly**		**Daily**		**Monthly**	
	Mean	Std	Mean	Std	Mean	Std	Mean	Std	**Mean**	**Std**	**Mean**	**Std**
**Alaska**	1.06	0.44	1.06	0.44	0.23	0.97	1.06	0.44	3.52	0.68	3.32	0.55
**W. Canada and USA**	1.25	0.46	1.25	0.46	0.00	0.01	1.25	0.46	3.16	0.23	3.01	0.21
**Arctic Canada N.**	1.01	0.46	1.01	0.46	0.49	0.82	1.01	0.46	4.01	1.08	3.72	0.76
**Arctic Canada S.**	0.95	0.37	0.95	0.37	0.50	0.73	0.95	0.37	4.14	0.88	3.67	0.63
**Greenland P.**	1.06	0.54	1.06	0.54	1.68	2.08	1.06	0.54	4.39	1.68	3.70	0.86
**Iceland**	0.84	0.13	0.84	0.13	0.31	0.44	0.84	0.13	3.98	0.57	4.06	0.58
**Svalbard and J.M.**	1.09	0.49	1.09	0.49	0.16	1.29	1.09	0.49	5.55	1.57	2.72	0.75
**Scandinavia**	1.19	0.28	1.19	0.28	0.00	0.00	1.19	0.28	3.06	0.07	2.99	0.04
**Russian Arctic**	1.15	0.40	1.15	0.40	0.28	0.42	1.15	0.40	7.27	2.04	2.33	0.80
**North Asia**	1.53	0.51	1.53	0.51	0.01	0.12	1.53	0.51	3.24	0.94	3.00	0.30
**Central Europe**	0.97	0.27	0.97	0.27	0.06	0.33	0.97	0.27	3.28	0.45	3.21	0.41
**Caucasus and M.E.**	1.79	0.68	1.79	0.68	-0.03	0.49	1.79	0.68	3.12	0.59	3.10	0.57
**Central Asia**	0.96	0.35	0.96	0.35	0.80	1.24	0.96	0.35	4.08	1.22	3.70	0.69
**South Asia W.**	0.90	0.30	0.90	0.30	1.97	2.10	0.90	0.30	4.22	0.97	3.90	0.66
**South Asia E.**	0.95	0.37	0.95	0.37	1.01	1.24	0.95	0.37	3.84	0.73	3.84	0.70
**Low Latitudes**	1.01	0.50	1.01	0.50	0.58	0.98	1.01	0.50	3.86	0.78	4.02	0.94
**Southern Andes**	0.90	0.25	0.90	0.25	0.98	1.15	0.90	0.25	3.96	0.75	3.91	0.66
**New Zealand**	1.57	0.56	1.57	0.56	0.00	0.00	1.57	0.56	2.81	0.18	3.14	0.15
**Subant. and A.I.**	0.96	0.39	0.96	0.39	2.32	3.82	0.96	0.39	10.71	12.56	3.36	1.25

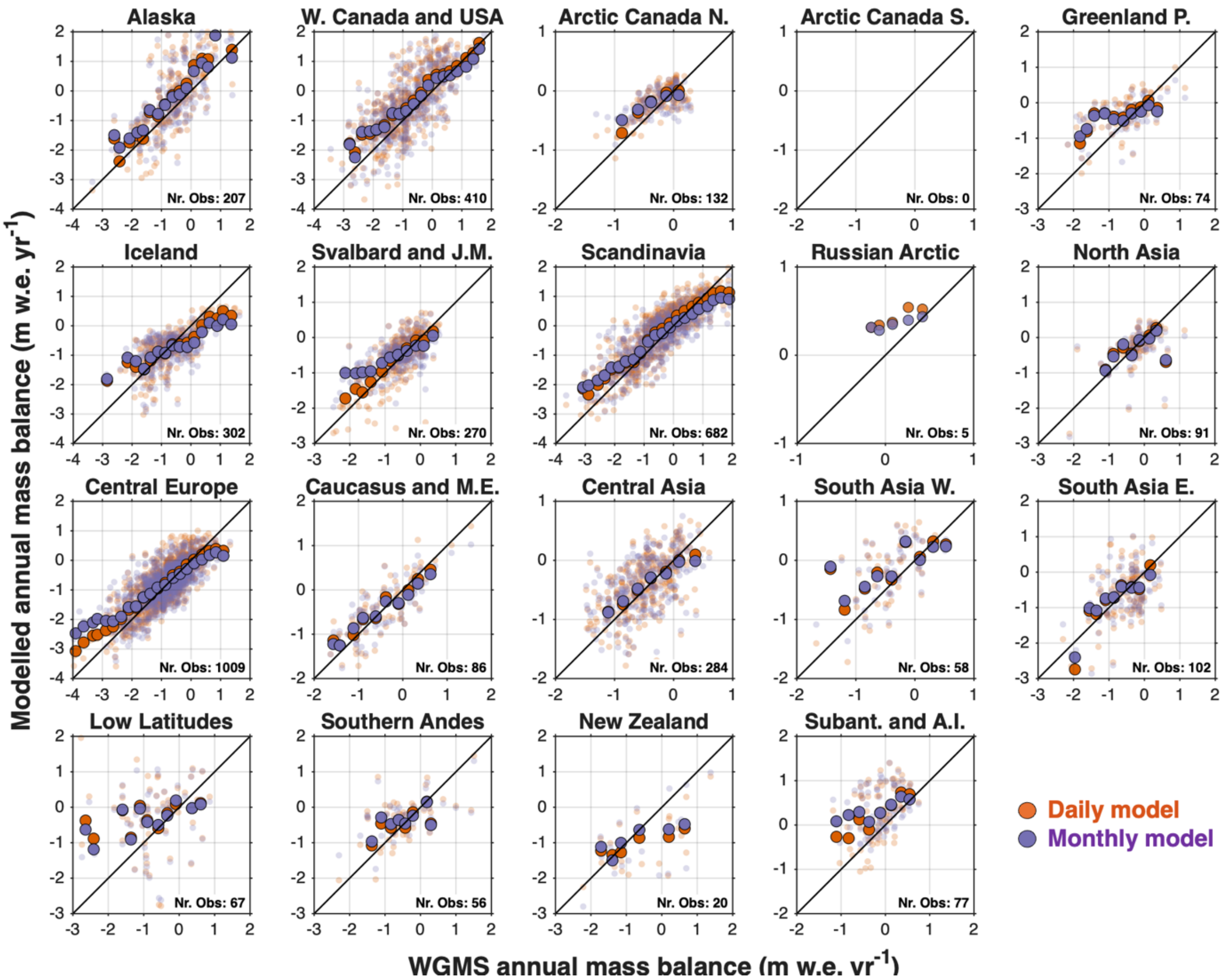

Extended Data Fig. 2: Comparison of modelled and observed glacier mass balance for the daily and monthly models. *Modelled versus observed glacier-wide annual specific mass balance according to WGMS (2025) for the period 1980–2024, shown separately for each RGI region. Only glaciers with at least three years of observations are included. Large circles indicate mean values within 0.5 m w.e. bins, which helps highlight the underlying signal where sufficient data are available. For the Russian Arctic, where the number of points is too small for meaningful binning, larger circles instead show the available individual comparisons. The number of model–observation comparisons (Nr. Obs) is indicated at the bottom right of each panel.*

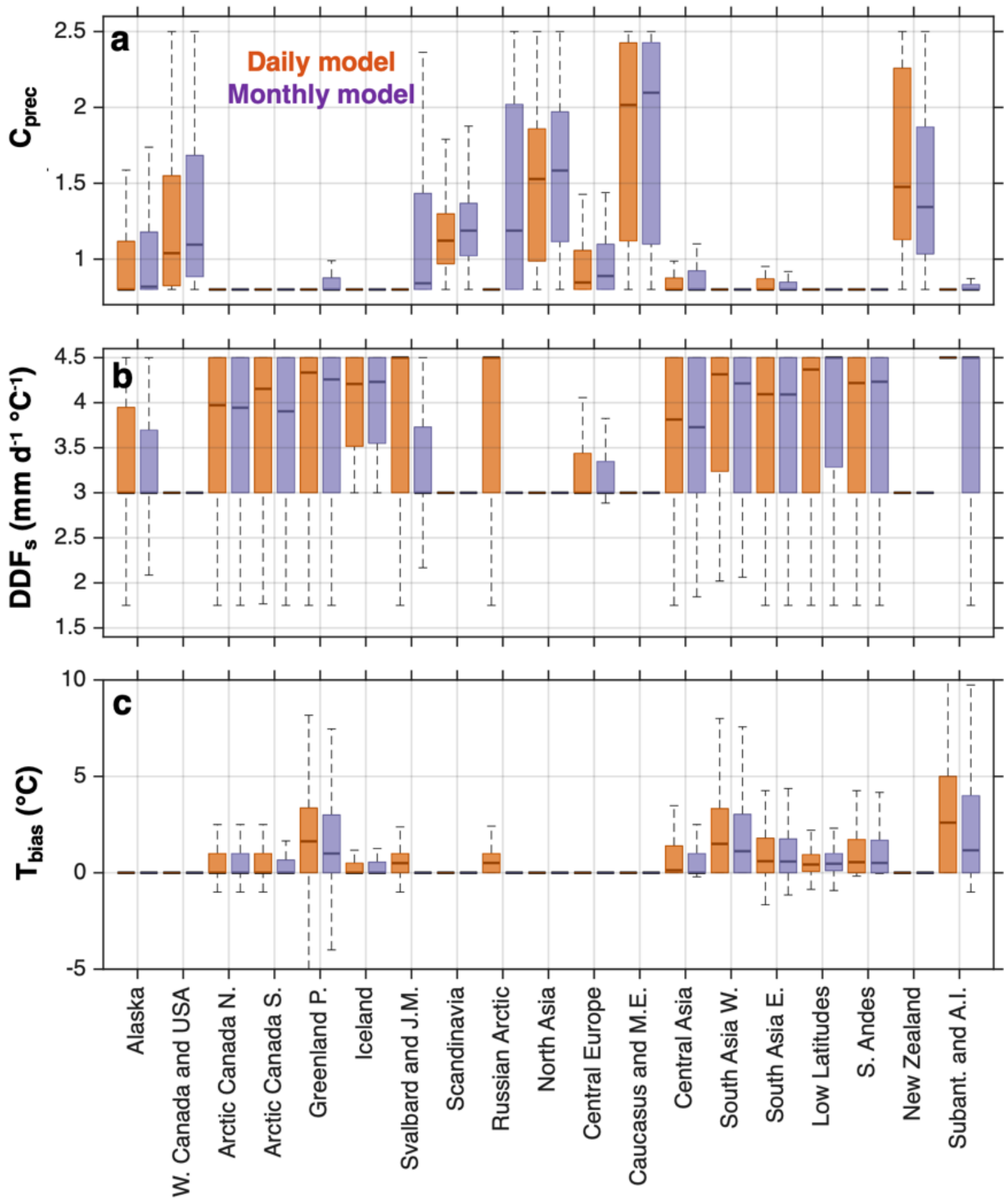

Extended Data Fig. 4: Regional distributions of calibrated model parameters for the daily and monthly model under the original calibration approach. a-c *Distributions of the calibrated precipitation correction factor (C_{prec}), snow degree-day factor (DDF_s), and temperature bias (T_{bias}) obtained from the original calibration procedure. Daily and monthly model calibrations are shown in orange and purple, respectively. Boxplots summarise the distribution of glacier-specific calibration parameters within each RGI region.*

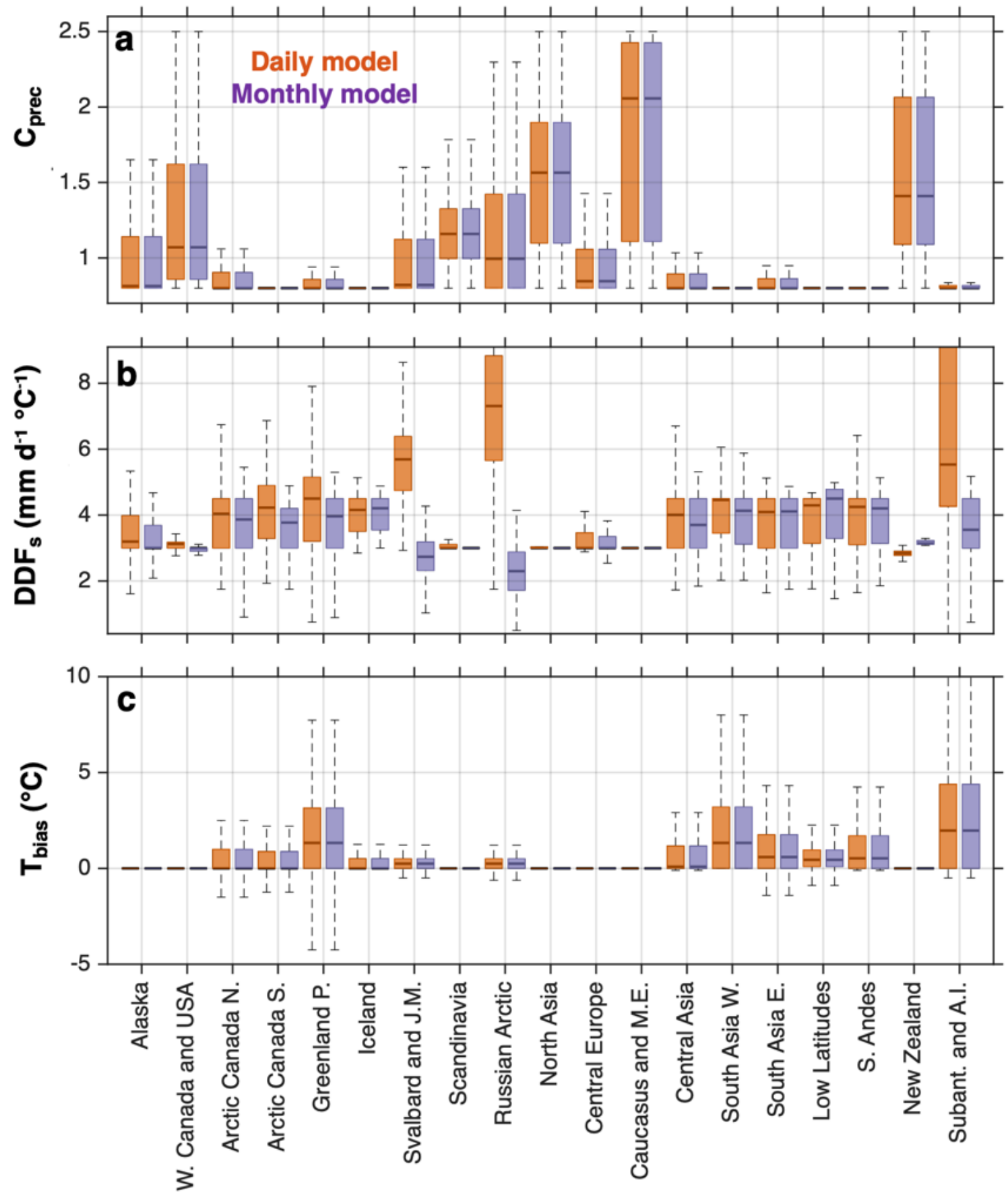

Extended Data Fig. 5: Regional distributions of calibrated model parameters for the daily and monthly model with the cross-calibration approach. a-c *Distributions of the calibrated precipitation correction factor (C_{prec}), snow degree-day factor (DDFs), and temperature bias (T_{bias}) obtained from the cross-calibration procedure. Daily and monthly model calibrations are shown in orange and purple, respectively. Boxplots summarise the distribution of glacier-specific calibration parameters within each RGI region.*

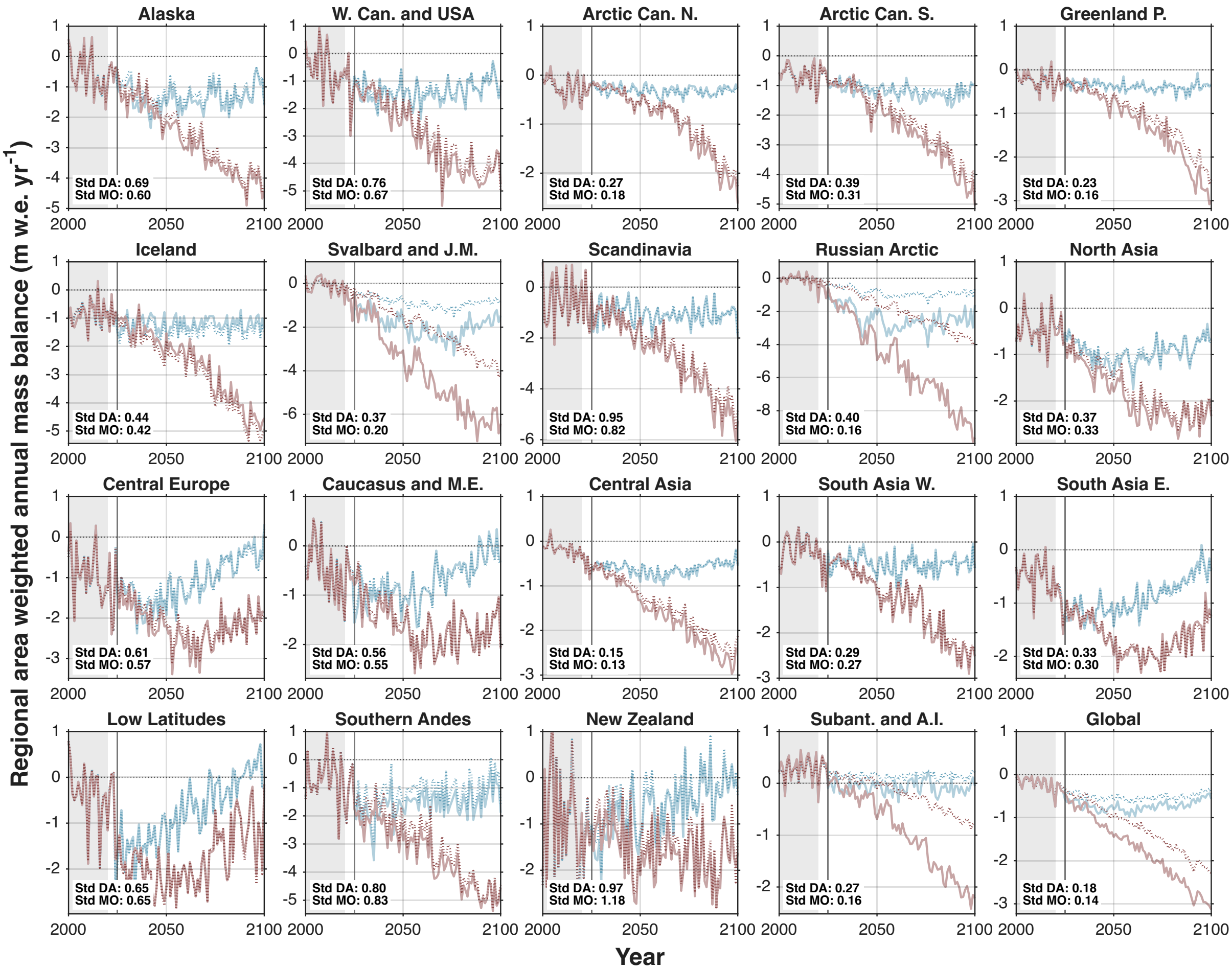

Extended Data Fig. 8: Regional area-weighted mass balance. *Evolution of the regional area-weighted glacier mass balance over 2025–2100 under SSP1-2.6 (blue) and SSP5-8.5 (red). Lines show the multi-GCM mean across five GCMs for the daily model (solid lines) and monthly model (dashed lines). The standard deviation of the daily and monthly modelled mass balance over the 2000–2024 period is shown in the lower-left corner of each panel.*

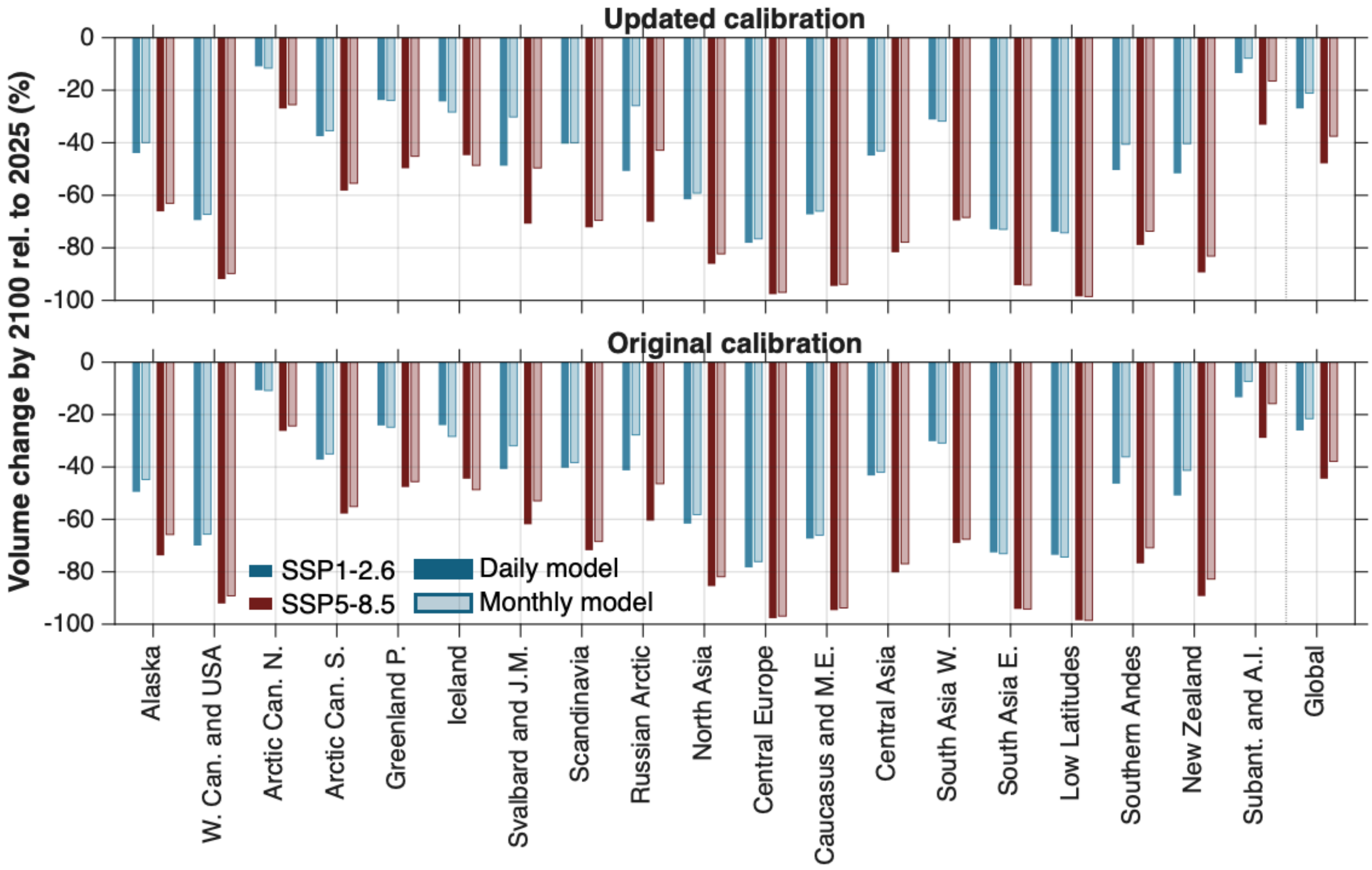

Extended Data Fig. 10: Regional ice volume by 2100 relative to 2025. *Projected glacier volume in 2100 relative to 2025 for the daily and monthly models under SSP1-2.6 and SSP5-8.5. Results are shown for the updated calibration used as the standard in this study, in which* T_{bias} *and* C_{prec} *are cross-calibrated, and for the original calibration, in which all three calibration parameters are calibrated independently for the daily and monthly glacier models. Values indicate multi-GCM mean relative volume change for each region.*

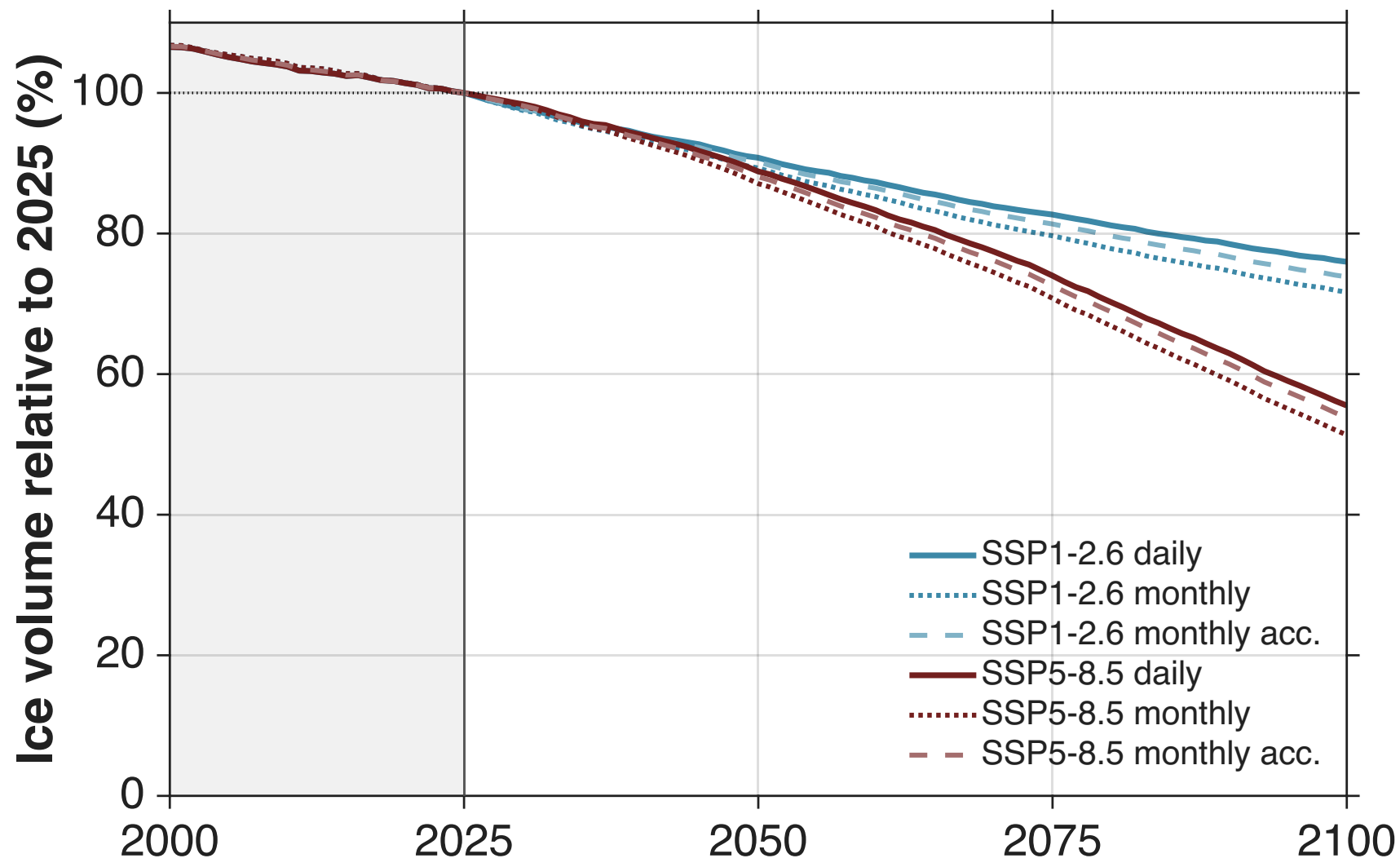

Extended Data Fig. 12: Regional ice volume relative to 2025 in Iceland. *Projected glacier volume relative to 2025 for the daily and monthly models under SSP1-2.6 and SSP5-8.5 in Iceland. "Monthly acc" refers to the monthly model experiment in which accumulation is determined from the daily temperature evolution instead of the monthly mean temperature.*

Resolving short-term meteorological variability amplifies projected glacier loss and sea-level rise

Response to reviewers

Dear reviewers,

Thank you for your constructive comments on our revised manuscript. We are pleased that the revision was positively received and that only a limited number of minor points remain to be addressed. In this response document, we address each of the remaining comments individually. Our replies are highlighted in blue, while all corresponding changes in the revised manuscript are marked in green. We believe that these final revisions have further strengthened the manuscript and have addressed the remaining points raised by Reviewer 2.

Sincerely,
Lander Van Tricht, on behalf of all co-authors

Reviewer 1

General comment	(R1)
I think the manuscript has been substantially improved. The Methods section is now much clearer. I also pointed out several minor issues in the Results section, and I appreciate the authors' careful revisions in response to those comments.	

We are pleased that the revised manuscript satisfactorily addresses the previous comments. We appreciate the reviewer's careful evaluation of the revised version and their positive assessment of the improvements made to the Methods section and the revisions in the Results section.

Reviewer 2

General comment	(R2)
I thank the authors for a careful and constructive response. The revision addresses the substance of all four of my major concerns. The independent-calibration sensitivity experiment (Extended Data Fig. 10), the regional calibration-parameter statistics (Extended Data Table 5; Extended Data Figs. 4–5), the regional validation table and figure (Extended Data Table 2; Extended Data Fig. 2), the Iceland accumulation experiment (Extended Data Fig. 12), the removal of the flood-exposure extrapolation, and the public, documented, DOI-referenced release of GloGEM together make this a materially stronger and more transparent paper. I particularly welcome the added honesty about non-uniform regional performance, which is the kind of statement that makes a modelling paper trustworthy. Some points remain. All are textual, none requires new simulations, and the first is the only one that touches the central claim. I recommend MINOR revision.	

We thank the reviewer for this positive assessment of the revised manuscript and for acknowledging the improvements made in response to the previous round of comments. We are pleased that the additional sensitivity experiments, validation material, calibration diagnostics, and the public release of GloGEM have helped to make the manuscript stronger and more transparent. We also thank the reviewer for the remaining minor comments. We address each of these points in detail below and have revised the manuscript where appropriate to further clarify the text.

**Reviewer 2 – Comment 1**	**(R2.1)**
Framing of the abstract (lines 27–29, 33–35). The abstract states that the relevant mechanisms "cannot be resolved at a monthly time scale" and that resolving high-frequency variability "is essential" for robust projections. The revised discussion is more measured: the Iceland experiment (Extended Data Fig. 12) shows that a monthly model with daily-informed accumulation moves close to the daily model, and the authors conclude at lines 549–551 that simple corrections could improve monthly projections. I find the latter framing both more accurate and stronger result, since it diagnoses the mechanism rather than only reporting the difference. Please bring the abstract into line with it, and refer to the standard monthly GloGEM configuration rather than to monthly models in general. The title may warrant the same consideration.	

We thank the reviewer for this helpful comment. We agree that the original abstract was still phrased too broadly with respect to monthly models in general, whereas our results specifically refer to the standard monthly GloGEM configuration. We have therefore revised the abstract to better align it with the more nuanced discussion and the Iceland accumulation experiment.

In particular, we now avoid stating that the relevant mechanisms cannot be resolved at a monthly time scale in general, and instead clarify that they are not fully represented in the standard monthly GloGEM configuration. The text on lines 27-29 now reads:

"These differences arise from extreme melt days, precipitation-phase transitions, and surface-type feedbacks that are not fully represented in the standard monthly GloGEM configuration."

We also revised the final sentence of the abstract to acknowledge that high-frequency variability may either be explicitly resolved (daily model) or accounted for through suitable parameterisations like the one we tested for Iceland. The text on lines 33-36 now reads:

"Our results show that resolving high-frequency variability in meteorological forcing, or accounting for its effects through suitable parameterisations, is essential for robust glacier change projections, and that its omission may lead to systematic underestimation of future glacier loss and associated impacts."

We considered the reviewer's suggestion regarding the title, but decided to retain it unchanged, as we feel it remains appropriate while the revised abstract now more precisely defines the scope of the comparison.

**Reviewer 2 – Comment 2**	**(R2.2)**
Calibration underlying the validation (Fig. 1; Extended Data Table 2; Methods). Extended Data Table 2 reports RMSEs for the individually calibrated models, whereas the projections use the cross-calibrated configuration. The Fig. 1 caption does not state which configuration it shows, and the skill scores at lines 110–112 are unattributed. Since the argument runs from validation to projection, please state the calibration explicitly in both captions and in the Methods, and report the glacier-wide skill scores for the cross-calibrated configuration if they differ. Both parameter sets already exist.	

We thank the reviewer for pointing out this ambiguity. Both Fig. 1 and Extended Data Table 2 are based on the cross-calibrated parameter configuration, which is the standard configuration used in our setup and the one applied for the projections. We have now clarified this explicitly in the relevant figure and table captions, as well as in the Methods section.

Specifically, we added to the Fig. 1 caption, line 127:

"... using the cross-calibrated parameter configuration."

In the Methods section, lines 490-492, we added:

"The validation is performed using the cross-calibrated daily and monthly GloGEM configurations that are also used for the projections (see section Calibration consistency across temporal resolutions)."

Finally, in the caption of Extended Data Table 2, line 737-740, we clarified:

"RMSE Da. and RMSE Mo. denote the root mean square error, MAE Da. and MAE Mo. the mean absolute error, and R^2 Da. and R^2 Mo. the coefficient of determination for the cross-calibrated daily and monthly model configurations, respectively."

The glacier-wide skill scores reported in the main text therefore correspond to the cross-calibrated configuration used for the projections.

**Reviewer 2 – Comment 3**	**(R2.3)**

Russian Arctic in the abstract (lines 25–27). The abstract now names the Russian Arctic and Svalbard as the regions with relative increases of up to 25%. Svalbard is well supported (14 glaciers, 270 observations). The Russian Arctic rests on one glacier and five observation-years, and the monthly model has the marginally lower RMSE there; the manuscript notes at lines 119–121 that the region allows only limited evaluation. Please lead with Svalbard, or qualify the Russian Arctic. New Zealand (lines 313–315) and the Low Latitudes are similarly weakly constrained and may deserve brief acknowledgement.

We thank the reviewer for this helpful comment. We agree that the projections for the Russian Arctic are associated with higher uncertainty, given the very limited number of available validation observations in this region. We have therefore removed the explicit reference to the Russian Arctic from the abstract and now only refer to Svalbard, which is better supported by the observational record. The revised text on lines 25-27 now reads:

"We show that accounting for short-term meteorological variability leads to systematically greater projected glacier mass loss, with relative increases over 2025-2100 of up to 25% in Svalbard."

We also agree that New Zealand is similarly weakly constrained, with only one glacier available for validation. This is mentioned together with the Russian Arctic on lines 101-103:

"Regions with sparse mass-balance observations, such as the Russian Arctic and New Zealand, provide only limited opportunities for robust model evaluation, highlighting the continued need for long-term in situ observations in underrepresented glacier regions."

For the Low Latitudes, four glaciers are available for validation. We therefore chose not to explicitly mention this region in the same sentence, while still acknowledging more generally that observational coverage remains uneven across glacier regions.

Reviewer 2 – Comment 4	(R2.4)

Comparison at lines 569–572. The 20–33 mm daily-minus-monthly difference is set against the 18 mm glacier contribution observed over 2000–2023. These are different quantities: a spread between two model configurations, and a measured historical signal. Please reframe the comparison, for instance by noting that the difference falls within the GCM ensemble spread shown in Fig. 4a, or omit it.

We agree that the daily-minus-monthly difference and the observed historical glacier contribution represent different quantities. We have therefore removed the comparison with the 2000-2023 observed glacier contribution and now instead frame the daily-minus-monthly difference as requested relative to the GCM ensemble spread shown in Fig. 4a. The text in lines 411-415 now reads:

"By 2100, our daily model projects a global glacier contribution of 86–157 mm to global mean sea-level rise, exceeding monthly estimates by 20–33 mm (Fig. 4a,b). This additional contribution in the daily model is substantial, although it remains within the GCM ensemble spread shown in Fig. 4a."

Reviewer 2 – Comment 5	(R2.5)

Extended Data Table 2. Please report MAE and R^2 alongside RMSE. The daily model's claimed advantage lies in interannual variability, and R^2 is already quoted globally in the main text.

We thank the reviewer for this suggestion. We have added the MAE and R^2 values for both the daily and monthly model configurations to Extended Data Table 2, alongside the previously reported RMSE values. This provides a more complete evaluation of model performance, including the representation of interannual variability.

Reviewer 2 – Comment 6	(R2.6)

Extended Data Fig. 1 caption, and lines 90–92. The caption describes a fixed standard deviation of 5 °C without indicating that this is illustrative, and the main text describes a "fixed" standard deviation, which reads as spatially uniform rather than stationary in time. The Methods wording at lines 727–731 is clear and could be adopted in both places.

We thank the reviewer for pointing out this ambiguity. We agree that the previous wording could be interpreted as implying a spatially uniform standard deviation, whereas the intended meaning was that the sub-monthly temperature variability is prescribed from historical daily temperature variability for each month. We have therefore revised both the main text and the caption of Extended Data Fig. 1 to make this clearer.

In the main text, we modified the text on lines 79–84 to now read:

"In contrast, in the standard monthly GloGEM configuration, surface mass balance is computed from monthly mean temperature and total monthly precipitation, with precipitation phase based on monthly temperature and melt estimated using a statistical approach that prescribes sub-monthly temperature

variability through a monthly standard deviation of daily temperatures derived from historical data[16,36] (see Methods)."

In the caption of Extended Data Fig. 1, line 786, we now explicitly state that the value shown is illustrative:

"… an illustrative standard deviation of 5°C"

**Reviewer 2 – Comment 7**	**(R2.7)**
Data and code availability (lines 950–955). The text directs readers to GitHub, but reference 83 is a Zenodo software DOI and no repository URL is given; please add it so the documentation is reachable. A reserved DOI for the simulation outputs, cited now, would complete this.	

We thank the reviewer for pointing this out. We have now added the GitHub repository URL to the Data and code availability section. The revised text on lines 701-703 now reads:

As part of this study, we have made a fully documented version of GloGEM publicly available on GitHub [https://github.com/eth-vaw-glaciology/GloGEM][83].

We also prepared the simulation outputs for Zenodo and included the corresponding DOI.

"Van Tricht, L., Huss, M., Zekollari, H., van Tiel, M., von der Esch, A., Beer, J., and Farinotti, D.: Resolving short-term meteorological variability amplifies projected glacier loss and sea-level rise, Zenodo [data set], https://doi.org/10.5281/zenodo.21792181, 2026"

Reviewer 3

**Reviewer 3 – Comment 1**	**(R3.1)**
The authors have adequately addressed my comments	

We thank the reviewer for their positive assessment and are pleased that the revised manuscript adequately addresses their comments.

“Resolving short-term meteorological variability amplifies projected glacier loss and sea-level rise” submitted by Van Tricht et al.

Main comment

This manuscript recalculates global glacier mass-balance variability using a daily time step instead of the previously employed monthly resolution. In doing so, precipitation, degree-day factors (DDF), and air temperature are calibrated so as to reproduce the 2000–2019 mass balances derived from the DEM. The authors then perform future projections and classify the causes of regional differences between the monthly and daily models into four types, which they discuss in detail. Glacier runoff is also computed. Their calibration procedure follows that of Huss and Hock (2015), with only a slightly different calibration range. The main differences from previous studies lie in the input datasets used. Specifically, the authors use ERA5 instead of ERA-Interim as the meteorological forcing, and they update the mass-balance data used for calibration from Gardner et al. (2013) to Hugonnet et al. (2021).

In the three-step calibration procedure, I understand that T_{bias} is initially set to zero when C_{prec} is determined, but from the Methods section it was not clear how the DDF is specified at this stage. If the procedure is identical to Huss and Hock (2015), i.e. a spatially uniform DDF is assumed, a key issue is that the calibrated C_{prec} will strongly depend on this initial choice of DDF.

With the current approach, DDF is only allowed to vary when C_{prec} becomes smaller than 0.8 or larger than 2.5. DDF, however, typically varies by about a factor of two between regions. This implies that for the majority of glaciers (those with $0.8 \leq C_{prec} \leq 2.5$) a single constant DDF value is effectively used. In my view it is not clear whether such a configuration can adequately reproduce the observed mass-balance patterns for future projection.

In the manuscript, in order to “obscure the interpretation of temporal-resolution effects”, the authors average C_{prec} and T_{bias} between the monthly and daily models and then recompute the DDF for each temporal resolutions. However, I wonder whether this averaging step actually hides potentially important information. Before averaging, it would be very informative to explicitly analyse and discuss the differences in the individually calibrated parameters C_{prec} and T_{bias} between the daily and monthly setups. I think This could help clarify how much of the model behaviour stems from temporal resolution itself versus from compensating changes in the calibrated parameters.

In my view, the calibration strategy proposed in this manuscript is not particularly innovative, and the resulting model does not seem well suited to reveal the fundamental processes governing glacier change.

Specific comments

・There are several places in the manuscript where the reference numbers do not seem to be correctly assigned.

Lines 98–109: When moving from monthly to daily data, the authors only demonstrate cases where the agreement with observations improves, but Fig. 1 suggests that there are also regions and elevation bands where the fit does not improve. Focusing on such regions and bands and identifying the reasons for the degraded performance could, in my view, reveal important shortcomings of the current calibration strategy.

Line 132: What exactly is meant by “original glacier outline” here? Is the term “original” actually necessary?

Fig. 3: For the graphs within the “patterns” panels of Fig. 3, the right-hand y-axis is labeled “Diff vs 2025 (%)”, but it is not clear what this difference is taken between. Please explicitly state what is subtracted from what (e.g. “daily minus monthly relative to 2025” or similar) so that the definition of the percentage difference is unambiguous.

Fig. 3e: I found Arctic Canada South here, and not Alaska, Scandinavia and New Zealand.

Lines 188–240 (“Regional and global differences in projected ice volume changes” section): In this section, the authors discuss projected changes in glacier ice volume, which inherently include changes in glacier area. However, most of the mechanisms invoked to explain the regional differences relate to surface energy or mass-balance processes at the glacier surface. I therefore wonder whether the manuscript would be clearer if the authors first focused on discussing differences in surface mass balance alone, before moving on to the resulting differences in ice volume change (which additionally involve geometric adjustment and area change).

L211-213 “where resolving daily variability produces steeper mass balance” In the Extended Figure 2, New Zealand has not larger mass balance gradient in the daily model.

Line 317: Is the expression “snowlines or snow cover datasets” really appropriate here? These datasets only provide information on the areal extent of snow cover, not on its mass. Wouldn’t snow water equivalent (SWE) be more relevant for this purpose?

Line 452: The statement “applying distinct degree-day factors (DDFs) for snow, firn and ice, with fixed ratios of 1:1.5:2, respectively” requires a supporting reference.

Lines 484–494: It would be important to explicitly state in which regions (or for which glaciers) a temperature adjustment was necessary. In addition, the manuscript lacks a clear description of which specific mass-balance quantity the three parameters were calibrated against (e.g. glacier-wide mean annual balance versus elevation-band mass balance). This is a crucial methodological detail and should be described more explicitly.

Manuscript ID: NCOMMS-26-026192-T

Manuscript Title: Resolving short-term meteorological variability amplifies projected glacier loss and sea-level rise

Summary

This manuscript presents the first global, scenario-based glacier evolution projection using a daily-resolution version of the Global Glacier Evolution Model (GloGEM), applied to all glaciers larger than 5 km^2 (~94% of global ice volume). The authors compare daily and monthly model configurations under three SSP scenarios (SSP1-2.6, SSP3-7.0, SSP5-8.5) forced by five CMIP6 GCMs, and quantify the implications of temporal resolution for projected mass balance, glacier runoff, ice volume evolution, and sea-level rise. The central finding is that resolving daily meteorological variability leads to systematically greater projected glacier mass loss (5–10% more by 2100), with regional relative increases exceeding 25%, contributing an additional 20–33 mm to global mean sea-level rise compared to monthly forcing.

The topic is timely and relevant to the glaciological, hydrological, and climate impact communities. The cross-calibration approach to isolate the temporal-resolution effect is methodologically thoughtful, and the identification of four distinct regional divergence patterns offers physical insight beyond a simple aggregate comparison. The validation against WGMS and Glambie datasets is appropriate, and the demonstration of improved performance during extreme years (e.g., 2022 Alpine melt, 2024 Svalbard) is compelling.

Nevertheless, several methodological choices, interpretational claims, and reproducibility concerns require attention before the manuscript is suitable for publication. The headline performance improvements in routine years are modest, the comparison may inadvertently treat the monthly model as a strawman, and several quantitative claims (particularly the sea-level rise extrapolation to flood exposure) require more careful framing. I therefore recommend major revision, with the major and minor comments detailed below.

Major Comments

Major Comment 1: The cross-calibration design may not cleanly isolate the temporal-resolution effect

The authors implement a cross-calibration procedure (lines 505–524) in which C_prec and T_bias are independently calibrated for both configurations, then their *mean* values are imposed on both daily and monthly simulations, with only the DDFs subsequently re-calibrated separately. The stated goal is to isolate the impact of temporal resolution. However, this design raises several concerns:

1. **The averaged parameters are optimal for neither configuration.** By construction, the resulting C_prec and T_bias are a compromise that does not reflect the best-fit calibration for either model. This means the reported daily-vs-monthly differences may partly reflect

the *imposition of suboptimal parameters* on one or both configurations, rather than purely the resolution effect.

2. **No sensitivity analysis is provided.** It would be valuable to know how results differ when each configuration is independently calibrated (the "natural" approach) vs. the cross-calibrated approach. Without this comparison, readers cannot assess whether the headline conclusions are robust to the calibration design.
3. **DDFs are re-calibrated, but C_prec is not, despite precipitation phase being central to several mechanisms.** For the Iceland "reversed divergence" pattern (lines 232–241), the authors argue that monthly averaging overclassifies snowfall and biases calibration. If this is true, then *fixing* C_prec at an averaged value may carry over the bias from the monthly calibration into the daily configuration.
4. **The Iceland mechanism appears post-hoc.** The argument that monthly averaging "overclassifies snowfall, and thus overestimates accumulation. This bias propagates into calibration, yielding parameters that increase glacier mass-balance sensitivity to future warming" is plausible but unsupported by direct diagnostic evidence. The authors should provide a quantitative breakdown of the calibrated parameters for Iceland under both configurations (independent calibration), and ideally for at least one region from each of the four divergence patterns.

Recommended actions: (i) Add a supplementary analysis comparing results from independently-calibrated daily and monthly configurations against the cross-calibrated baseline. (ii) Provide regional summary tables of calibrated parameters (C_prec, T_bias, DDF_snow) for both configurations. (iii) Explicitly discuss the calibration-design trade-offs in the Methods section, including how Iceland's parameters differ between independent and cross-calibration.

Major Comment 2: The monthly model baseline may function as a strawman, weakening the central claim

The authors propose that daily modelling captures processes that "a monthly resolution cannot reproduce" (line 290). However, several aspects of the monthly model comparison appear to penalise it more than is necessary:

1. **Fixed sub-monthly temperature standard deviation of 5°C.** The authors themselves note (lines 308–311) that "assumptions of stationary sub-monthly temperature variability are increasingly inconsistent with observed changes in the variance of temperature distributions under climate change." Yet this stationary σ is retained as the basis for comparison. If σ were updated using GCM-derived sub-monthly variability (which is, in principle, available from the same daily GCM outputs used to force the daily model), how much of the daily-monthly gap would close?
2. **Precipitation phase based solely on monthly mean temperature in the monthly model.** Extended Data Fig. 1 vividly illustrates the issue: in a warm month with mean T > 0 °C, the monthly configuration yields *zero snowfall*. This is a known limitation that simpler statistical adjustments (e.g., applying the same temperature PDF to precipitation-phase partitioning) could partially address. The comparison would be more compelling if it

included an "improved monthly" intermediate configuration that applied the sub-monthly PDF to precipitation phase as well.

3. **Monthly surface-type updating is a model choice, not an intrinsic property of monthly forcing.** A monthly-forced model could conceptually update surface type more frequently using sub-monthly logic. The fact that the standard GloGEM does not is a structural choice that compounds the resolution penalty.

The combined effect is that some of the reported daily-vs-monthly differences may reflect *implementation choices* in the monthly model rather than a fundamental limitation of monthly forcing. The authors note this implicitly (lines 308–315) but do not test it.

Recommended actions: (i) Implement and report at least one "improved monthly" sensitivity experiment in which the monthly precipitation phase uses the assumed sub-monthly temperature PDF (matching the PDD calculation). (ii) Discuss explicitly the difference between the "resolution effect" (temporal granularity of inputs) and the "implementation effect" (specific choices in the monthly GloGEM). (iii) Soften the language in the abstract and conclusions to reflect that the comparison is between the daily GloGEM and the *current standard* monthly GloGEM, not a generic monthly model.

Major Comment 3: Validation evidence does not equally support all regional claims; high-latitude conclusions rest on limited in situ data

The mass-balance validation against WGMS observations (Fig. 1) shows overall modest improvement (glacier-wide RMSE 0.63 → 0.61 m w.e., R^2 0.71 → 0.75). The authors emphasise that the daily model substantially outperforms in extreme years (RMSE 0.98 → 0.83 m w.e. for ±2σ events), which is convincing.

However, the largest projected divergences between daily and monthly models occur in high-latitude, ice-cap-dominated regions: Svalbard, Russian Arctic, and Antarctic/sub-Antarctic Islands (Fig. 3f; up to 20–25% relative difference by 2100). These regions are precisely where in situ WGMS validation is sparse:

1. **The WGMS dataset is acknowledged to be "biased towards smaller, mid-latitude glaciers"** (line 401–402). This means the regions where the daily model claims the *largest* improvements are validated the *least* directly.
2. **The Svalbard 2024 example is compelling but anecdotal.** A single extreme year for a single region does not constitute a robust validation of a ~20–25% projected divergence over the 21st century in the broader Russian Arctic and Antarctic regions.
3. **The Antarctic/sub-Antarctic anomaly is concerning.** The authors note (lines 261–265) that both daily and monthly models project *smaller* losses than previous global assessments in these regions, and attribute this to "forcing and model uncertainty [that] can outweigh temporal-resolution effects in cold, low-melt regimes." If this is so, then the claimed 20–25% divergence in Antarctic/sub-Antarctic glaciers under SSP5-8.5 should be presented

with greater caution — the daily model's apparent improvement in these regions may rest on a forcing baseline that is itself biased.

4. **Cumulative regional mass balance comparison (Fig. 1c)** uses Glambie, which is an inverse comparison: simulations restricted to glaciers >5 km² are compared to observations including all glaciers. This methodological mismatch could mask or amplify apparent differences and should be discussed.

Recommended actions: (i) Add a regional breakdown of validation statistics (RMSE, MAE, R^2) for each RGI region for both models, with the number of validation glaciers and observation-years per region. (ii) Explicitly flag the regions where validation data are sparse (Russian Arctic, Antarctic/sub-Antarctic) and temper claims accordingly. (iii) For the Antarctic/sub-Antarctic anomaly, provide an explicit discussion of whether the projected daily-vs-monthly divergence is robust given the underlying baseline mismatch with literature. (iv) Where possible, supplement validation with remote-sensing-based seasonal snow line observations (the authors mention these in lines 315–317 as a future opportunity; integrating even a few representative examples would strengthen the high-latitude case).

Major Comment 4: The sea-level rise to flood exposure extrapolation overstates implications and should be revised

In the final section (lines 325–334), the authors translate the additional 20–33 mm of sea-level rise from daily-vs-monthly modelling into "4–10 million additional people exposed to flooding by 2100" using a linear extrapolation of ~200,000–300,000 people per mm of SLR. This claim warrants substantial revision for several reasons:

1. **Linear extrapolation is unlikely to hold at this scale.** The cited exposure rate (Kulp & Strauss, 2019; Nicholls et al., 2021) is derived from current population distributions and present-day coastal topography. Applying it to a 20–33 mm increment *on top of* a much larger projected SLR (the daily model's 86–157 mm glacier contribution alone, plus thermal expansion, ice sheets, and land water storage) ignores that exposure responses to SLR are typically nonlinear and depend on local topography, adaptation, demographic change, and concurrent processes such as subsidence.
2. **The marginal vs. total framing is misleading.** Stating that "an additional 4–10 million people are exposed to flooding by 2100" implies that this is a discrete, attributable population. In reality, this is a marginal sensitivity estimate within a much larger uncertainty envelope. The comparison to the 18 mm of glacier-driven SLR observed over 2000–2023 (line 328) is rhetorically powerful but mixes a *modelling choice difference* with an *observed historical increment*, which are not directly comparable.
3. **The framing risks overshadowing the methodological contribution.** The paper's strongest contribution is methodological — demonstrating that temporal resolution materially affects projections. The flood-exposure extrapolation pushes into impact-assessment territory that requires additional caveats not provided here.

4. **Uncertainty quantification is missing.** No uncertainty range is given for the exposure estimate, despite the underlying SLR estimate having a ±1 SD spread that overlaps zero in some scenarios (Fig. 4a uncertainty envelopes).

Recommended actions: (i) Either remove the flood-exposure extrapolation or substantially temper it. If retained, present it explicitly as a first-order sensitivity estimate, include uncertainty bounds, and acknowledge nonlinearities and adaptation. (ii) Reframe the comparison to historical glacier-driven SLR (line 328) to avoid conflating a model-choice difference with an observed increment. (iii) Consider moving impact extrapolations to a discussion paragraph rather than the conclusion, where the methodological contribution should remain central.

Minor Comments

Abstract and Introduction

- **Lines 23–24:** "relative increases exceeding 25% at the regional scale". Specify which region(s) and scenario this refers to in the abstract.
- **Line 27:** "26 ± 10% under low-emission scenarios and 47 ± 17% under high-emission scenarios". State that these are ensemble-mean ± 1 SD across GCMs.
- **Lines 49–50:** The phrasing "such approaches have not yet been used for global, scenario-based projections of 21st-century glacier change" should explicitly contrast with the cited daily-forced studies (refs. 21–24) by noting their scope (regional, single-scenario, etc.) for clarity.
- **Line 55:** "transient albedo feedbacks"? Given that GloGEM uses a degree-day approach without explicit albedo, clarify how the daily model captures albedo effects (presumably indirectly through surface-type switching).

Methods

- **Lines 64–65:** Justify the 5 km² threshold more quantitatively. What fraction of regional ice volume is excluded in each RGI region? This is particularly relevant for regions dominated by smaller glaciers (e.g., Central Europe, New Zealand, Low Latitudes).
- **Lines 363–364:** Specify whether the "nearest land grid cell" approach causes systematic biases for glaciers near coastlines or in steep topography.
- **Lines 382–383:** The delta-method bias correction over 1991–2020. Does this preserve daily variability statistics (variance, autocorrelation, extreme tails) or only the mean? This is critical given the manuscript's focus on extremes.
- **Lines 422–423:** The 0.5–2.5 °C snow–rain transition is treated as a fixed range. Recent work (cited as ref. 26, Jennings et al. 2025) suggests this range varies regionally. A brief acknowledgment would be appropriate.
- **Lines 466–470:** "Processes such as basal melt, debris cover and internal accumulation are not included". This omission is more consequential for some regions (e.g., debris cover in High Mountain Asia). Briefly discuss whether this could systematically affect the daily-vs-monthly comparison in those regions.

- **Lines 530–532:** The computational cost statement ("approximately 30 times higher") is a useful figure. Provide the absolute runtime (CPU-hours) for the daily and monthly global runs to help readers calibrate feasibility for their own work.

Results – Mass Balance (Figure 1)

- **Figure 1a:** The grey shaded band (±2σ of observations) is helpful but the colour distinction between daily (orange) and monthly (blue) circles is hard to read in the dense scatter. Consider using shape differentiation in addition to colour.
- **Figure 1b (elevation-band):** The elevation-band RMSE of ~1 m w.e. is much higher than the glacier-wide RMSE (~0.6). Briefly explain to readers why elevation-band errors are inherently larger.
- **Figure 1c:** The Central Europe 2022 highlighted point is effective. Consider adding 1–2 other named extreme years (e.g., Svalbard 2024, Western Canada 2023) to make the extreme-year argument more visually concrete.

Results – Runoff (Figure 2 and Extended Data Figs. 3–5)

- **Lines 142–144:** "high-flow glacier runoff in Western Canada and the USA is projected to occur ~46 days earlier by 2100". Please clarify whether this is the daily model under SSP5-8.5 vs. the 2001–2020 baseline (which I infer but is not explicit).
- **Figure 2 panel labels:** The figure caption refers to panel "f" but I see only panels a–e in the description. Please verify the panel labelling matches the figure.
- **Extended Data Fig. 4:** The +8% (2022) and –12% (2014) comparison is striking but represents only two years. Provide the full distribution of daily-vs-monthly annual runoff differences for Central Europe over 2000–2024 to support the generality of the claim.
- **Lines 171–173:** "ice melt accounts for approximately 70–80% of present-day glacier runoff by late summer (September), whereas the monthly model yields a substantially lower contribution of 44%". This is a large difference. Is it possible to validate against in situ HMA hydrological observations (e.g., isotope-based partitioning studies)?

Results – Ice Volume (Figure 3, Extended Data Fig. 6, Extended Data Table 1)

- **Figure 3a–c (causes):** The schematic panels are useful but could be more clearly linked to the right-hand patterns. Consider adding small icons or coloured borders matching each pattern to the relevant divergence panel (d–g).
- **Extended Data Table 1:** The "Global" row shows -24 ± 9% (SSP1-2.6, daily) vs. -21 ± 8% (monthly). The text reports "26 ± 10% (SSP1-2.6) and 47 ± 17% (SSP5-8.5)". The signs and values differ slightly. Please reconcile (likely a 2025 vs. 2015 reference year issue, but should be made explicit in the table caption).
- **Extended Data Table 2:** The comparison to Zekollari et al. (2024) is valuable, but the monthly model in this study yields *less* loss than the previous GloGEM CMIP6 results (e.g., –32 ± 12% vs. –39 ± 21% for SSP3-7.0 by 2100). The text notes this (lines 277–278) but does not explain *why*, given that both are monthly GloGEM with similar forcing, this discrepancy deserves a sentence or two of discussion. Is it due to ERA5-Land vs. ERA5, the updated calibration, 2015 vs. 2025 baseline, or the >5 km² subsetting?

Results – Global (Figure 4)

- **Figure 4b:** The diverging y-axes (volume difference on left, SLR difference on right) are slightly hard to parse. Consider stacking the panels or using a single normalised axis.
- **Lines 273–275:** "the divergence ... does not stabilise by 2100 ... suggesting that differences may continue to grow beyond 2100". This is a strong claim. Either run a short extension (e.g., to 2150) or temper the statement.

Discussion

- **Lines 287–290:** The opening of the discussion is somewhat repetitive of the abstract. Consider restructuring to emphasise the *implications* rather than re-summarising findings.
- **Lines 296–298:** "Many coupled applications require temporally consistent runoff estimates and realistic interannual variability, particularly in regions where glacier melt sustains dry-season water supply and hydropower production." It is a strong statement; cite the relevant glacio-hydrological coupling literature explicitly here (refs. 22–24, 32–35 are cited earlier but the link to this conclusion could be tighter).
- **Lines 308–319:** This paragraph on pathways for improving monthly models is excellent and should be expanded. Specifically, the suggestion that snowline observations could constrain models (ref. 55 (actually ref. 54)) is intriguing. Could the authors comment on whether their daily model could be calibrated/validated this way in future work?

Data and Code Availability

- **Lines 572–577:** The statement that "The GitHub version is currently undergoing final evaluation, with a public release planned for late spring alongside full documentation" is concerning for a Nature Communications submission, which operates under transparent peer review. Code should be available at the time of review to allow independent assessment. Please provide at minimum a private repository link (or anonymised code archive) to the reviewers and editors during the revision.
- **Simulations output data:** Specify the repository (e.g., Zenodo, PANGAEA) and provide a placeholder DOI if available.

References and Citations

- **Reference 51 (Screen, 2014):** This is cited at line 275 ("does not stabilise by 2100, suggesting that differences may continue to grow") but ref. 51 is about Arctic amplification reducing temperature variance, the connection is unclear. Verify the citation.
- **Reference numbering inconsistency:** Ref. 47 (Aguayo et al. 2024 on Patagonia uncertainty) appears in the reference list but I could not locate its in-text citation. Please verify. The in-text numbering skips Reference 47 entirely, jumping directly from citation 46 (line 238) to citation 48 (line 257).
- **Line 102:** Footnote-style reference to "the daily model[1]". ref. 1 is the Glambie Team paper, which is the observational dataset. The phrasing is potentially confusing; consider rephrasing as "compared to Glambie observations[1]."

- **Line 317:** Reference 55 is actually a paper on coastal flooding by Nicholls et al., while Reference 54 is the correct paper on transient glacier snowlines by Loibl et al.

Figures and Extended Data (General)

- Several Extended Data figures (e.g., ED Fig. 2 mass-balance gradients) could benefit from including the sample size (number of glaciers) per region directly on the plot.
- Extended Data Fig. 7 is a useful comparison but the labelled outliers (Svalbard and J.M., Russian Arctic in SSP1-2.6 2100 panel) are not discussed in the caption. A brief note on what drives these outliers would help readers.

Language and Presentation

- **Line 30:** "an additional 20-33 mm to global mean sea-level rise": consistently use either en-dashes or hyphens in numerical ranges throughout (multiple inconsistencies exist).
- **Line 53:** "limited number of large-scale glacier evolution models have been forced with daily data", here "small number" reads more naturally than "limited number."
- **Line 165:** "snouth" should be "snout" (line 551 in Methods).
- **Lines 215–216:** "relative losses that are typically 2–5% higher in the daily model". Clarify whether this is the relative difference in remaining volume, or the absolute percentage-point difference in projected loss.
